# Unusual Magnetic Field Responsive Circularly Polarized Luminescence Probes with Highly Emissive Chiral Europium(III) Complexes

**DOI:** 10.1002/anie.202012133

**Published:** 2020-11-03

**Authors:** Junhui Zhang, Lixiong Dai, Alexandra M. Webster, Wesley Ting Kwok Chan, Lewis E. Mackenzie, Robert Pal, Steven L. Cobb, Ga‐Lai Law

**Affiliations:** ^1^ Department of Applied Biology and Chemical Technology State Key Laboratory of Chemical Biology and Drug Discovery The Hong Kong Polytechnic University Hung Hom, Hong Kong SAR China; ^2^ The Hong Kong Polytechnic University Shenzhen Research Institute Shenzhen 518000 P. R. China; ^3^ Department of Chemistry Durham University South Road Durham DH1 3LE UK

**Keywords:** chirality, circularly polarized luminescence, lanthanide, magnetic properties

## Abstract

Chirality is ubiquitous within biological systems where many of the roles and functions are still undetermined. Given this, there is a clear need to design and develop sensitive chiral optical probes that can function within a biological setting. Here we report the design and synthesis of magnetically responsive Circularly Polarized Luminescence (CPL) complexes displaying exceptional photophysical properties (quantum yield up to 31 % and |g_lum_| up to 0.240) by introducing chiral substituents onto the macrocyclic scaffolds. Magnetic CPL responses are observed in these chiral Eu^III^ complexes, promoting an exciting development to the field of magneto‐optics. The |g_lum_| of the ^5^D_0_ → ^7^F_1_ transition increases by 20 % from 0.222 (0 T) to 0.266 (1.4 T) displaying a linear relationship between the Δg_lum_ and the magnetic field strength. These Eu^III^ complexes with magnetic CPL responses, provides potential development to be used in CPL imaging applications due to improved sensitivity and resolution.

## Graphical abstract

A series of chiral, water‐soluble, DO3A‐based Eu^III^ complexes was designed and synthesized. The chiral substituents introduced onto the macrocyclic scaffolds enhanced the photophysical properties with quantum yields up to 31 % and |g_lum_| up to 0.240. The |g_lum_| value increased by 20 % from 0.222 to 0.267 under external magnetic field showing a linear response.

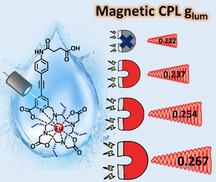

## Introduction

Chirality plays an essential role in all living matter, it can be observed from macroscopic to microscopic worlds, from human hands to natural amino acids. It exists in biological activities, including cellular uptake processes, metabolism and in protein structures. Significant efforts have been devoted to reveal its underlying existence in nature. To date, methodologies involving circular dichroism (CD) and circularly polarized luminescence (CPL) have proven to be valuable techniques that can be used to probe chiral information.[^1^, ^2^] CPL and magnetic circularly polarized luminescence (MCPL), which are the emission analogs of CD and magnetic circular dichroism (MCD) respectively, provide a powerful and highly sensitive way to determine the conformation of biological macromolecules in solution and hence give a better understanding of their activities.[^3^, ^4^, ^5^] The advantage that CPL offers over CD is that it can provide chiroptical information of the excited states of compounds to correlate with the local chiral structural changes dynamically.^[6]^ For example, CPL studies have been used to discriminate the ratio of ADT/ATP in solution.^[7]^ The diversity in the use of CPL has been further stimulated by recent technological developments such as chiral luminescence microscopy.[^8^, ^9^] The challenge now lies in the development of chiral complexes with both high CPL and quantum yields that are suitable for in vitro studies.

Due to the selection rules for CPL, which is reliant on magnetic dipole‐allowed transitions, an innate feature of some transitions in lanthanides luminescence, large CPL signal can be observed in certain transitions of lanthanide complexes. According to theoretical studies, the relationship between the g_lum_ and the lanthanide transitions can be described as g_lum_=4|M_ba_|/|P_ab_| cos*τ*_ab_,^[10]^ where |P_ab_| and |M_ba_| are the electric and magnetic dipole transition moment vectors, respectively, hence the most suitabletransition to give a large magnitude for a strong dissymmetry factor (g_lum_) is at the magnetic‐ dipole allowed, but electric‐dipole forbidden ^5^D_0_ → ^7^F_1_ transition.[^10^, ^11^, ^12^] Compared to small chiral organic molecules (|g_lum_| typically within 10^−3^–10^−1^ range),[^13^, ^14^, ^15^] typical g_lum_ of chiral lanthanide complexes can reach ≥0.1.[^11^, ^16^, ^17^, ^18^, ^19^] In addition to distinguished g_lum_ values, the sharp and narrow signature emission bands, long lifetimes and large pseudo‐Stokes’ shifts of the chromophores, as well as the lanthanide ions’ spherical nature which eliminates the problem of anisotropy,^[20]^ are all factors that attributes to lanthanide complexes being ideal for use in imaging applications. However, to date, there are still only limited examples of lanthanide complexes used in CPL applications, due to the difficulties in the design and synthetic work.

**Scheme 1:**
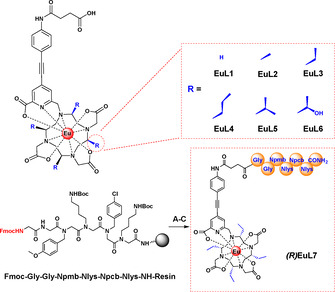
Molecular structures of **EuL1**–**6** (top). Peptoid used for conjugation for **EuL7** (bottom), A) piperidine/DMF (1:5, v/v); B) DMF, **(*R*)(SAP)EuL3**, NMM, PyBop; C) 95 % TFA, 3 % water, 2 % TIPS.

Herein, we report a new series of highly emissive, CPL europium probes, **EuL2**–**7**, with excellent stability and water solubility rendering it suitable to the development of biological sensors (Scheme 1). In our ligand design, chiral substituents were introduced to the macrocyclic scaffolds, achieving 4 chiral centers, which suppress ring inversion and “lock” the isomers in place, eliminating interconversion between isomers. By the use of a reversed‐phase HPLC equipped with an achiral column, these geometric isomers can be easily separated and hence reduces the difficulties in the purification and synthetic methodology. The selection of a suitable rigid chromophore and effective sensitiser was performed through the screening strategy reported in our prior work.^[21]^ The quantum yields of **EuL2**–**7** improved significantly compared with the achiral **EuL1** due to the modified chiral DOTA chelators.^[22]^

More importantly, other than the intrinsic CPL properties, we further examined the effect of an external magnetic field on our compounds. This work builds on earlier publications of Riehl and Richardson, both of them studied the induced MCPL under static external magnetic field from naturally optically inactive probes, and lately by Yoshikawa et al.[^23^, ^24^, ^25^] As postulated, applying an external magnetic field induced stronger CPL signals and higher g_lum_ values were obtained for **EuL2**–**7** with obvious trends in MCPL enhancement, making these Eu^III^ complexes suitable as magneto‐optical probes.[^26^, ^27^]

With these promising results, we attempted to enhance the biocompatibility of these Eu^III^ complexes. As a proof of concept, a cell penetrating peptoid (CPPo) was introduced to the carboxylic linker handle on the chromophore. This is use to show the functionalisation capabilities for developing more robust and specific probes that can be used for tracking specific cellular organelles in biological imaging.^[28]^ Upon successful peptoid conjugation, we found this led to an even higher quantum yield, and at the same time demonstrates the feasibility and potential of these Eu^III^ complexes as imaging probes and tags where functionalisation does not sacrifice the desirable photophysical properties.

## Results and Discussion

**EuL1**–**6** (**(*R*)EuL3** and **(*R*)EuL7)**) are in *S*(*R*) configuration and synthesized from the *S*(*R*) isomer of natural amino acids. Unless stated, the handedness of Eu^III^ complexes is in *S* configuration. The synthesis of chiral cyclens with methyl or ethyl substituents was reported in our previous publication.^[22]^ The other chiral cyclens were also synthesised by using the same strategy with catalysis of the 3‐membered ring intermediates by Lewis acid boron trifluoride diethyl etherate to form 12‐membered scaffolds in the main cyclization step. The configurations of the chiral substituents on all these macrocyclic scaffolds were maintained, confirmed by the crystal structure of the isopropyl chiral cyclen intermediate (Figure S74). After deprotection of these macrocyclic scaffolds, another coupling reaction was further conducted to incorporate the new chromophore—the detailed synthetic procedures are shown in the SI—this is then followed by the addition of ethyl 2‐bromoacetate to form the pendant arms on the scaffold. Subsequent deprotection of the ethyl groups on the pendant arms by LiOH gave the final ligand.

In the complexation procedure, europium chloride hexahydrate was added to the deprotected ligand **L1**–**6** in water and refluxed for 16 hours. After purification by reversed‐phase semi‐preparative HPLC, the pure SAP isomers of the Eu^III^ complexes (unless stated) were obtained for analysis and measurements.

To demonstrate that these Eu^III^ complexes are suitable for cellular studies, a CPPo, *N*pmb‐*N*Lys‐*N*pcb‐*N*Lys‐NH_2_, which can selectively localize in the mitochondria,^[28]^ was used for conjugation as proof of concept. The peptoid was conjugated via a Gly‐Gly linker to form the complex **(*R*)EuL7**. Peptoid conjugation was performed using the highly biocompatible carboxylic linker in **(*R*)(SAP)EuL3** in 3 steps (Scheme 1).

The photophysical properties of these complexes were then studied and are summarised in Table 1. For the UV measurements, **EuL1**–**7**, all complexes display a similar absorption spectrum as well as extinction coefficient in 0.1 M HEPES buffer, resulting from the same chromophore incorporated in the structure. Hence, we selected **EuL3** with the ethyl chiral backbone for discussion. The absorption band of **(SAP)EuL3** with a maximum at 333 nm (*ϵ*_350nm_ of **EuL1**–**7**: ≈18 000 mol^−1^ dm^3^ cm^−1^) (Figure S3), is assigned as the π‐π* transition of the chromophore. There is no observable ligand emission peak in the spectra of the europium complexes. This indicates efficient energy transfer of the ligand‐to‐metal and is consistent with the calculated *η*_sens_, which mostly lie within the range of 80–90 %.

**Table 1:** Photophysical properties of **EuL1**–**7** measured in 0.1 M HEPES, pH 7.3, at 298 K, excited at 350 nm, with 380 nm long pass filter.

	**EuL1**	**EuL2**	**(SAP)EuL3**	**(TSAP)EuL3**	**(*R*)EuL3**	**EuL4**	**EuL5**	**EuL6**	**(*R*)EuL7**
*Φ* [%]^[a]^	18.4±0.4	27.0±0.3	28.0±1.5	19.8±1.8	26.3±0.6	25.6±0.8	24.1±0.7	26.7±0.2	31.3±1.1
*τ* ${}_{H{}_{2}O}$ [ms]^[b]^	0.976±0.09	1.09±0.08	1.30±0.02	1.06±0.02	1.21±0.05	1.35±0.006	1.33±0.006	1.26±0.02	1.39±0.004
*τ* ${}_{D{}_{2}O}$ [ms]^[b]^	1.71±0.08	1.93±0.007	2.14±0.01	1.65±0.09	2.14±0.004	2.21±0.005	2.15±0.002	2.10±0.003	2.06±0.003
*Q* ^[c]^	0.23	0.18	0.06	0.10	0.13	0.05	0.04	0.08	−0.02
*Q* ^[d]^	0.14	0.10	−0.01	0.03	0.05	−0.02	−0.03	0.01	−0.08
*Φ* ^Eu^_Eu_ [%]	24.3±1.8	29.2±1.4	30.9±1.5	26.6±1.9	32.2±0.7	32.5±0.7	32.4±0.6	31.9±0.2	33.2±0.2
*η*_sens_ [%]	74.6±3.4	92.4±4.1	90.3±1.9	74.4±3.1	81.6±3.4	78.8±1.3	74.2±3.4	83.7±1.5	94.5±2.5
*R* ^[e]^	2.61	2.53	2.54	2.50	2.48	2.54	2.52	2.45	2.55
*B* ^[f]^	3312	4860	5040	3564	4734	4608	4338	4806	5634

[a] Relative to quinine sulfate in 0.1 M H_2_SO_4_ (*λ*_ex_=350 nm, *Φ*=0.577). Estimated errors of quantum yield and lifetime are ±15 % and ±10 % respectively. [b] Measuring the ^5^D_0_ → ^7^F_2_ transition. [c] Calculated by Parker's equation.^[29]^ [d] Calculated by Horrocks’ equation.^[30]^ [e] I(^5^D_0_ → ^7^F_2_)/ I(^5^D_0_ → ^7^F_1_).^[31]^ [f] *B*=*ϵ*_350nm_ *Φ* ^[32]^

**Figure 1:**
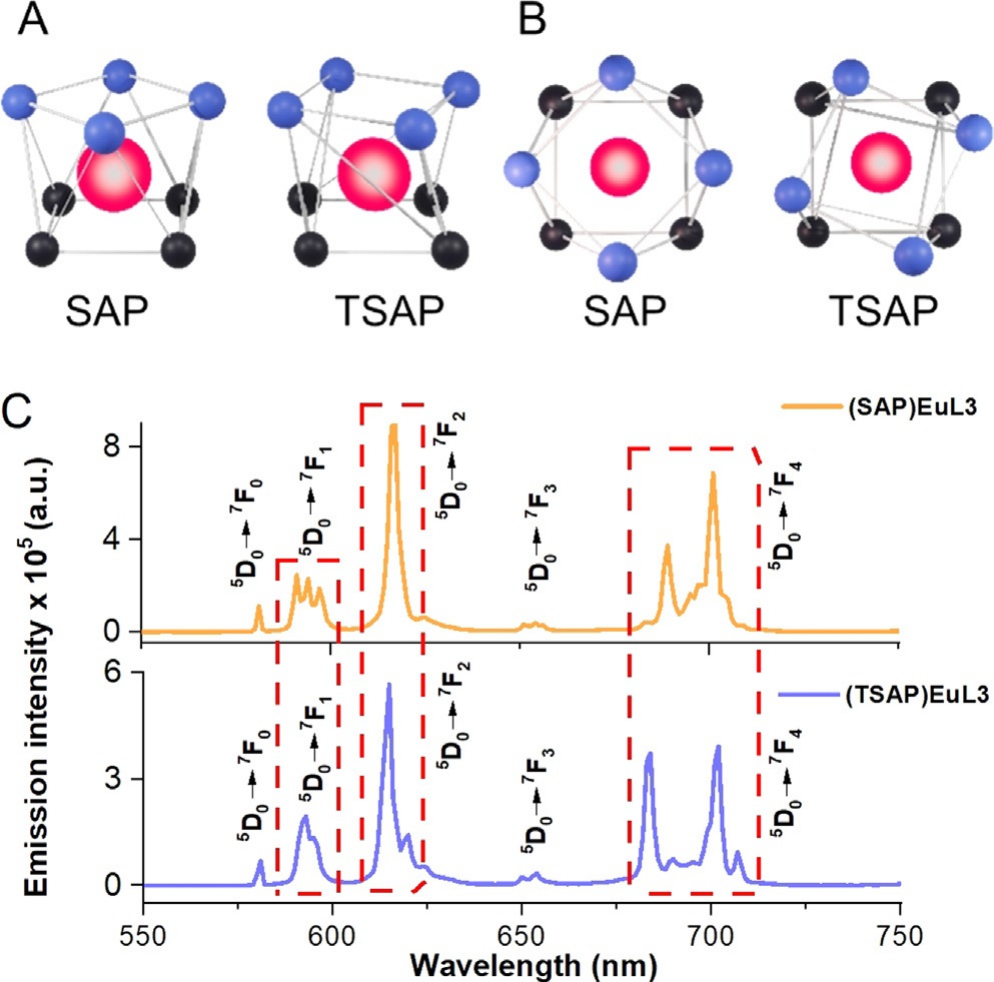
A) Front view of SAP (left) and TSAP (right); B) Top‐down view of SAP (left) and TSAP (right), atoms are represented as spheres with different color: europium (red), oxygen (blue), nitrogen (black); C) Emission spectra of **(SAP)EuL3** (top) and **(TSAP)EuL3** (bottom), excited at 350 nm, in 0.1 M HEPES buffer, pH 7.3, with 380 nm long pass filter.

According to the data in Table 1, the quantum yields of SAP isomers are generally much higher than that of the **TSAP**(**EuL3**). This may be due to the higher *η*_sens_ (80–90 %) in SAP isomers than that of the TSAP (74 %), thus giving rise to better ligand‐to‐metal energy transfer and hence higher quantum yields and brightness. Compared with the achiral **EuL1**, the chiral **EuL2**–**7** showed significant increase in quantum yields by simply varying the chiral substituent on the backbone. The only exception here is **(TSAP)EuL3**, which we propose is due to the difference in the isomer conformation (Figure 1 A & B). Upon conjugation of the peptoid to **(*R*)(SAP)EuL3**, where the ‐OH group is replaced by the peptoid to give **(*R*)EuL7**, the photophysical properties of **(*R*)EuL7** were found to be enhanced as shown by a slight increase in quantum yield when compared to the other chiral europium complexes. The luminescence lifetimes for **EuL1**–**7** were measured in water and D_2_O. Extremely long lifetimes were obtained in the millisecond range for the main Eu (^5^D_0_ → ^7^F_2_) transition. The q values of these Eu^III^ complexes are consistent, all close to 0, according to Parker's and Horrocks’ equations.[^29^, ^30^] This implies no water molecules are coordinated to the first coordination sphere of the Eu^III^ metal center. Furthermore, the chiral groups introduced to the macrocyclic scaffolds also shield the lanthanide metal center from water coordination, hence any quenching by OH oscillators is eliminated. All these factors in the structural design play an important role to attribute to the high quantum yields obtained for these Eu^III^ complexes.[^33^, ^34^] To study the energy transfer pathway, **(SAP)GdL3** was synthesized for low temperature studies at 77 K. Due to the similar ionic radii of Gd^III^ to the Eu^III^ cation, the Gd cation is commonly used as a surrogate for such measurements as it has a highly lying excited state above 30 000 cm^−1^, which typically prevents energy transfer from the ligand to the metal. Hence instead, the excited energy decay radiatively as either fluorescence from the singlet state or, due to the heavy atom effect of the proximal Gd^III^, which promotes intersystem crossing, as phosphorescence from the triplet state of the ligand.^[35]^

Insight to either a fluorescence or phosphorescence decay can be further corroborated by comparing lifetime measurements as well as comparing the emission maxima at room temperature and 77 K. From the emission spectrum of **(SAP)GdL3** at room temperature, the emission maximum is at 455 nm with a monoexponential lifetime, 7.49 μs. At 77 K, the emission maximum is at 405 nm and its monoexponential lifetime is 6.69 μs. These two emissions are assigned as ligand fluorescence from excited ^1^S_1_* and ^1^S_2_* states and are confirmed by the short microsecond lifetimes obtained. Moreover, the emission maximum at 77 K is blue shifted compared to the room temperature spectrum, this implies that energy transfer is directly from the ligand's singlet excited states, rather than the triplet states, to the Eu^III^ metal center; this phenomenal is not uncommon.^[36]^ Through unit conversion, the excited ^1^S_1_* and ^1^S_2_* states are around 21 930 cm^−1^ and 24 813 cm^−1^, which are 2904 cm^−1^ (Δ*E*: ^1^S_1_* → ^5^D_1_) and 3314 cm^−1^ (Δ*E*: ^1^S_2_* → ^5^D_2_) away from the accepting levels of Eu^III^ (Figure S15). According to the energy gap law, Δ*E* within 2500–3500 cm^−1^ is ideal for efficient energy transfer and this is consistent with the *η*_sens_ of **EuL2**–**7**.[^37^, ^38^]

As aforementioned, these Eu^III^ complexes, **EuL2**–**7** are all in the SAP form, except **(TSAP)EuL3** (Figure 1 A & B) which was isolated in order to study the properties of these geometric isomers.^[39]^ Here, the TSAP isomer existed as the minor peak, observed in the HPLC trace and were separated by an achiral reversed phase HPLC. **(SAP)EuL3** and **(TSAP)EuL3** are geometric isomers with the same chiral ethyl group in (*S*) configuration, the emission spectra (Figure 1 C) shows distinct splitting patterns for these isomers due to their different geometries in the DOTA platform. Comparing their emission spectra, the SAP and TSAP isomers can be identified through the ^5^D_0_ → ^7^F_1_, ^5^D_0_ → ^7^F_2_ and ^5^D_0_ → ^7^F_4_ transition bands. In Δ*J*=1 transition, larger spectral splitting is observed in the SAP isomer, giving rise to three well‐identifiable peaks, however a relatively broad peak was observed for the TSAP isomer. In the Δ*J*=2 transition, only one peak can be observed in the SAP isomer as the transitions caused by the SAP ligand‐field splitting are approximately at the same energy levels, but for TSAP, the energy levels of these transitions are different, which resulted in an extra minor peak near the main peak. In Δ*J*=4, a less resolved peak was observed with greater peak overlap in the SAP isomer, whereas the TSAP shows a clear defined peak separation.[^40^, ^41^] The spectral splitting in SAP and TSAP of these **(SAP)EuL3** and **(TSAP)EuL3** also shows a similar pattern to the parent chiral ethyl DOTA analogue (Figure S10). The differences of these SAP and TSAP isomers have also been confirmed by NMR spectra (see SI p. 78), which is also similar to the NMR of their parent chiral DOTA.[^22^, ^42^] Larger chemical shifts in the NMR for the proton set in SAP isomer were observed from 15 to 30 ppm, while the TSAP isomer showed relatively centralized proton signals from around 10 to 15 ppm.

To reveal the chiroptical information in biomacromolecules, such as proteins, cellular organisms, CPL compounds need to be water soluble, stable, highly emissive with large g_lum_ which is difficult to balance simultaneously.[^8^, ^11^, ^43^, ^44^, ^45^, ^46^, ^47^] In our previous studies,^[48]^ our design strategy required 8 chiral centers in order to create a chiral environment to give an optimised g_lum_ (−0.23 at the transition ^5^D_0_ → ^7^F_1_), but it has simultaneously increased the synthetic difficulties. Here, in this simpler design of **EuL2**–**7**, we show that comparable g_lum_ values (Table 2) are possible even by reducing the number of chiral centers to only 4 chiral centers by incorporating a more rigid chromophore. To obtain a more rigid chromophore, the phosphate group was modified to a carboxyl group and an increased g_lum_ values was achieved as expected.^[11]^ From our CPL studies, the largest |g_lum_| value among **EuL2**–**7**, 0.240, was observed in the ^5^D_0_ → ^7^F_1_ transition of **EuL5**. To the best of our knowledge, this is one of the highest g_lum_ values observed in such classes of Eu^III^ macrocyclic complexes. We hypothesis that the asymmetric nature of the DO3A structure with a chromophore in these Eu^III^ complexes may also assist in generating such high g_lum_ values as the non‐symmetric structure deviates the twist angles of the SAP and TSAP geometries from 40° and 29° to around ±22.5°, which is similar to previous values for a maximal g_lum_ reported by Bruce et al.^[10]^

**Table 2:** g_lum_ values of **EuL2**–**7** in 0.1 M HEPES, pH 7.3.

Transition	^5^D_0_ → ^7^F_1_	^5^D_0_ → ^7^F_2_
**EuL2**	−0.193	0.052
**(SAP)EuL3**	−0.222	0.059
**(*R*)(SAP)EuL3**	0.221	−0.058
**(TSAP)EuL3**	0.232	−0.052
**EuL4**	−0.202	0.049
**EuL5**	−0.240	0.058
**EuL6**	−0.212	0.071
**(*R*)EuL7**	0.215	−0.073

**Figure 2:**
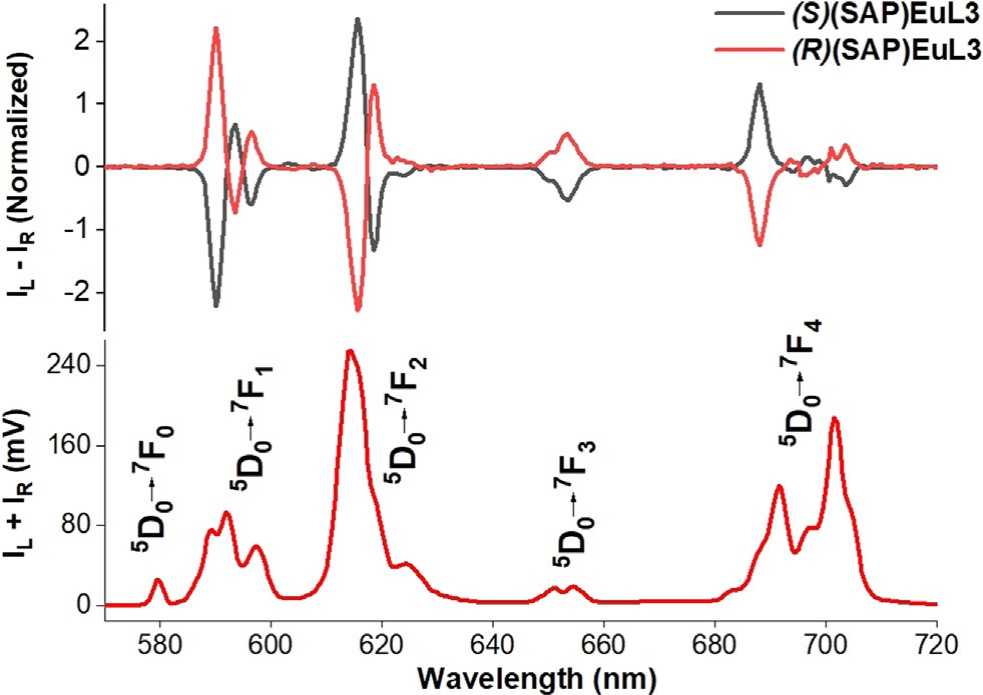
CPL spectra (upper curves) and total luminescence (lower curves) of **(SAP)EuL3** isomers in 0.1 M HEPES, pH 7.3, *λ*_exc_=340 nm.

Upon examination of the **(*S*)(SAP)EuL3** and **(*R*)(SAP)EuL3** which are of opposite handedness, these exhibited mirror images in the CPL spectra with opposite signals and g_lum_ (Figure 2). This reflects the results as expected from the isomeric nature from these two enantiomers.

**Figure 3:**
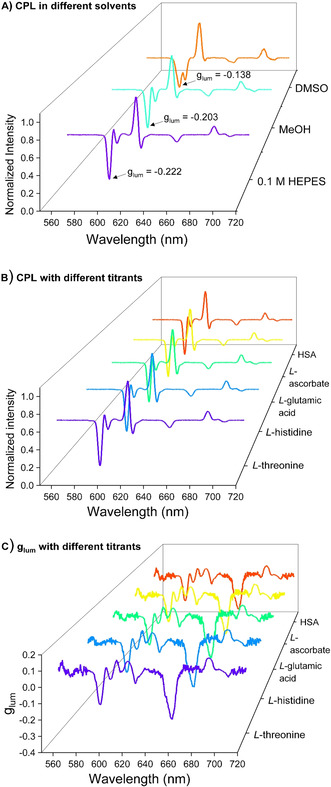
A) CPL spectra of **(SAP)EuL3** in 0.1 M HEPES (purple), MeOH (cyan) and DMSO (red), *λ*_ex_=340 nm. B) CPL spectra & C) g_lum_ spectra of **(SAP)EuL3** titrated with L‐threonine (purple), L‐histidine (blue), L‐glutamic acid (cyan), L‐ascorbate (yellow) and HSA (red) in 0.1 M HEPES, pH 7.3.

Solvent effects also exist in the CPL measurements. CPL measurements for **(SAP)EuL3** were conducted in different solvents: 0.1 M HEPES, MeOH and DMSO. In HEPES and MeOH, the CPL spectra of **(SAP)EuL3** displayed similar spectral shapes. There were, however, some differences observed in DMSO, where more spectral splitting were observed. For example, the peak of ^5^D_0_ → ^7^F_1_ transition in **(SAP)EuL3** splits into 2 in DMSO (Figure 3 A). The g_lum_ values of **(SAP)EuL3** in DMSO, MeOH and 0.1 M HEPES buffer were calculated as −0.138, −0.203 and −0.222 respectively. Interestingly, the g_lum_ values enhanced with an increase in solvent polarity. Comparing the least polar solvent (DMSO with relative polarity=0.444) to the most polar solvent (0.1 M HEPES with relative polarity=1),^[49]^ the g_lum_ value of **(SAP)EuL3** increased around 61 % (|0.138| to |0.222|). One of the possible explanations is the Pfeiffer effect between **(SAP)EuL3** and the solvent molecules, in which the solvation sheath created around the complex generates a second source of chirality affecting the g_lum_ value.^[46]^

The effects of the steric hindrance from the chiral substituents were also examined in this study. The asymmetry ratio, *R*, is calculated to reveal the geometric difference in **EuL1**–**7**, according to the equation: I(^5^D_0_ → ^7^F_2_)/I (^5^D_0_ → ^7^F_1_), which is the ratio between the integrated intensity of the peaks Δ*J*=2 and Δ*J*=1.^[20]^ The luminescent intensity of ^5^D_0_ → ^7^F_1_ transition, which is magnetic dipole‐allowed, but spin and orbit forbidden, can act as a reference as it is relatively independent of site symmetry and coordination environment of Eu^III^ ion, whereas the ^5^D_0_ → ^7^F_2_ transition, the forced electric dipole transition, is hypersensitive to the environment and the coordination symmetry.^[31]^ The *R* value thus can give an insight to whether the substituents affected the local symmetry of the Eu^III^ center. When the bulkiness of R groups was increased from methyl to isopropyl, similar *R* ratios were obtained, implying that the R groups had minimal effect on the local symmetries of the Eu^III^ centers of **EuL1**–**7**. Examination of the quantum yields also shows no apparent influences from the peripheral chiral groups. However, analysis of the g_lum_ values shows an uphill trend which can be correlated to the increased steric bulkiness of the chiral groups. This is important as it shows the sensitivity of CPL, as even peripheral or distant structural deviations can have an impact on the CPL but not the PL properties.

Altering the inner coordination sphere of Eu^III^ ion will also affect the CPL properties. However, in our nine coordinated complexes, **EuL1**–**7** is expected to have minimal changes as the Eu^III^ ion's coordination site within these complexes are fully saturated, shielding the cation from the surrounding environment. To confirm this hypothesis, **(SAP)EuL3** was titrated in a pH ranged from 4–9 with various small molecules, L‐glutamic acid, L‐histidine, L‐threonine, sodium L‐ascorbate and human serum albumin (HSA) in aqueous media and the CPL spectra were compared. From Figures 3 B & C, there was no change among these CPL spectra; moreover, the g_lum_ values remained the same. These indicated the coordination of **(SAP)EuL3** did not changed during different titrations. Stable CPL signals and g_lum_ values were obtained even when subjected to different environments.

This was further supported by examining **EuL1**–**6** under a “physiological environment” mimicked by an anion cocktail (0.9 mM HPO_4_^2−^, 100 mM chloride, 2.3 mM lactate, 0.13 mM citrate and 15 mM HCO_3_^−^),^[50]^ and also at various pH conditions (Figures S16–S29). All these europium complexes showed no significant changes in luminescence intensity or in spectral splitting upon addition of the anion cocktail. The pH titrations also showed no changes in the luminescence from the range of pH 5–10. Hence, it can be concluded that no apparent decomplexation occured even in extreme pH ranges. These Eu^III^ complexes show a high tolerance to the changes of pH under physiological conditions.

**Figure 4:**
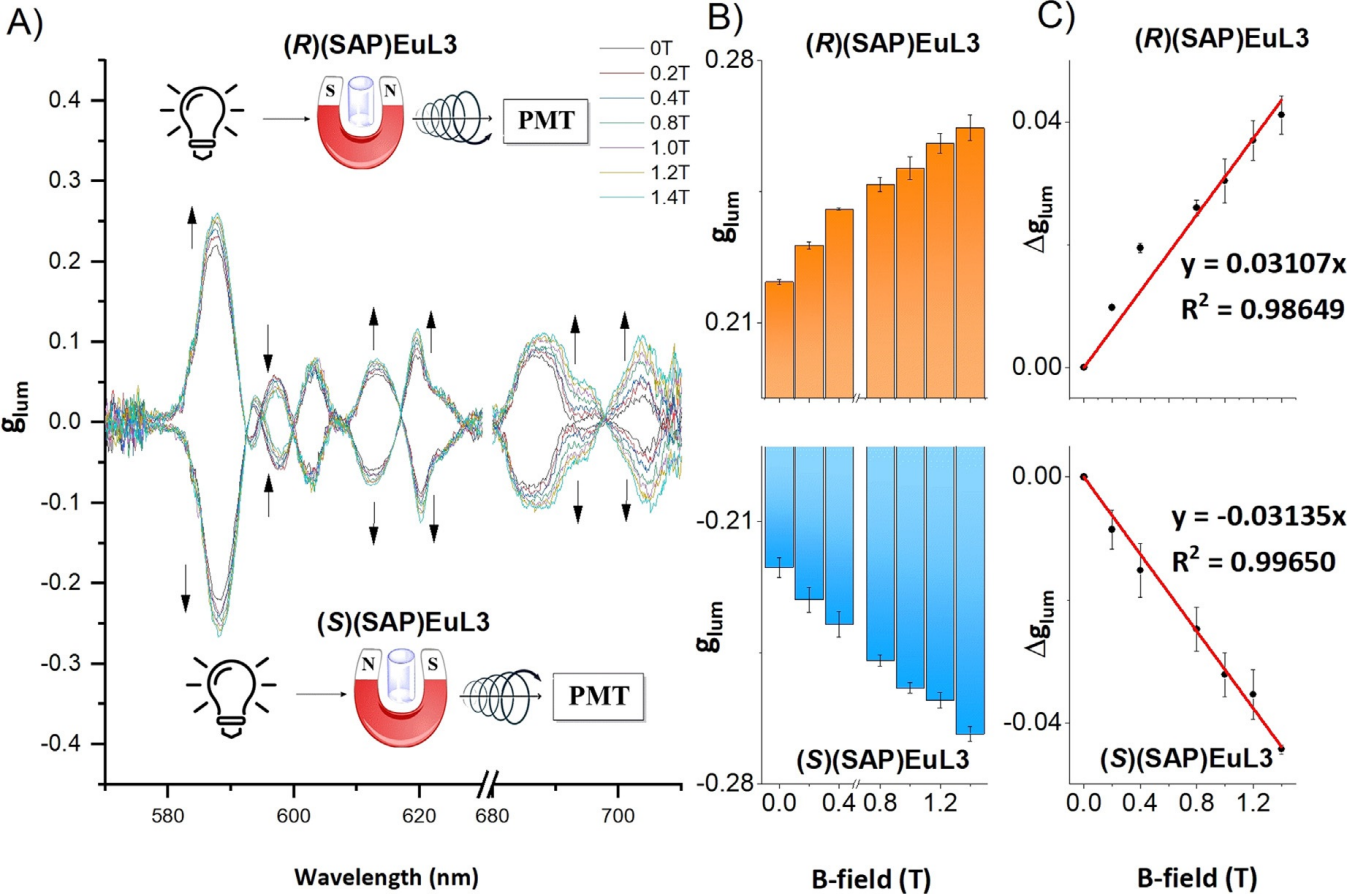
A) Magnetic field dependent g_lum_ spectra of **(*R*)&(*S*)(SAP)EuL3** in 0.1 M HEPES buffer, pH 7.3, excited at 350 nm at room temperature, arrows indicate the trends of g_lum_ changes, inserted diagrams show the direction of applied magnetic field; B) g_lum_ and C) Δg_lum_ as a function of magnetic field strength at ^5^D_0_ → ^7^F_1_ transition.

Based on the excellent quantum yields and high g_lum_ values, we further studied the magnetic responses of our complexes, **EuL2**–**7**. Although **EuL2**–**7** have intrinsic CPL signals, we hypothesised that the CPL or g_lum_ would be influenced under magnetic fields. Hence, these Eu^III^ complexes were placed in static magnetic fields to test for MCPL responses. In MCPL measurements, magnets from 0 T to 1.4 T were applied to **EuL2**–**6** in a specific direction: S to N for complexes in *S* configuration, N to S for **(*R*)(SAP)EuL3**. From the g_lum_ spectra (Figure 4), the Δg_lum_ values displayed a linear relationship with the applied magnetic field strengths. The |g_lum_| obtained from the magnetic dipole allowed ^5^D_0_ → ^7^F_1_ transition increased with increasing applied magnetic field strength and the |g_lum_| value at this transition for **(SAP)EuL3** was enhanced by 20 % from 0.222 to 0.267 from 0 T to 1.4 T at room temperature.

## Conclusion

In summary, a series of nine‐coordinated chiral macrocyclic Eu^III^ complexes were synthesized by introducing chiral substituents onto the macrocyclic backbone. A highly rigid and bioconjugatable chromophore was designed to optimise the luminescence properties. With a fully coordinated inner sphere, **EuL2**–**7** shows high stability and excellent luminescent quantum yields and g_lum_ values under various biological conditions, making these Eu^III^ complexes ideal as chiroptical probes and sensors Among these complexes, the highest quantum yield, 31.3 %, was determined in **(*R*)EuL7** and the largest |g_lum_| value of 0.240 was obtained for **EuL5**. More importantly, to the best of our knowledge, this is amongst the first series of Eu^III^ chiral DO3A derivatives to show enhanced MCPL response, with 20 % g_lum_ enhancement (from 0 T to 1.4 T), which we believe can be further optimised by higher magnetic field strengths to provide an alternative way to increase the image contrast in chiroptics. We demonstrated that these highly biocompatible complexes, **EuL2**–**7**, offers insights to the design criteria of CPL probes and simultaneously expands the choices of CPL materials used in biological studies with the additional value of MCPL.

**Please note**: Two authors (L.E.M. and R.P.) have been added to this manuscript after its appearance as Accepted Article. The Executive Committee

## Conflict of interest

The authors declare no conflict of interest.

## Supporting information

As a service to our authors and readers, this journal provides supporting information supplied by the authors. Such materials are peer reviewed and may be re‐organized for online delivery, but are not copy‐edited or typeset. Technical support issues arising from supporting information (other than missing files) should be addressed to the authors.

Supplementary
