# Supplementary material for: Unusual Magnetic Field Responsive Circularly Polarized Luminescence Probes with Highly Emissive Chiral Europium(III) Complexes
Source: Angew Chem Int Ed Engl. 2020 Nov 3;60(2):1004–10. doi: 10.1002/anie.202012133 (PMC7821146; doi:10.1002/anie.202012133)
Supplement: Supplementary file 1 — Supplementary [file ANIE-60-1004-s001.pdf]

Angewandte Chemie
Eine Zeitschrift der Gesellschaft Deutscher Chemiker

Supporting Information

Unusual Magnetic Field Responsive Circularly Polarized Luminescence Probes with Highly Emissive Chiral Europium(III) Complexes

*Junhui Zhang, Lixiong Dai, Alexandra M. Webster, Wesley Ting Kwok Chan, Lewis E. Mackenzie, Robert Pal, Steven L. Cobb, and Ga-Lai Law**

anie_202012133_sm_miscellaneous_information.pdf

Author Contributions

G.-L.L. conceived and supervised the project. L.D. performed the synthesis of the compounds. J.Z. performed all the magnetic CPL studies and CPL studies. W. T.-K.C. collected and solved the crystal structure. L.E.M. and R.P. performed some of the initial CPL experiments. A.M.W. and S.L.C. performed the peptoid work. J.Z. and G.-L.L. co-wrote the manuscript.

Content

1. General Method and Materials ***6***

2. Experimental section ***8***

2.1 Synthesis of chromophore **8**

Scheme S1. Synthesis of the chromophore compound, 6. 8

2.2 Synthesis of chiral methyl & ethyl cyclens **10**

Scheme S2. Synthesis of chiral n-propyl cyclen, A4. 10

Scheme S3. Synthesis of chiral iso-propyl cyclen, A5. 11

2.3 Synthesis of chiral ethan-1-ol cyclen **12**

Scheme S4. Synthesis of chiral ethan-1-ol cyclen, A6. 12

2.4 Synthesis of EuL1 .... **13**

Scheme S5. Synthesis of EuL1. 13

2.5 Synthesis of EuL2 .... **14**

Scheme S6. Synthesis of EuL2. 14

2.6 Synthesis of (SAP)EuL3 and (TSAP)EuL3 .... **15**

Scheme S7. Synthesis of (SAP)EuL3 and (TSAP)EuL3. 15

2.7 Synthesis of (*R*)(SAP)EuL3 .... **16**

Scheme S8. Synthesis of (*R*)(SAP)EuL3. 16

2.8 Synthesis of EuL4 .... **17**

Scheme S9. Synthesis of EuL4. 17

2.9 Synthesis of EuL5 .... **18**

Scheme S10. Synthesis of EuL5. 18

2.10 Synthesis of EuL6 .... **19**

Scheme S11. Synthesis of EuL6. 19

2.11. Synthesis of peptoid **19**

2.12. Synthesis of EuL7 .... **20**

Scheme S12. Synthesis of EuL7. 20

3. Photophysical measurements ***21***

3.1 Absorption, excitation and emission of EuL1-7 .... **21**

Figure S1. Absorption, excitation and emission spectra of EuL1 21

Figure S2. Absorption, excitation and emission spectra of EuL2. 21

Figure S3. Absorption, excitation and emission spectra of (SAP)EuL3 22

Figure S4. Absorption, excitation and emission spectra of (TSAP)EuL3 22

Figure S5. Absorption, excitation and emission spectra of (*R*)(SAP)EuL3 23

Figure S6. Absorption, excitation and emission spectra of EuL4 23

Figure S7. Absorption, excitation and emission spectra of EuL5 24

Figure S8. Absorption, excitation and emission spectra of EuL6 24

Figure S9 Absorption, excitation and emission spectra of (*R*)EuL7 25

Figure S10. Emission spectra of Eu(III) chiral ethyl DOTA in SAP and TSAP geometries 25

3.2 Low temperature measurement of GdL3A 26

Figure S11. Emission spectrum of (SAP)GdL3 at room temperature 26

Figure S12. Excited state decay curve of (SAP)GdL3 at 459 nm at room temperature 26

Figure S13. Emission spectrum of (SAP)GdL3 at 77 K 27

Figure S14. Excited state decay curve of (SAP)GdL3 at 404 nm at 77 K 27

Figure S15. Jablonski diagram of (SAP)GdL3. 28

3.3 pH titration 28

Figure S16. Emission spectra of EuL1 in different pH 28

Figure S17. Emission spectra of EuL2 in different pH 29

Figure S18. Emission spectra of (SAP)EuL3 in different pH 29

Figure S19. Emission spectra of (TSAP)EuL3 in different pH 30

Figure S20. Emission spectra of EuL4 in different pH 30

Figure S21. Emission spectra of EuL5 in different pH 31

Figure S22. Emission spectra of EuL6 in different pH 31

3.4 Anion titration 32

Figure S23. Emission spectra of EuL1 in anion titration. 32

Figure S24. Emission spectra of EuL2 in anion titration. 32

Figure S25. Emission spectra of (SAP)EuL3 in anion titration. 33

Figure S26. Emission spectra of (TSAP)EuL3 in anion titration. 33

Figure S27. Emission spectra of EuL4 in anion titration. 34

Figure S28. Emission spectra of EuL5 in anion titration. 34

Figure S29. Emission spectra of EuL6 in anion titration. 35

3.5 CD spectrum 35

Figure S30. CD spectrum of (SAP)EuL3 36

3.6 CPL spectra 36

Figure S31. CPL and g_{lum} spectra of EuL2 36

Figure S32. CPL and g_{lum} spectra of (SAP)EuL3 36

Figure S33. CPL and g_{lum} spectra of (*R*)(SAP)EuL3 37

Figure S34. CPL and g_{lum} spectra of (TSAP)EuL3 37

Figure S35. CPL and g_{lum} spectra of EuL4. 38

Figure S36. CPL and g_{lum} spectra of EuL5 38

Figure S37. CPL and g_{lum} spectra of EuL6 39
Figure S38. CPL and g_{lum} spectra of (*R*)EuL7. 39
Figure S39. g_{lum} spectra of (SAP)EuL3 in different solvents. 40
Figure S40. g_{lum} spectra of (SAP)EuL3 at different pH 41
Figure S41. *L*-Histidine titration of (SAP)EuL3 42
Figure S42. *L*-Glutamic acid titration of (SAP)EuL3 42
Figure S43. *L*-Threonine titration of (SAP)EuL3 43
Figure S44. HSA titration of (SAP)EuL3 43
Figure S45. Sodium *L*-Ascorbate titration of (SAP)EuL3 44
Figure S46. Sodium *L*-Ascorbate titration of (*R*)(SAP)EuL3 44

3.6 MCPL 45

Figure S47. MCPL study of EuL2 45
Figure S48. MCPL study of EuL4 45
Figure S49. MCPL study of EuL5 46
Figure S50. MCPL study of EuL6 46

4. Mass Spectra 47

Figure S51. Mass spectrum of EuL1 47
Figure S52. Mass spectrum of EuL2 47
Figure S53. Mass spectrum of (SAP)EuL3 48
Figure S54. Mass spectrum of (TSAP)EuL3. 48
Figure S55. Mass spectrum of (*R*)(SAP)EuL3 49
Figure S56. Mass spectrum of EuL4 49
Figure S57. Mass spectrum of EuL5 50
Figure S58. Mass spectrum of EuL6. 50
Figure S59. Mass spectrum of EuL7 51

5. HPLC Traces 52

Figure S60. HPLC trace of L2 52
Figure S61. HPLC trace of EuL2 (before purification) 52
Figure S62. HPLC trace of EuL2 (after purification) 52
Figure S63. HPLC trace of EuL3 (before purification) 52
Figure S64. HPLC trace of (SAP)EuL3 (the 1st isomer) 53
Figure S65. HPLC trace of (TSAP)EuL3 (the 2nd isomer) 53
Figure S66. HPLC trace of (*R*)(SAP)EuL3 53
Figure S67. HPLC trace of EuL4 53
Figure S68. HPLC trace of EuL5 54
Figure S69. HPLC trace of EuL6. 54
Figure S70. HPLC trace of EuL7 (UV 350 nm and fluorescence 615 nm) 54
Figure S71. HPLC trace of pure ligand L3, (SAP)EuL3 and (TSAP)EuL3 dissolved in water 55

Figure S72. HPLC trace of (SAP)EuL3 dissolved in 95% TFA. 56

6. Single Crystal X-ray Diffraction 57

Figure S73. Structure of Compound 13. 57

Figure S74. Crystal structures of Compound 13 57

7. NMR Spectra 58

8. References 85

1. General Method and Materials

All solvents and chemicals were purchased from commercial sources and directly used for synthesis without further purification. Quinine sulphate and sulfuric acid (0.1 M) used in photophysical measurements were purchased from Sigma-Aldrich. HEPES buffer (1 M) and potassium hydroxide (1 M) were purchased from Fisher Scientific and 1 M HEPES buffer was further diluted to 0.1 M using Milli-Q water (18.2MΩ at 25°C). Hydrochloric acid (1 M) and sodium hydroxide solution (1 M) were purchased from J.T.Baker. Deuterated solvents were purchased from Cambridge Isotope Laboratories Inc. ^{1}H, ^{13}C NMR spectra were recorded on a Bruker Ultrashield 400 Plus NMR spectrometer (at 400 MHz, 100 MHz respectively) or Bruker Ultrashield 600 Plus NMR spectrometer (at 600 MHz, 150 MHz respectively). Solvent residual peaks were used as references for calibration of NMR shifts. Mass spectra were obtained either on a Micromass Q-TOF 2 mass spectrometer or on an Agilent Technology 6540 UHD Accurate-Mass Q-TOF LC/MS system or on a Bruker UltrafleXtreme Matrix Assisted Laser Ionization (MALDI) Mass Spectrometer. High performance liquid chromatography (HPLC) were conducted on Waters 1525 series apparatus with PDA detector for analytical use and on Waters 2535 series apparatus with PDA detector and Fraction Collector III for reverse-phase semi-preparative purification. (HPLC methods) UV-vis absorption spectra of complexes were measured with an HP UV–8453 spectrophotometer (Santa Clara, CA, USA). Steady-state room temperature photoluminescence measurements were performed with an Edinburgh Instrument (Livingston, UK) FLSP920 spectrophotometer equipped with a Xe900 continuous xenon lamp, mF920 microsecond flash lamp and a single photon counting photomultiplier tube. Spectra were corrected with the bundled F900 software. Low temperature (77 K) measurements were measured on FLSP920 using an EPR dewar from Edinburgh Instruments, samples were dissolved in 1: 1 water/glycerol mixture. Anion and pH titration were performed with PTI QuantaMaster 500 equipped with a 75W Xenon lamp and PMT928 detector. CPL measurements were recorded on a JASCO CPL-300 Circularly Polarized Luminescence Spectrophotometer equipped with ozone-free 150W Xe arc lamp and PMT detector. Bundled magnets 0.2 T, 0.4 T, 0.8 T, 1.0 T, 1.2 T and 1.4 T were used for magnetic CPL measurements. CPL spectra were recorded using a 3-5 spectral average sequence in the range of 550–720 nm with 15-5nm slits, 0.1 nm spectral intervals and 0.5 s integration time. g_{lum} values were calculated using bundled software. All the solution-state measurements were conducted using Type 23 quartz curettes with 10 mm path length from Starna Scientific (London, UK). All the UV-vis absorption spectra, excitation spectra, emission spectra and lifetime measurements were conducted within 0.1 UV absorbance at 350nm. Quinine sulfate in 0.1M sulfuric acid (Φ = 0.577) was used as a standard in relative quantum yield measurements. Emission spectra were recorded in the range of 400-750 nm with 1 nm spectral intervals, 0.1-0.3 s integration time and 380 nm long pass filter. Lifetime measurements were recorded base on the highest intensity emission peak of each complexes and fitted using bundled F900 software. Reversed-phase semi-preparative purification was performed on the Waters HPLC system with UV detection from 220 to 330 nm using a Waters Atlantis® T3 Prep OBD™ Column (250 × 19 mm). The method used: mobile phase A was water with 0.05% TFA, mobile phase B was acetonitrile. Gradient: starting from 90% A/10% B, the fraction of B increased to 80% over 20 mins, then re-equilibrated at 10% B for 5 mins, flow rate at 8 mL/min. The analytical HPLC was performed on the Waters HPLC system using Waters Atlantis® T3 Column (250 × 4.6 mm) with UV detection from 220 to 350 nm with two methods used. Method A:

mobile phase A was H_2O with 0.1% TFA; mobile phase B was acetonitrile. Gradient: starting from 90% A/10% B, the fraction of B increased to 64% over 15 mins, then re-equilibrated at 10% B for 5 min, flow rate at 1 mL/min. Method B: mobile phase A was H_2O with 0.05% TFA; mobile phase B was acetonitrile. Gradient: starting from 90% A/10% B, the fraction of B increased to 100% over 12 mins, then re-equilibrated at 10% B for 2 min, flow rate at 1 mL/min.

Photophysical Measurements. All the photophysical measurements are performed in triplicates. Steady-state fluorescence and time-decay measurements of **EuL1-7** were conducted in water and HEPES (0.1 M, pH 7.3). The overall quantum yields (Φ) were determined using a comparative method with the well-known standard, quinine sulfate (Φ = 0.577 in 0.1 M H_2SO_4).**[1]** The intrinsic quantum yield (Φ_{Eu}^{Eu}) and sensitisation efficiency (η_{sens}) are calculated according to the publication of Aebischer *et al*. The equations are shown below: **[2]**

$$\eta_{sens} = \frac{Q_L^{Ln}}{Q_{Ln}^{Ln}} \qquad \text{Eq. (S1)}$$

$$Q_{Ln}^{Ln} = \frac{\tau_{obs}}{\tau_{rad}} \qquad \text{Eq. (S2)}$$

$$\frac{1}{\tau_{rad}} = A_{MD,0} \cdot n^3 (\frac{I_{tot}}{I_{MD}}) \qquad \text{Eq. (S3)}$$

where Q_L^{Ln} is the overall quantum yield, which can be measured; τ_{obs} is the actual lifetime of the emitting excited state and τ_{rad} is the radiative lifetime without any non-radiative de-activation processes; $A_{MD,0}$ is a constant equal to 14.65 s^{-1}; n is the refractive index of the using solvent; I_{tot} and I_{MD} are the integrated intensities of the total $^5D_0 \rightarrow {}^7F_J$ transitions and the integrated intensity of magnetic dipole transition ($^5D_0 \rightarrow {}^7F_1$) respectively.

The number of water molecules coordinated to the first coordination sphere of Eu(III) metal center (q value) was determined according to equations published by Parker *et al.*[3] and Horrocks *et al.*[4]:

Parker's equation:

$$q = A(\tau_{O-H}^{-1} - \tau_{O-D}^{-1} - 0.25 - 0.75n_{NH} \qquad \text{Eq. (S5)}$$

where A is a constant equal to 1.2 ms^{-1} for europium; τ_{O-H} is the lifetime measured in water and τ_{O-D} is the lifetime measured in deuterated water; n_{NH} is the number of carbonyl-bound amide NH oscillators with Eu.

Horrocks' equations:

$$q = A(\tau_{H2O}^{-1} - \tau_{D2O}^{-1} - k_{XH}) \qquad \text{Eq. (S6)}$$

$$k_{XH} = \alpha + \beta n_{OH} + \gamma n_{NH} + \delta n_{O=CNH} \qquad \text{Eq. (S7)}$$

where A, α, β, γ, and δ are constants equal to 1.1, 0.31, 0.45, 0.99 and 0.075 respectively; τ_{O-H} is the lifetime measured in water and τ_{O-D} is the lifetime measured in deuterated water; n_{OH} is the number of alcoholic O-H oscillators, n_{NH} is the number of amine N-H oscillators and $n_{O=CNH}$ is the number of amide N-H oscillators in which the amide carboxylic oxygen is in the first coordination sphere of Eu(III).

Titration conditions. pH titrations were performed by adjusting the pH using 1 M NaOH and 1 M HCl solution. Anion titrations were performed by adding the stock solution of anion mixture included 45Mm of Na_2HPO_4,5M of NaCl, 115mM of sodium lactate, 6.5Mm of sodium citrate and 0.75M of $NaHCO_3$ from 0µL to 120µL into 3mL aqueous solution of Eu(III) complexes.

CPL measurements. Samples were dissolved in 0.1 M HEPES, MeOH and DMSO (HPLC grade from Sigma-Aldrich) with UV absorbance at around 0.5. Excitation wavelength at 340 nm was used. 180° sample geometry

with unpolarized excitation light, the distance between sample and detector were adjusted for maximum signal intensity.[5] In magnetic studies, bundled magnet components were placed into the sample chamber from 0.2 to 1.4 T in the direction perpendicular to the excitation light beam.

Single Crystal X-ray Diffraction. The crystal data reported in the manuscript was collected on a Bruker D8-Venture Diffractometer System with a micro-focus Mo-Kα radiation. The data was collected at room temperature. Multi-scan absorption correction was applied by SADABS program,[6] and the SAINT program utilised for the integration of the diffraction profile.[7] The structure was solved by direct method and was refined by a full-matrix least-squares treatment on F^2 using the SHELXLE programme system.[8] The crystallographic data for the structural analyses have been deposited with the Cambridge Crystallographic Data Centre, CCDC No. 1991693, and the data can be obtained free of charge via www.ccdc.cam.ac.uk/data_request/cif.

2. Experimental section

2.1 Synthesis of chromophore

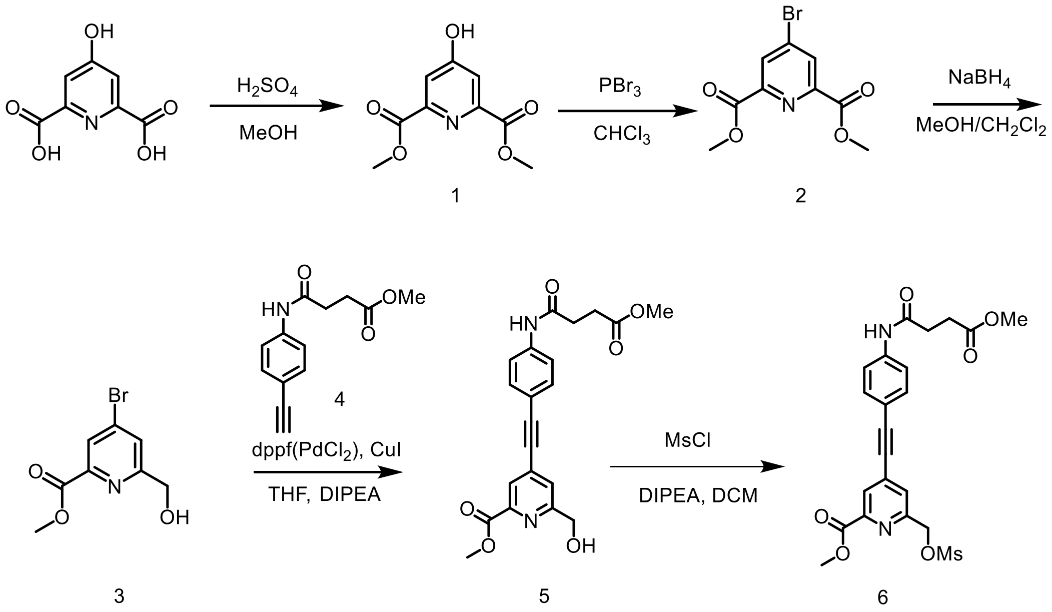

Scheme S1. Synthesis of the chromophore compound, 6.

The chromophore was synthesized according to the literature[9] with some minor modifications. Chelidamic acid (5 g, 27.3 mmol) was dissolved in 150 mL methanol and 95% H_2SO_4 (0.45 mL) was added to the solution. Pale yellow solution mixture with some solid was observed. The reaction mixture was heated to 65 °C. After 30 mins, the yellow color of solution became deeper and solid still existed. The reaction was monitored by TLC and reacted overnight. The resulting solution was concentrated under reduced pressure. Water was added to the crude product and the pH was adjusted to slightly basic by sodium bicarbonate and potassium hydroxide. Ethyl acetate (80mL x 4) was used for extraction. The organic phase was washed by saturated sodium chloride and dried by anhydrous sodium sulfate. After removing the solvent, 2.5 g yellow powder, compound **1**, was obtained with 43% yield. ^{1}H NMR (400 MHz, DMSO) δ 7.58 (s, 2H), 3.89 (d, J = 11.2 Hz, 6H). ^{13}C NMR (100 MHz, DMSO) δ 166.55, 165.29, 149.74, 115.77, 53.03. m/z (ESI-MS$^+$) 212.40 ([M+H]$^+$ calculated: 212.05).

Compound **1** (2 g, 9.47 mmol) was dissolved in 40mL dry chloroform under nitrogen protection. PBr_3 (1.34 mL, 14.2 mmol) was added directly to the reaction and heated to 90 ºC. The solution turned from pale yellow to yellowish milky after heating. The reaction was monitored by TLC. After 24 hours, the resulting mixture was cooled down to 0 ºC using ice bath and methanol was added dropwise to quench the reaction. The resulting mixture was concentrated under reduced pressure. Methanol was added to the crude product and white precipitate formed. The solution was put in fridge overnight. The precipitate was filtered. The obtained product can be used without further purification. ^1H NMR (400 MHz, DMSO) δ 8.43 (s, 2H), 3.93 (s, 6H). ^{13}C NMR (100 MHz, DMSO) δ 164.02, 149.26, 134.81, 131.19, 53.44. m/z (ESI-MS$^+$) 274.27 ($[M+H]^+$ calculated: 273.96)

Compound **3** was synthesized according to the literature method.[9] Compound 2 (0.5g, 1.82mmol) was dissolved in 25mL mixture of methanol and DCM in the ration of 4:1. The solution mixture was cooled down to 0 °C using ice bath. $NaBH_4$ (0.069g, 1.82mmol) was added gradually in small portions. The reaction mixture turned from colorless to pale yellow. The reaction was stirred overnight. When the reaction was completed, the resulting mixture was concentrated under reduced pressure. The crude product was extracted by ethyl acetate (30ml x 3) and organic phase was dried by anhydrous Na_2SO_4 without further purification. ^1H NMR (400 MHz, Chloroform-*d*) δ 8.15 (s, 1H), 7.72 (s, 1H), 4.81 (s, 2H), 3.96 (s, 3H). ^{13}C NMR (100 MHz, $CDCl_3$) δ 164.45, 161.77, 148.06, 134.53, 127.26, 127.13, 64.38, 53.18. m/z (ESI-MS$^+$) 246.21 ($[M+H]^+$ calculated: 245.97)

NMM (10.3 g, 102 mmol) was added into a mixture of 4-ethynylaniline (6 g, 51 mmol) and 4-methoxy-4-oxobutanoic acid (8.8 g, 67 mmol) in THF (50 mL), then the reaction mixture was cooled to 0 – 10 °C with ice/water bath. HATU (25 g, 66 mmol) was added slowly, then after reacting for 16 h, concentrated and the residue was poured into 300 mL of water, the precipitate was filtered and washed with water, the filter cake was dried in oven, this resulted in the product **4** as a light yellow solid (9.5 g, 80% yield). ^1H NMR (400 MHz, Chloroform-*d*) δ 7.92 (s, 1H), 7.47 (d, J = 8.4 Hz, 2H), 7.41 (d, J = 8.7 Hz, 2H), 3.70 (s, 3H), 3.03 (s, 1H), 2.74 (ddd, J = 7.2, 5.8, 1.2 Hz, 2H), 2.66 (ddd, J = 7.1, 5.7, 1.2 Hz, 2H). ^{13}C NMR (100 MHz, $CDCl_3$) δ 173.74, 169.90, 138.37, 132.90, 119.32, 117.61, 83.39, 52.06, 32.09, 29.16. m/z (ESI-MS$^+$) 232.59 ($[M+H]^+$ calculated: 232.09)

The mixture of compound **4** (2 g) and the compound **3** (2.5 g) in THF (20 mL) and DIPEA (4 mL) was degassed three times. Then added dppf($PdCl_2$) (0.26 g) and CuI (120 mg), temperature was increased to 70 ºC and the mixture was reacting at this temperature for 16 h. Then concentrated and the residue was purified by column chromatography (silica) with $CHCl_3$ and ethanol (50:1 to 20:1), this resulted in the product as a light yellow solid (compound **5**) (2.09 g, yield 65%). ^1H NMR (400 MHz, Methanol-d_4) δ 8.07 (d, J = 1.5 Hz, 1H), 7.72 (d, J = 2.6 Hz, 1H), 7.67 – 7.60 (m, 2H), 7.56 – 7.47 (m, 2H), 4.80 (s, 2H), 4.05 (s, 2H), 3.72 (s, 3H), 2.75 – 2.69 (m 4H). ^{13}C NMR (100 MHz, $CDCl_3$) δ 173.96, 170.29, 165.27, 161.12, 146.91, 139.42, 133.81, 132.83, 125.69, 125.57, 119.32, 116.69, 95.58, 85.68, 64.37, 53.00, 52.05, 31.65, 29.02. m/z (ESI-MS$^+$) 397.32 ($[M+H]^+$ calculated: 397.13).

DIPEA (2 g, 15.5 mmol) was added to the solution of compound **5** (2 g, 5 mmol) in dichloromethane (20 mL), the solution was cooled to 0 - 10°C and methanesulfonyl chloride (1.2 g, 10 mmol) was added. After reacting for 20 mins, the solution was quenched by added 10 mL of water. The organic and aqueous layers were separated and the organic layer was washed with 10 mL water and 10 mL saturated NaCl solution, dried with magnesium sulfate. The solid was filtered and the filtrate was concentrated. Compound **6** (2 g, yield 83%) was used to the next step reaction without further purification.

2.2 Synthesis of chiral methyl & ethyl cyclens

The chiral cyclens with methyl, ethyl substituents were synthesized according to our previous publications.[10-11]

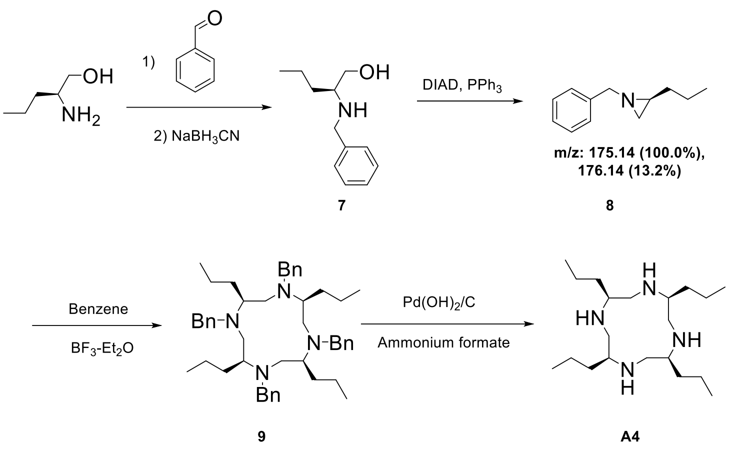

Scheme S2. Synthesis of chiral n-propyl cyclen, A4.

(*S*)-2-aminopentan-1-ol (20 g) and benzaldehyde (22.6 g) in methanol (60 mg) and dichloromethane (260 mL) was stirred at room temperature for 3 days, then concentrated and dried under vacuum for 2 days, the resulted mixture was added 1,2-dichloroethane (200 mL), cooled to 0 - 10 °C, then added $NaBH_3CN$ (15 g) slowly, after reacting for 1 hour, another 5 g of $NaBH_3CN$ was added, after another 1 hour, the resulting mixture was concentrated, added with water (200 mL), extracted with ethyl acetate (200 mL × 4), combined the organic layers and concentrated, the residue was purified by column chromatography on silica gel by ethyl acetate and petroleum ether (1: 5 to 2:1) to get the product **compound 7** (15 g, 40%) as a colorless oil. ^{1}H NMR (400 MHz, $CDCl_3$) δ 7.35 – 6.94 (m, 20H), 3.74 (d, *J* = 14.1 Hz, 4H), 3.23 (t, *J* = 9.9 Hz, 4H), 3.05 (d, *J* = 12.3 Hz, 4H), 2.96 (d, *J* = 14.1 Hz, 3H), 2.17 – 1.88 (m, 4H), 1.77 – 1.47 (m, 10H), 1.51 – 1.18 (m, 8H), 0.96 (t, *J* = 7.0 Hz, 12H). ^{13}C NMR (100 MHz, $CDCl_3$) δ 128.51, 128.21, 127.13, 63.12, 58.18, 51.13, 33.83, 19.26, 14.27. m/z (ESI-MS$^+$) 194.25 ($[M+H]^+$ calculated: 194.15).

PPh_3 (28.6 g) was added into a solution of **compound 7** (15.0 g) in THF (150 mL), then cooled the reaction mixture to 0 - 10 °C, then dropped with DIAD (22 g), the resulted mixture was reacted for 16 h at room temperature, then concentrated and added 500 mL of hexane to form precipitate, the solid was removed by filtration. The filtrate was concentrated and the residue was purified by distillation under vacuum, this resulted in the product as a colorless liquid, **compound 8** (7 g, yield 51%). ^{1}H NMR (400 MHz, $CDCl_3$) δ 7.46 – 6.99 (m, 5H), 3.88 – 3.70 (m, 2H), 3.64 (dd, *J* = 10.7, 4.0 Hz, 1H), 3.33 (dd, *J* = 10.8, 6.4 Hz, 1H), 2.67 (dt, *J* = 11.0, 5.3 Hz, 1H), 1.59 – 1.11 (m, 4H), 0.90 (t, *J* = 7.1 Hz, 3H). ^{13}C NMR (100 MHz, $CDCl_3$) δ 139.44, 128.29, 128.16, 126.95, 64.99, 39.64, 35.10, 34.04, 20.65, 13.88. m/z (ESI-MS$^+$) 176.19 ($[M+H]^+$ calculated: 176.14).

$BF_3{\cdot}Et_2O$ (0.3 mL) was added to a solution of **compound 8** (6 g) in dried benzene (60 mL), the mixture was stirred under reflux for 24 h. Then cooled and 2 mL of saturated sodium bicarbonate was added, then concentrated and the residue was purified by recrystallization in benzene and ethyl acetate, inorganic salts were washed by water. The solid was dried in oven and this resulted in the product as a white solid, **compound 9** (470 mg, 7.8%). ^{1}H NMR (400 MHz, CDCl3) δ 7.46 – 7.05 (m, 20H), 3.74 (d, *J* = 14.1 Hz, 4H), 3.23 (t, *J* = 9.9 Hz, 4H), 3.05 (d, *J* = 12.3 Hz, 4H), 2.96 (d, *J* = 14.1 Hz, 3H), 2.22 – 1.99 (m, 4H), 1.68 – 1.54 (m, 9H), 1.48 –

1.20 (m, 8H), 0.96 (t, J = 7.0 Hz, 12H). ^{13}C NMR (100 MHz, $CDCl_3$) δ 140.94, 128.46, 128.02, 126.44, 52.49, 47.27, 32.27, 19.84, 14.27. m/z (ESI-MS$^+$) 701.60 ($[M+H]^+$ calculated: 701.54)

$Pd(OH)_2$/C (200 mg) and ammonium formate (500 mg) were added into the solution of **compound 9** (470 mg) in trifluoroethanol (15 mL), after reacting at 50 °C for 16 h, a filtration was performed and the filtrate was concentrated under vacuum to get the product a light yellow solid, **compound A4** (228 mg, yield 100%). ^{1}H NMR (400 MHz, $CDCl_3$) δ 2.72 (dd, J = 12.8, 2.7 Hz, 4H), 2.55 – 2.31 (m, 8H), 1.63 – 1.06 (m, 12H), 0.91 (t, J = 7.1 Hz, 12H). ^{13}C NMR (100 MHz, $CDCl_3$) δ 53.09, 49.82, 35.15, 19.41, 14.46. m/z (ESI-MS$^+$) 341.41 ($[M+H]^+$ calculated: 341.36)

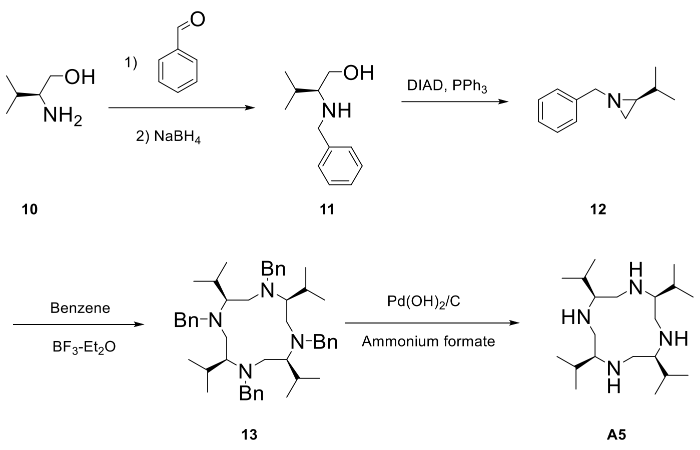

Scheme S3. Synthesis of chiral iso-propyl cyclen, A5.

(*S*)-2-amino-3-methylbutan-1-ol (**compound 10**, 20 g) and benzaldehyde (41 g) in methanol (60 mg) and dichloromethane (260 mL) were stirred at room temperature for 3 days, then concentrated and dried under vacuum for 2 days, petroleum ether (200 mg) was added, cooled to 0 - 10 °C, then stirred at this temperature for 1 h, filtered and the solid was dried in vacuum as a white solid (25 g). Then dissolved in methanol (250 mL), added $NaBH_4$ (5 g), after stirred at room temperature for 1 h, the resulting mixture was concentrated, added with water (200 mL), extracted with ethyl acetate (200 mL × 4), combined the organic layers and concentrated, the residue was purified by column chromatography on silica gel by ethyl acetate and petroleum ether (1: 5 to 2:1) to get the product **11** (18 g, 48%) as a colorless oil. ^{1}H NMR (400 MHz, $CDCl_3$) δ 7.33 (m, 5H), 3.79 (q, J = 12.8 Hz, 2H), 3.64 (dd, J = 10.6, 4.2 Hz, 1H), 3.38 (dd, J = 10.6, 7.0 Hz, 1H), 2.47 (td, J = 6.6, 4.3 Hz, 1H), 1.88 (m, 1H), 0.98 (d, J = 6.8 Hz, 3H), 0.92 (d, J = 6.9 Hz, 3H). ^{13}C NMR (101 MHz, $CDCl_3$) δ 140.41, 128.50, 128.18, 127.13, 63.89, 60.46, 51.44, 28.83, 19.57, 18.44. m/z (ESI-MS$^+$) 194.20 ($[M+H]^+$ calculated: 194.15).

PPh_3 (29 g) was added into a solution of **11** (15.0 g) in THF (150 mL), then cooled the reaction mixture to 0 - 10 °C, then dropped with DIAD (23 g), the resulted mixture was reacted for 16 h at room temperature, then concentrated and added 400 mL of hexane to form precipitate, the solid was removed by filtration. The filtrate was concentrated and the residue was purified by distillation under vacuum, this resulted in the product as a colorless liquid, **compound 12** (10 g, yield 74%). ^{1}H NMR (400 MHz, $CDCl_3$) δ 7.47 – 7.19 (m, 5H), 3.53 (d, J = 13.0 Hz, 1H), 3.24 (d, J = 13.0 Hz, 1H), 1.65 (d, J = 3.0 Hz, 1H), 1.36 (d, J = 5.9 Hz, 1H), 1.27 – 1.20 (m, 1H), 0.89 (d, J = 6.4 Hz, 1H), 0.85 (d, J = 6.2 Hz, 1H). ^{13}C NMR (101 MHz, $CDCl_3$) δ 139.47, 128.45, 128.26, 127.00, 65.30, 46.59, 33.30, 31.64, 20.53, 19.62. m/z (ESI-MS$^+$) 176.22 ($[M+H]^+$ calculated: 176.14).

$BF_3·Et_2O$ (0.3 mL) was added to a solution of **12** (6 g) in dried benzene (60 mL), the mixture was stirred under reflux for 24 h. Then cooled and 2 mL of saturated sodium bicarbonate was added, then concentrated and the residue was purified by recrystallization in benzene and ethyl acetate, inorganic salts were washed by water. The solid was dried in oven and this resulted in product as a white solid, **compound 13** (1.5 g, 25%). ^1H NMR (400 MHz, $CDCl_3$) δ 7.47 – 7.07 (m, 20H), 3.48 (d, J = 13.3 Hz, 4H), 3.29 (d, J = 13.3 Hz, 4H), 3.06 (m, 4H), 2.88 (m, 4H), 2.29 (m, 4H), 1.95 (m, 4H), 0.87 (t, J = 14.0 Hz, 12H), 0.76 (d, J = 6.5 Hz, 12H). ^{13}C NMR (400 MHz, $CDCl_3$) δ 140.66, 129.56, 127.93, 126.57, 54.68, 48.04, 27.58, 20.66. m/z (ESI-MS$^+$) 701.67 ($[M+H]^+$ calculated: 701.51).

$Pd(OH)_2/C$ (200 mg) and ammonium formate (500 mg) were added into the solution of **13** (1 g) in trifluoroethanol (15 mL), after reacting at 50 °C for 16 h, a filtration was performed and the filtrate was concentrated under vacuum to get the product a light yellow solid, **compound A5** (450 mg, yield 93%). ^1H NMR (400 MHz, $CDCl_3$) δ 2.70 – 2.59 (m, 4H), 2.45 (m, 4H), 2.34 (m, 4H), 1.94 (m, 4H), 1.81 (m, 4H), 0.92 (d, J = 6.9 Hz, 3H), 0.85 (d, J = 6.9 Hz, 3H). ^{13}C NMR (400 MHz, $CDCl_3$) δ 58.52, 45.38, 28.36, 19.98, 17.04. m/z (ESI-MS$^+$) 341.93 ($[M+H]^+$ calculated: 341.36).

2.3 Synthesis of chiral ethan-1-ol cyclen

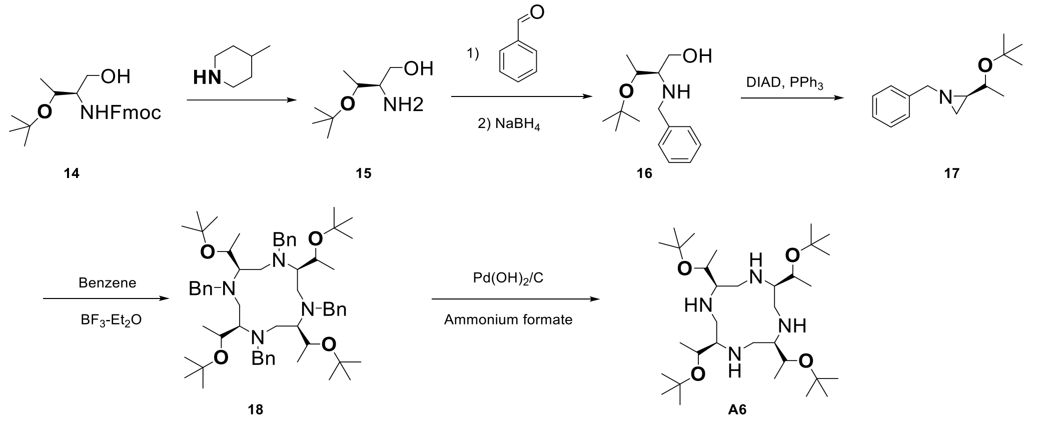

Scheme S4. Synthesis of chiral ethan-1-ol cyclen, A6.

4-methylpiperidine (10 mL) was added to **compound 14** (15 g) in DCM (100 mL), reacted at room temperature for 3 h, concentrated and added methanol (50 mL), cooled to 0 - 10 °C and filtered, the filtrate was concentrated and this resulted in the product **15** (6 g) which was used to the next step reaction directly without further purification. m/z (ESI-MS$^+$) 162.18 ($[M+H]^+$ calculated: 162.14)

Compound **15** (6 g) was dissolved in methanol (18 mL) and DCM (78 mL), then benzaldehyde (4.15 g) was added. After reacting at room temperature for 16 h, concentrated under reduced pressure and then dissolved in methanol (150 mL), $NaBH_4$ (2.9 g) was added, after reacting at room temperature for 30 min, the mixture was concentrated and purified by silica gel column chromatography with ethyl acetate and petroleum ether (1:5 to 2:1). This resulted in the product **16** (6 g, yield 64.5%) as a colorless oil. ^1H NMR (400 MHz, $CDCl_3$) δ 7.47 – 7.11 (m, 5H), 3.80 (q, J = 12.9 Hz, 2H), 3.65 (dd, J = 10.6, 4.2 Hz, 1H), 3.39 (dd, J = 10.6, 7.0 Hz, 1H), 2.48 (td, J = 6.6, 4.2 Hz, 1H), 1.88 (h, J = 6.8 Hz, 1H), 0.98 (d, J = 6.9 Hz, 3H), 0.92 (d, J = 6.8 Hz, 3H). ^{13}C NMR (100 MHz, $CDCl_3$) δ 140.47, 128.46, 127.89, 127.00, 67.96, 63.06, 60.13, 28.64, 13.47. m/z (ESI-MS$^+$) 252.24 ($[M+H]^+$ calculated: 252.19)

PPh_3 (8.1 g) was added into a soliton of **16** (5.5 g) in THF (100 mL), then cooled the reaction mixture to 0 - 10 °C, then added DIAD (5.7 g) dropwise, the resulted mixture was reacted for 16 h at room temperature, then concentrated and added 200 mL of hexane to form precipitate, the solid was removed by filtration. The filtrate was concentrated and the residue was purified by silica gel column chromatography with ethyl acetate and petroleum ether (1:20 to 1:5). This resulted in the product **17** (4 g, yield 78%) as a colorless oil. ^{1}H NMR (400 MHz, $CDCl_3$) δ 7.58 – 7.03 (m, 5H), 3.38 (d, *J* = 2.0 Hz, 2H), 3.35 – 3.23 (m, 1H), 1.74 – 1.54 (m, 2H), 1.40 (dd, *J* = 6.4, 1.9 Hz, 1H), 1.11 (s, 9H), 1.08 (d, *J* = 6.4 Hz, 3H). ^{13}C NMR (100 MHz, $CDCl_3$) δ 139.30, 128.56, 128.25, 127.02, 73.36, 69.44, 64.94, 45.38, 31.36, 28.52, 20.42. m/z (ESI-MS$^+$) 234.25 ([M+H]$^+$ calculated: 234.18)

$BF_3 \cdot Et_2O$ (0.093 mL) was added to a solution of **17** (2.7 g) in dried benzene (27 mL), the mixture was stirred under reflux for 24 h. Cooled and 2 mL of saturated sodium bicarbonate was added, then concentrated and the residue was purified by recrystallization in benzene. The solid was dried in oven and this resulted in product as a white solid **18** (400 mg, 14.8%). ^{1}H NMR (400 MHz, $CDCl_3$) δ 7.81 – 6.68 (m, 20H), 4.62 (d, *J* = 12.6 Hz, 4H), 3.89 (dd, *J* = 6.2, 1.8 Hz, 4H), 3.01 (d, *J* = 12.6 Hz, 4H), 2.88 – 2.41 (m, 12H), 1.18 (d, *J* = 6.2 Hz, 12H), 0.81 (s, 36H). ^{13}C NMR (100 MHz, $CDCl_3$) δ 141.84, 130.48, 127.77, 126.36, 72.61, 68.04, 58.19, 57.01, 45.36, 28.86, 22.13. m/z (ESI-MS$^+$) 933.78 ([M+H]$^+$ calculated: 933.71)

$Pd(OH)_2/C$ (200 mg) and ammonium formate (500 mg) was added into the solution of **18** (400 mg) in trifluoroethanol (15 mL). After reacting at 50 °C for 16 h, a filtration was performed and the filtrate was concentrated under vacuum to get the product a white solid **A6** (232 mg, yield 95%). ^{1}H NMR (400 MHz, $CDCl_3$) δ 4.01 – 3.86 (m, 4H), 2.93 (dd, *J* = 13.2, 2.6 Hz, 4H), 2.65 (dt, *J* = 11.3, 3.3 Hz, 4H), 2.40 (dd, *J* = 13.3, 10.7 Hz, 4H), 1.19 (s, 36H), 1.03 (d, *J* = 6.4 Hz, 9H). ^{13}C NMR (100 MHz, $CDCl_3$) δ 65.50, 56.12, 43.65, 28.44, 15.89. m/z (ESI-MS$^+$) 573.60 ([M+H]$^+$ calculated: 573.52)

2.4 Synthesis of EuL1

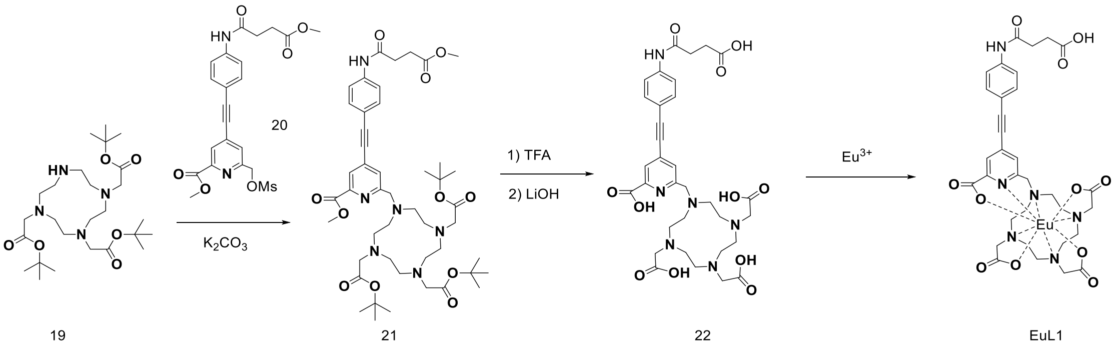

Scheme S5. Synthesis of EuL1.

DIPEA (2 g) was added to a solution of **compound 5** (2 g) in dichloromethane (20 mL), the solution was cooled to 0 - 10 °C and added methanesulfonyl chloride (1.2 g), after reacting for 30 mins, the solution was quenched by added 10 mL of water, separated the organic and aqueous layers, the organic layer was washed with 10 mL water and 10 mL sat. NaCl solution, dried with magnesium sulfate, filtered and the filtrate was concentrated. The product of **20** (2 g, yield 83%) was used to the next step reaction without any further purification.

Compound **19** was synthesized according to our previous publication.[10] Compound **19** (200 mg) was dissolved in acetonitrile (6 mL), then K_2CO_3 (161 mg) and compound **20** (240 mg) were added into the reaction mixture.

After reacting at room temperature for 12 hrs. The solution was filtered and the filtrate was concentrated under reduced pressure to give a brown oil. This crude product was dissolved into 0.3% HCl (60 mL), then extracted with ethyl acetate (3 × 20 mL), the aqueous solution was then adjusted pH to 8.0 by saturated NaHCO3 and extracted with CH_2Cl_2 (2 × 30 mL), combined the CH_2Cl_2 layers and washed with saturated NaCl (20 mL), dried over MgSO4, filtered and the solvent was removed under reduced pressure. This resulted in the product **21** (267 mg, yield 77%) as a light yellow foamy solid. ^{1}H NMR (400 MHz, $CDCl_3$) δ 7.95 – 7.68 (m, 2H), 7.40 (d, *J* = 8.3 Hz, 2H), 7.15 (d, *J* = 7.9 Hz, 2H), 3.68 (d, *J* = 4.1 Hz, 3H), 3.46 – 1.80 (m, 31H), 1.23 (s, 9H), 1.09 (s, 18H). m/z (ESI-MS$^+$) 893.53 ([M+H]$^+$ calculated: 893.49).

Compound **21** (200 mg) was firstly deprotected with trifluoroacetic acid (2 mL) at room temperature for one night, then concentrated to get the half deprotected product. It was further deprotected with lithium hydroxide (100 mg) in methanol (5 mL) and water (2 mL) at room temperature for another 16 h, the product was purified by semi-preparative HPLC and lyophilized to get the product as a yellow solid, compound **22** (80 mg, yield 52%). ^{1}H NMR (400 MHz, D_2O) δ 7.62 (s, 1H), 7.49 (s, 1H), 7.17 (s, 4H), 4.41 – 2.64 (m, 24H), 2.52 (s, 4H). m/z (ESI-MS$^+$) 697.35 ([M+H]$^+$ calculated: 697.28).

Compound **22** (50 mg) in water (2 mL) and europium chloride hexahydrate (28 mg) was mixed, after refluxing for 16 h, the temperature was cooled down and the complex was purified by semi-preparative HPLC and lyophilized to get the product (**EuL1**) as a yellow solid (45 mg, yield 75%). m/z (ESI-MS$^-$) 845.1675 ([M]$^-$ calculated: 845.1654).

2.5 Synthesis of EuL2

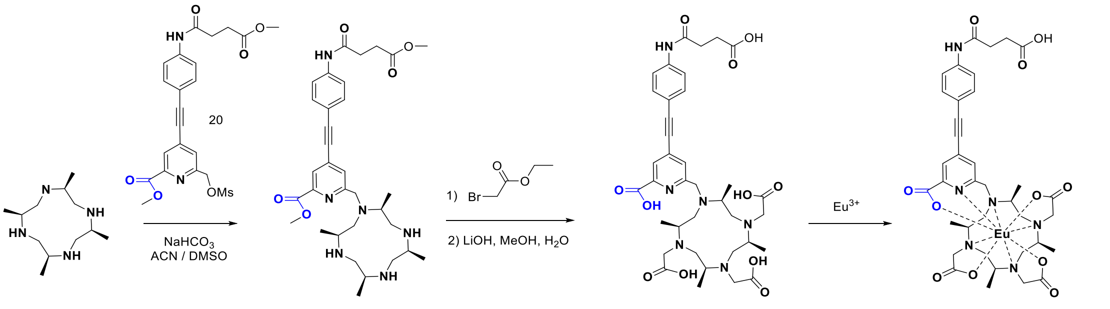

Scheme S6. Synthesis of EuL2.

Compound **A2** (500 mg) was dissolved in acetonitrile (40 mL), $NaHCO_3$ (183 mg) was added, followed by adding compound **20** (500 mg) in DMSO (2 mL) and acetonitrile (8 mL) dropwise into the reaction mixture at 50 °C slowly (10 h). Then, reacted for another 8 h. When the solution was cooled down, filtered the solid and the filtrate was concentrated under reduced pressure to give brown oil. This crude product was purified by semi-preparative HPLC and concentrated to get the product as a light yellow oil, **B2** (220 mg, yield 17%). ^{1}H NMR (400 MHz, CD_3OD) δ 7.68 (s, 1H), 7.42 – 6.97 (m, 5H), 3.63 (s, 3H), 3.29 (s, 3H), 3.20 – 2.20 (m, 18H), 0.85 – 0.84 (m, 12H). ^{13}C NMR (100 MHz, CD_3OD) δ 173.49, 171.39, 165.09, 159.32, 147.42, 140.20, 134.11, 132.57, 127.81, 125.79, 119.38, 116.32, 95.72, 85.03, 55.85, 52.39, 52.03, 51.01, 50.60, 49.71, 45.19, 39.26, 30.95, 28.47, 16.29, 12.61, 12.00, 9.12. m/z (ESI-MS$^+$) 607.40 ([M+H]$^+$ calculated: 607.35).

K_2CO_3 (500 mg) and ethyl 2-bromoacetate (365 mg) were added to compound **B2** (220 mg) in acetonitrile (5 mL), the mixture was stirred at 50 °C for 16 h, then cooled down and filtered. The filtrate was concentrated and

the residue was dissolved in methanol (3 mL), followed by adding LiOH (250 mg) in water (3 mL). It was stirred at room temperature for 12 h, pH was adjusted to about 7 and the mixture was purified by semi-preparative HPLC and concentrated to get the product as a yellow solid, **L2** (100 mg, 37%). ^{1}H NMR (400 MHz, CD_3OD) δ 7.83 – 6.82 (m, 6H), 4.21 – 2.15 (m, 24H), 1.08 – 0.54 (m, 12H). ^{13}C NMR (100 MHz, CD_3OD) δ 174.96, 174.05, 173.35, 171.70, 167.19, 165.57, 153.10, 147.95, 140.14, 134.28, 132.62, 132.52, 128.64, 126.13, 119.34, 95.57, 85.13, 59.32, 58.12, 54.28, 53.62, 53.45, 52.26, 50.78, 50.38, 49.73, 48.82, 31.03, 28.53, 9.98, 9.65, 8.97, 8.87. m/z (ESI-MS$^+$) 753.41 ($[M+H]^+$ calculated: 753.34).

Ligand **L2** (50 mg) in water (2 mL) and europium chloride hexahydrate (26 mg) was mixed together, adjusted the pH to 7.0. After refluxing for 16 h, the temperature was cooled down and the complex was purified by semi-preparative HPLC and lyophilized to get the product as a yellow solid, **EuL2** (40 mg, yield 68%). m/z (ESI-MS$^-$) 901.2280 ($[M]^-$ calculated: 901.2265). m/z (ESI-MS$^-$) 901.2280 ($[M]^-$ calculated: 901.2265).

2.6 Synthesis of (SAP)EuL3 and (TSAP)EuL3

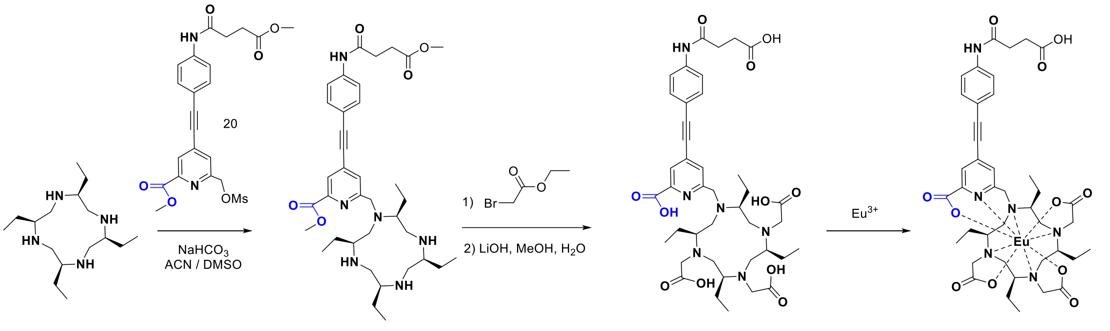

Scheme S7. Synthesis of (SAP)EuL3 and (TSAP)EuL3.

Compound **(*S*)A3** (440 mg) was dissolved in acetonitrile (40 mL), $NaHCO_3$ (130 mg) was added, followed by adding compound **20** (300 mg) in DMSO (2 mL) and acetonitrile (20 mL) dropwise into the reaction mixture at 50 °C slowly (5 h). Then, reacted for another 8 h, the solution was cooled down, filtered and the filtrate was concentrated under reduced pressure to give a brown oil. This crude product was purified by semi-preparative HPLC and concentrated to get the product as a light yellow oil **(*S*)B3** (170 mg, yield 17%). ^{1}H NMR (400 MHz, CD_3OD) δ 7.47 (d, J = 10.8 Hz, 1H), 7.06 (d, J = 8.3 Hz, 2H), 6.98 (d, J = 4.8 Hz, 1H), 6.94 (d, J = 8.4 Hz, 2H), 3.76 – 3.18 (m, 4H), 3.09 (s, 3H), 3.02 – 1.79 (m, 17H), 1.55 – 0.69 (m, 8H), 0.52 – 0.37 (m, 12H). ^{13}C NMR (100 MHz, CD_3OD) δ 173.54, 171.46, 165.30, 159.18, 147.31, 140.13, 134.13, 132.52, 127.86, 125.85, 119.40, 116.41, 116.37, 95.70, 85.01, 84.94, 57.09, 55.89, 54.04, 53.26, 52.35, 50.97, 50.89, 49.65, 44.69, 43.81, 42.73, 30.93, 28.46, 23.02, 21.09, 20.16, 18.85, 10.91, 10.13, 9.69, 9.55, 8.59, 8.55, 8.25, 7.24, 7.21. m/z (ESI-MS$^+$) 663.52 ($[M+H]^+$ calculated: 663.42).

K_2CO_3 (354 mg) and ethyl 2-bromoacetate (255 mg) were added into compound **(*S*)B3** (170 mg) in acetonitrile (5 mL), the mixture was stirred at 50 °C for 16 h, then cooled down and filtered, concentrated under reduced pressure and the residue was dissolved in methanol (3 mL). LiOH (100 mg) in water (3 mL) was added and stirred at room temperature for 12 h. pH was adjusted to about 7 and the mixture was purified by semi-preparative HPLC and concentrated to get the product as a yellow solid **(*S*)L3** (36 mg, 17%). ^{1}H NMR (400 MHz,

D_2O) δ 7.82 (s, 1H), 7.71 -7.60 (m, 1H), 7.41 – 7.10 (m, 4H), 4.04 - 2.65 (m, 20H), 2.46 (s, 4H), 1.80 – 1.12 (m, 8H), 0.91 – 0.37 (m, 12H). m/z (ESI-MS$^+$) 809.45 ($[M+H]^+$ calculated: 809.40).

Europium chloride hexahydrate (9.3 mg) was added to the solution of ligand **(*S*)L3** (20 mg) in water (2 mL), the pH was adjusted to 7.0. After refluxing for 16 h, the reaction mixture was cooled down and the complex was purified by semi-preparative HPLC and lyophilized to get two isomers, the first peak **(SAP)EuL3** (14 mg, 60% yield). m/z (ESI-MS$^+$) 959.3080 ($[M+2H]^+$ calculated: 959.3063). The second peak **(TSAP)EuL3** (4 mg, 16%), as yellow solids. m/z (ESI-MS$^+$) 959.3073 ($[M+2H]^+$ calculated: 959.3063).

2.7 Synthesis of (*R*)(SAP)EuL3

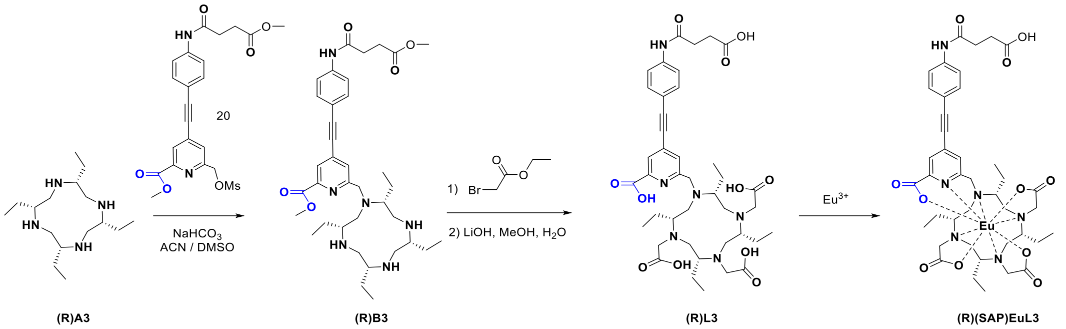

Scheme S8. Synthesis of (*R*)(SAP)EuL3.

Compound **(*R*)A3** (500 mg) was dissolved in acetonitrile (40 mL), $NaHCO_3$ (140 mg) was added, followed by adding compound **20** (340 mg) in DMSO (2 mL) and acetonitrile (8 mL) dropwise into the reaction mixture at 50 °C slowly (10 h). Then, reacted for another 8 h. When the solution was cooled down, filtered and the filtrate was concentrated under reduced pressure to give a brown oil. This crude product was purified by semi-preparative HPLC and concentrated to get the product as a light yellow oil **(*R*)B3** (220 mg, yield 19%). m/z (ESI-MS$^+$) 663.50 ($[M+H]^+$ calculated: 663.42).

K_2CO_3 (400 mg) and ethyl 2-bromoacetate (300 mg) were added to the solution of compound **(*R*)B3** (200 mg) in acetonitrile (5 mL), the mixture was stirred at 50 °C for 16 h, then cooled down and filtered, the filtrate was concentrated under reduced pressure. The residue was dissolved in methanol (3 mL), LiOH (100 mg) in water (3 mL) was added. The reaction mixture was stirred at room temperature for 12 h, pH was adjusted to about 7 and the mixture was purified by semi-preparative HPLC and concentrated to get the product as a yellow solid **(*R*)L3** (80 mg, 33%). ^{1}H NMR (400 MHz, D_2O) δ 8.09 – 7.40 (m, 6H), 4.30 – 2.60 (m, 24H), 2.07 – 1.16 (m, 8H), 1.02 – 0.64 (m, 12H). m/z (ESI-MS$^+$) 809.40 ($[M+H]^+$ calculated: 809.40).

Europium chloride hexahydrate (19 mg) was added to the solution of ligand **(*R*)L3** (40 mg) in water (3 mL), the pH was adjusted to 7.0. After refluxing for 16 h, the reaction mixture was cooled down and the complex was purified by semi-preparative HPLC and lyophilized, only one isomer was obtained with good purity (31 mg, 67% yield) as the yellow solid, **(*R*)(SAP)EuL3**. m/z (ESI-MS$^-$) 957.2920 ($[M]^-$ calculated: 957.2907).

2.8 Synthesis of EuL4

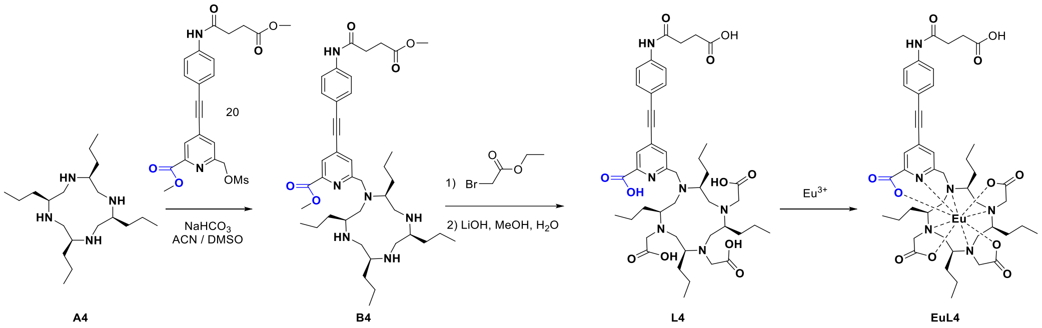

Scheme S9. Synthesis of EuL4.

Compound **A4** (228 mg) was dissolved in acetonitrile (20 mL), $NaHCO_3$ (71 mg) was added, followed by adding compound **20** (144 mg) in DMSO (1 mL) and acetonitrile (8 mL) dropwise into the reaction mixture at 50 °C slowly (10 h). Then, reacted for another 8 h. When the solution was cooled down, the solid was filtered and the filtrate was concentrated under reduced pressure to give a brown oil. This crude product was purified by semi-preparative HPLC and concentrated to get the product as a light yellow oil, **B4** (110 mg, yield 23%). ^{1}H NMR (400 MHz, D_2O+CD_3OD) δ 7.89 (s, 1H), 7.69 – 7.17 (m, 5H), 4.12 – 3.33 (m, 8H), 2.54 – 2.151 (m, 16H), 1.59 – 1.23 (m, 16H), 0.87 – 0.80 (m, 12H). ^{13}C NMR (100 MHz, CD_3OD) δ 171.85, 169.77, 163.64, 163.39, 158.06, 157.56, 145.96, 145.67, 138.52, 138.49, 132.55, 132.52, 130.84, 126.23, 124.20, 124.01, 117.69, 114.64, 94.16, 83.18, 54.37, 52.66, 52.19, 51.27, 50.73, 50.67, 49.97, 49.28, 48.17, 31.57, 29.26, 28.74, 27.75, 26.77, 26.54, 18.59, 18.07, 17.64, 17.52, 17.31, 16.89, 16.50, 15.82, 11.41, 11.20, 11.18, 11.06. m/z (ESI-MS$^+$) 719.55 ($[M+H]^+$ calculated: 719.48).

K_2CO_3 (211 mg) and ethyl 2-bromoacetate (155 mg) was added to compound **B4** (110 mg) in acetonitrile (5 mL), the mixture was stirred at 50 °C for 16 h, then cooled down and filtered the solid. The filtrate was concentrated and the residue was dissolved in methanol (3 mL). LiOH (100 mg) in water (3 mL) was added and the reaction mixture was stirred at room temperature for 12 h, pH was adjusted to about 7, the precipitate was collected by filtration, washed with water and dried in oven, resulted in the product as a yellow solid, **L4** (30 mg, 23%). ^{1}H NMR (400 MHz, CD_3OD) δ 8.17 – 7.13 (m, 6H), 4.57 – 2.68 (m, 20H), 2.54 (d, *J* = 4.1 Hz, 4H), 2.04 – 1.09 (m, 16H), 1.02 – 0.67 (m, 12H). m/z (ESI-MS$^+$) 865.57 ($[M+H]^+$ calculated: 865.46).

Europium chloride hexahydrate (5 mg) was added to ligand **L4** (10 mg) in water (2 mL), the pH was adjusted to 7.0. After refluxing for 16 h, the temperature was cooled down and the crude product was purified by semi-preparative HPLC and concentrated to get the final product as a yellow solid, **EuL4** (5 mg, yield 44%). m/z (ESI-MS$^-$) 1013.3542 ($[M]^-$ calculated: 1013.3533).

2.9 Synthesis of EuL5

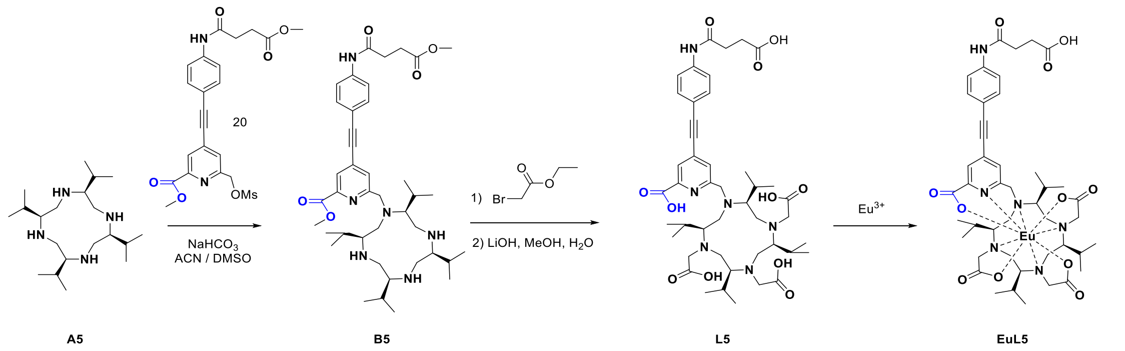

Scheme S10. Synthesis of EuL5.

Compound **A5** (240 mg) was dissolved in acetonitrile (20 mL) and $NaHCO_3$ (100 mg) was added, followed by adding compound **20** (115 mg) in DMSO (1 mL) and acetonitrile (8 mL) dropwise into the reaction mixture at 50 °C slowly (10 h). Then, reacted for another 8 h. When the solution was cooled down, filtered the solid and the filtrate was concentrated under reduced pressure to give a brown oil. This crude product was purified by semi-preparative HPLC and concentrated to get the product as a light yellow oil, **B5** (90 mg, yield 18%). ^{1}H NMR (400 MHz, $CDCl_3$) δ 8.51 – 7.07 (m, 6H), 4.87 – 3.57 (m, 12H), 3.15 – 2.35 (m, 8H), 2.32 – 1.94 (m, 4H), 1.39 – 0.50 (m, 28H). ^{13}C NMR (100 MHz, $CDCl_3$) δ 173.26, 170.94, 165.11, 158.51, 146.73, 139.62, 133.89, 132.29, 132.25, 127.56, 125.83, 119.13, 116.06, 95.72, 84.68, 60.22, 58.45, 56.56, 53.70, 52.73, 52.24, 51.28, 51.03, 50.14, 40.44, 39.08, 30.72, 28.36, 27.49, 26.62, 25.59, 24.36, 21.64, 18.68, 18.48, 18.44, 18.35, 17.87, 14.63, 13.96, 13.77. m/z (ESI-MS$^+$) 719.55 ([M+H]$^+$ calculated: 719.48).

K_2CO_3 (600 mg) and ethyl 2-bromoacetate (600 mg) were added to compound **B5** (90 mg) in acetonitrile (3 mL), the mixture was stirred at 50 °C for 16 h, then cooled down and filtered the solid. The filtrate was concentrated under reduced pressure and the residue was dissolved in methanol (3 mL). After adding LiOH (200 mg) in water (3 mL), the reaction mixture was stirred at room temperature for 12 h, pH was adjusted to about 7, then purified by semi-preparative HPLC and concentrated to get the product as a light yellow solid, **L5** (50 mg, 46%). ^{1}H NMR (400 MHz, CD_3OD) δ 8.42 – 6.83 (m, 6H), 4.65 – 1.51 (m, 24H), 1.27 – 0.06 (m, 28H). m/z (ESI-MS$^+$) 865.53 ([M+H]$^+$ calculated: 865.46).

Europium chloride hexahydrate (9 mg) was added to ligand **L5** (20 mg) in water (2 mL). The pH of reaction mixture was adjusted to 7.0. After refluxing for 16 h, the resulting mixture was cooled down and purified by semi-preparative HPLC and lyophilized to get the product as a yellow solid, **EuL5** (16 mg, yield 68%). *m/z* (ESI-MS$^+$) 1015.3705 ([M+2H]$^+$ calculated: 1015.3689).

2.10 Synthesis of EuL6

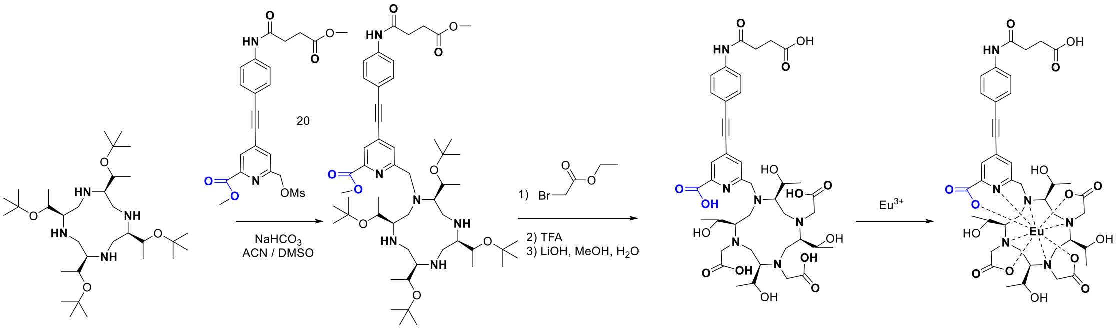

Scheme S11. Synthesis of EuL6.

Compound **A6** (373 mg) was dissolved in acetonitrile (20 mL), $NaHCO_3$ (55 mg)was added, followed by adding compound **20** (130 mg) in DMSO (1 mL) and acetonitrile (8 mL) dropwise into the reaction mixture at 50 °C slowly (10 h). Then the reaction was reacted for another 8 h. When the solution was cooled down, the solid was filtered and the filtrate was concentrated under reduced pressure to give a brown oil. This crude product was purified silica gel column chromatography by $CHCl_3$ and methanol (50:1 to 10:1) and concentrated to get the product as a light yellow solid, **B6** (200 mg, yield 32%). ^1H NMR (400 MHz, $CDCl_3$) δ 8.40 – 6.73 (m, 6H), 4.48 – 2.14 (m, 26H), 1.06 (q, *J* = 18.3, 14.5 Hz, 36H). m/z (ESI-MS$^+$) 951.74 ($[M+H]^+$ calculated: 951.65).

K_2CO_3 (400 mg) and ethyl 2-bromoacetate (210 mg) were added to compound **B6** (200 mg) in acetonitrile (5 mL), the mixture was stirred at 50 °C for 16 h, then cooled down and the solid was filtered, the filtrate was concentrated and the residue was first deprotected by TFA (3 mL) for overnight, then concentrated. The residue was dissolved in methanol (3 mL), LiOH (150 mg) in water (3 mL) was added, the reaction mixture was stirred at room temperature for 12 h. Then, the pH of resulting mixture was adjusted to about 7, and purified by semi-preparative HPLC and concentrated to get the product as a light yellow solid, **L6** (80 mg, 44%). ^1H NMR (400 MHz, CD_3OD) δ 8.44 – 7.24 (m, 6H), 4.70 – 1.92 (m, 22H), 1.66 – 0.63 (m, 12H). m/z (ESI-MS$^+$) 873.46 ($[M+H]^+$ calculated: 873.38).

Europium chloride hexahydrate (9 mg) was added to ligand **L6** (20 mg) in water (2 mL), the pH was adjusted to 7.0. After refluxing for 16 h, the temperature was cooled down and the resulting mixture was purified by semi-preparative HPLC and lyophilized to get the product as a yellow solid, **EuL6** (17 mg, yield 75%). m/z (ESI-MS$^-$) 1021.2720 ($[M]^-$ calculated: 1021.2703).

2.11. Synthesis of peptoid

Fmoc-protected Rink Amide resin (100–300 mg, 0.1–0.2 mmol, typical loading between 0.6 – 0.8 mmol g^{-1}) was swollen in DMF (overnight, at RT) in a 20 mL polypropylene syringe fitted with two polyethylene frits. The resin was deprotected with piperidine (20 % in DMF *v/v*, 2 x 20 min) and washed with DMF (5 x 2 mL). The resin was treated with bromoacetic acid (2 mL, 0.6 M in DMF) and DIC (0.20 mL, 50 % *v/v* in DMF) for 20 minutes at room temperature at 400 rpm. The resin was washed with DMF (5 x 2 mL), before the desired amine submonomer was added (2 mL, 0.8–2.0 M in DMF) and allowed to react for 60 minutes at room temperature on a block shaker at 400 rpm. The resin was again washed with DMF (5 x 2 mL) and the bromoacetylation and amine displacement steps were repeated until the final submonomer had been added and the desired peptoid sequence had been

obtained[12].

The linear α-peptoid was synthesised via manual SPPS, as described above and the glycine spacer added as follows. The peptoid on resin was swollen in DMF in a polypropylene syringe fitted with a polyethylene frit (overnight, at RT). DIPEA (5 equivalents with respect to the resin) was added to a solution Fmoc-protected glycine (5 equivalents with respect to the resin, dissolved in the minimum amount of DMF) and PyBOP (5 equivalents with respect to the resin in DMF). The DIPEA/PyBOP/glycine solution (2.0 mL) was then added to the resin bound peptoid and left for 1 hour at 25 ˚C on a block shaker at 400 rpm. The resin was washed with DMF (5 x 2 mL) and treated with piperidine (20 % in DMF v/v, 2 x 20 minutes) and then washed with DMF (5 x 2 mL). To prepare the glycine-glycine spacer, the procedure was repeated and a second Fmoc-Gly-OH was added.

A test cleavage of the linear α-peptoid-peptide hybrid Fmoc-Gly-Gly-*N*pmb-*N*Lys-Npcb-*N*Lys-NH_2 was carried out and LC mass spectroscopy confirmed that the target molecule had been prepared – observed *m/z* 969.5 = $[M + H]^+$

2.12. Synthesis of EuL7

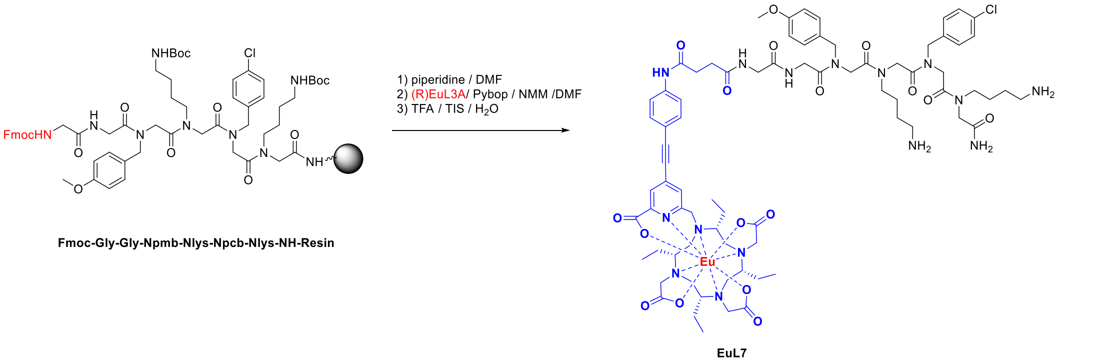

Scheme S12. Synthesis of EuL7.

EuL7 was synthesized from the complex **(*R*)(SAP)EuL3** with the peptoid, Fmoc-Gly-Gly-Npmb-Nlys-Npcb-Nlys-NH-Resin through the above peptoid synthesis. In a peptoid synthesis reactor, the peptoid on resin (0.01 mmol, 20 mg) was firstly deprotected by piperidine/DMF (1:5) (1 mL) for 20 min. Then the resin was washed with DMF (3 mL) and the solvent was purged by nitrogen, followed by adding DMF (1 mL), **(*R*)(SAP)EuL3** (4.6 mg), NMM (2 □L) and PyBop (5 mg). After reacting for 2 h, the solid was filtered and the filtrate was washed by DMF (5 mL). Then, the resulting resin was purged by nitrogen, it became very bright under UV lamp. Then the deprotection was done by adding 95% TFA / 3% water / 2% TIPS (1 mL) at room temperature for 1.5 h. The solid was filtered and the filtrate was concentrated under reduced pressure. The harsh conditions (95% TFA) used in the synthesis also confirms the high stability of these Eu(III) complexes where no decomplexation was observed (Figure S72). The residue was purified by semi-preparative HPLC and lyophilized to get the pure compound as a yellow solid, **EuL7** (2 mg, 25%). m/z 1/2(ESI-MS$^+$) 843.8357 (1/2$[M+3H]^{2+}$ calculated: 843.8351).

3. Photophysical measurements

3.1 Absorption, excitation and emission of EuL1-7

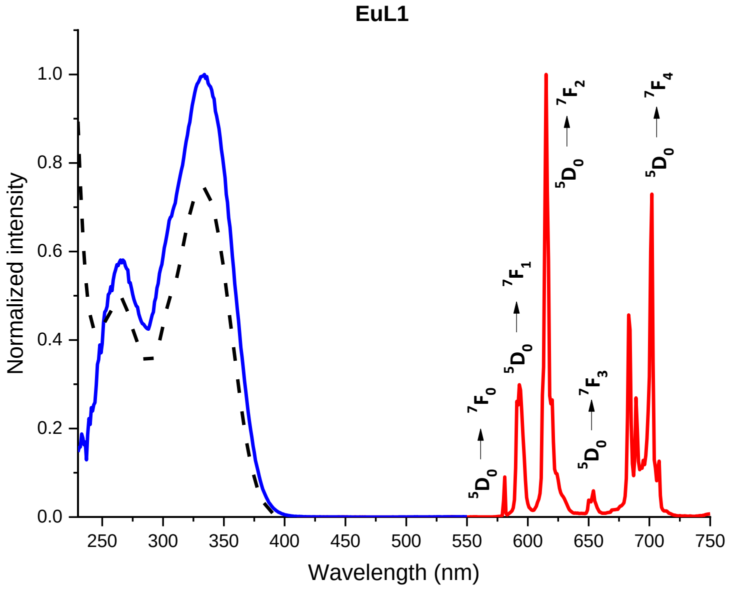

Figure S1. Absorption (blue, solid line), excitation (black, dash line, λ_{em} = 616 nm) and emission spectra (red, solid line, λ_{ex} = 350 nm, with 380 nm long pass filter) of EuL1 in 0.1 M HEPES buffer, pH 7.3.

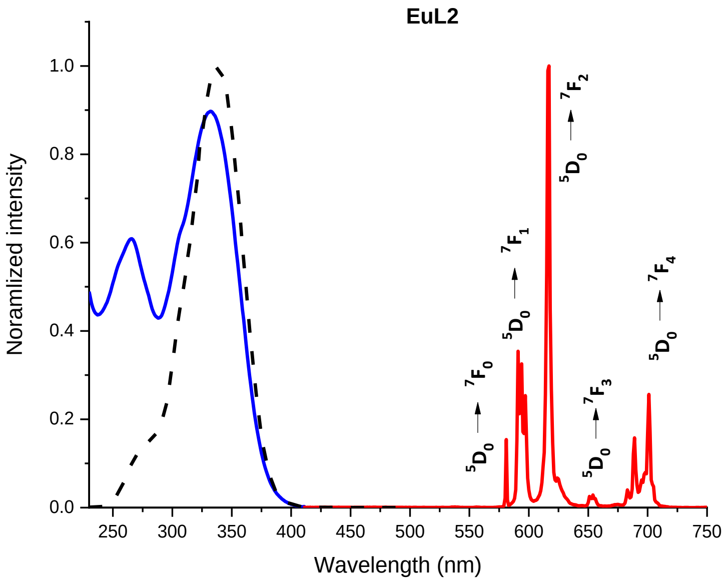

Figure S2. Absorption (blue, solid line), excitation (black, dash line, λ_{em} = 616 nm) and emission spectra (red, solid line, λ_{ex} = 350 nm, with 380 nm long pass filter) of EuL2 in 0.1 M HEPES buffer, pH 7.3.

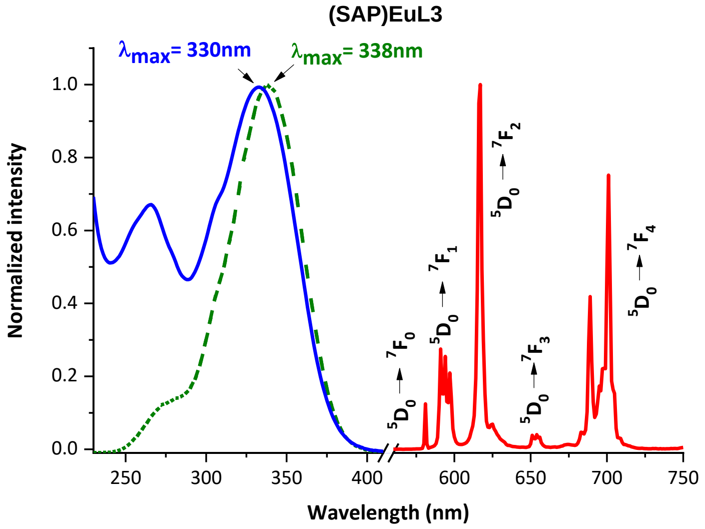

Figure S3. Absorption (blue, solid line), excitation (black, dash line, λ_{em} = 616 nm) and emission spectra (red, solid line, λ_{ex} = 350 nm, with 380 nm long pass filter) of (SAP)EuL3 in 0.1 M HEPES buffer, pH 7.3.

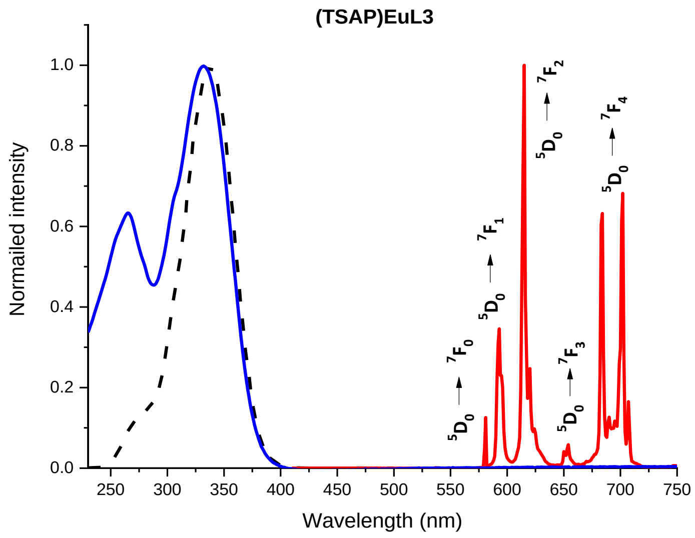

Figure S4. Absorption (blue, solid line), excitation (black, dash line, λ_{em} = 616 nm) and emission spectra (red, solid line, λ_{ex} = 350 nm, with 380 nm long pass filter) of (TSAP)EuL3 in 0.1 M HEPES buffer, pH 7.3.

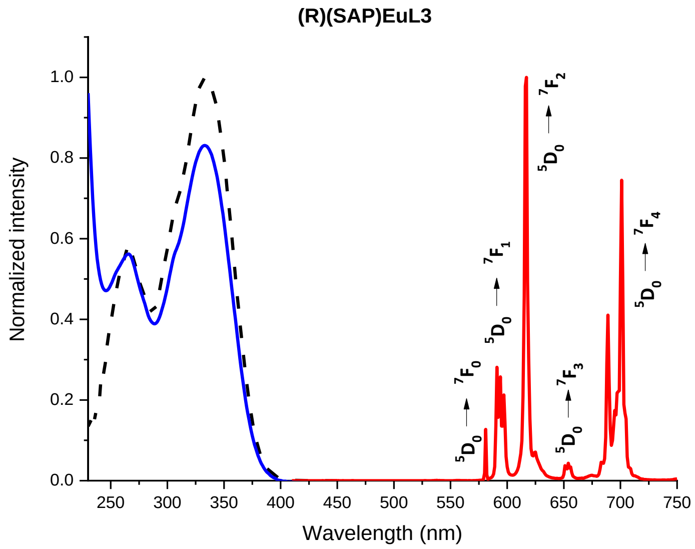

Figure S5. Absorption (blue, solid line), excitation (black, dash line, λ_{em} = 616 nm) and emission spectra (red, solid line, λ_{ex} = 350 nm, with 380 nm long pass filter) of (*R*)(SAP)EuL3 in 0.1 M HEPES buffer, pH 7.3.

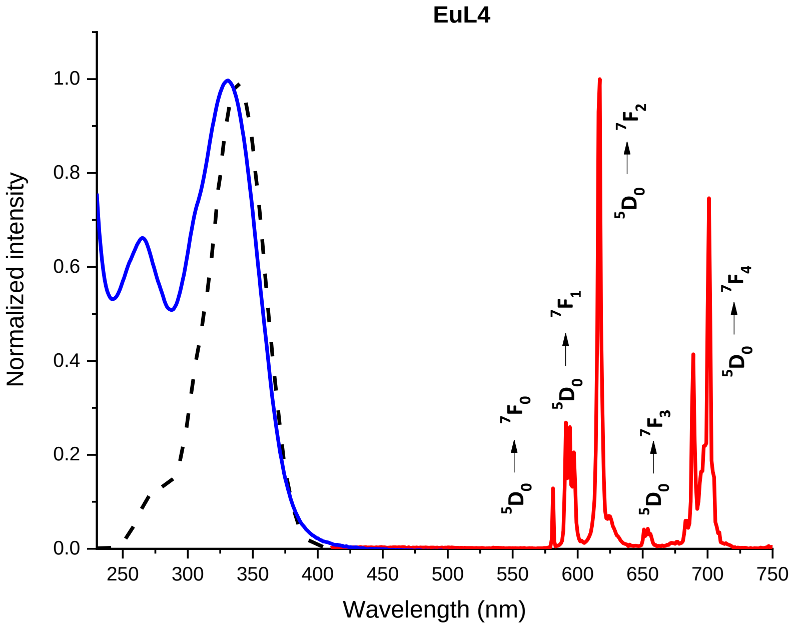

Figure S6. Absorption (blue, solid line), excitation (black, dash line, λ_{em} = 616 nm) and emission spectra (red, solid line, λ_{ex} = 350 nm, with 380 nm long pass filter) of EuL4 in 0.1 M HEPES buffer, pH 7.3.

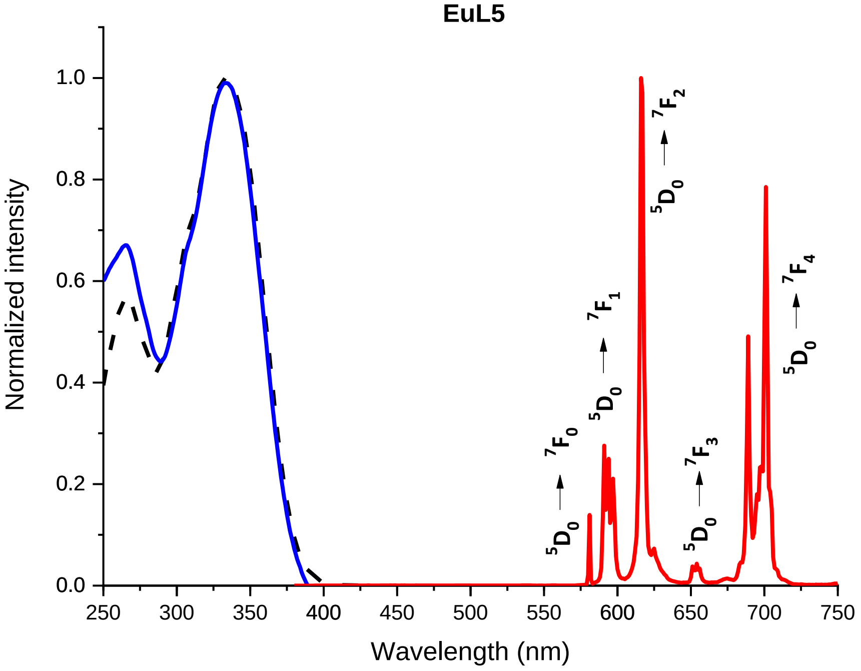

Figure S7. Absorption (blue, solid line), excitation (black, dash line, λ_{em} = 616 nm) and emission spectra (red, solid line, λ_{ex} = 350 nm, with 380 nm long pass filter) of EuL5 in 0.1 M HEPES buffer, pH 7.3.

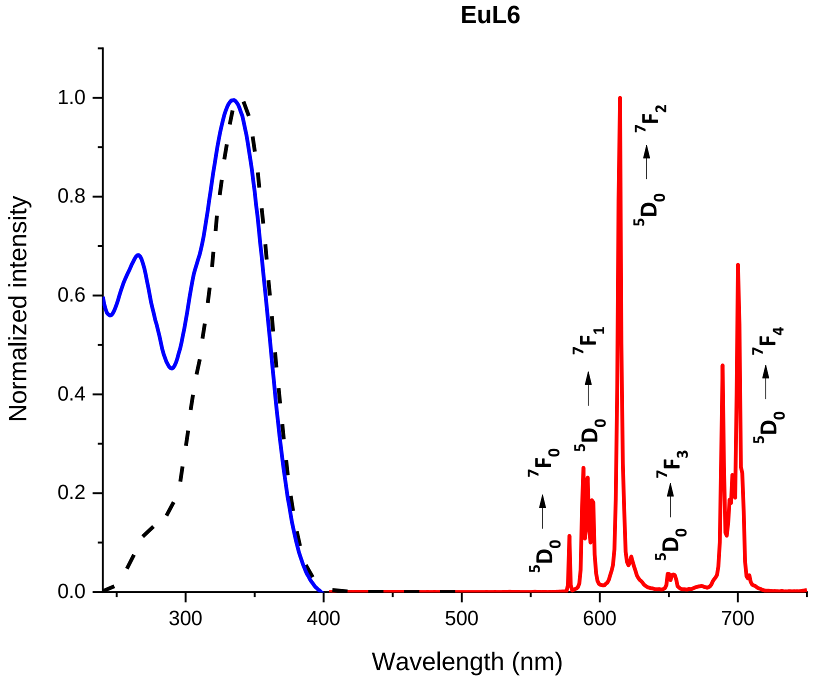

Figure S8. Absorption (blue, solid line), excitation (black, dash line, λ_{em} = 616 nm) and emission spectra (red, solid line, λ_{ex} = 350 nm, with 380 nm long pass filter) of EuL6 in 0.1 M HEPES buffer, pH 7.3.

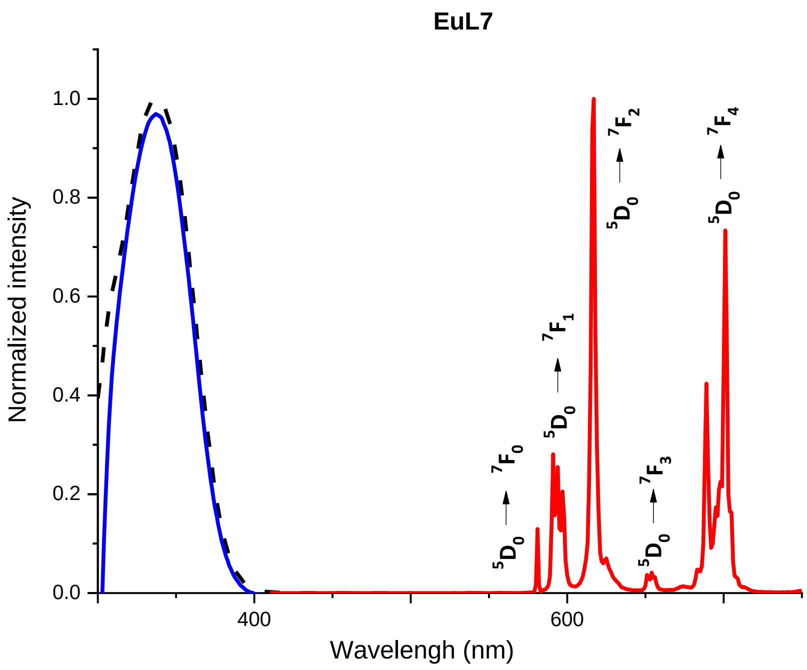

Figure S9 Absorption (blue, solid line), excitation (black, dash line, λ_{em} = 616 nm) and emission spectra (red, solid line, λ_{ex} = 350 nm, with 380 nm long pass filter) of (*R*)EuL7 in 0.1 M HEPES buffer, pH 7.3.

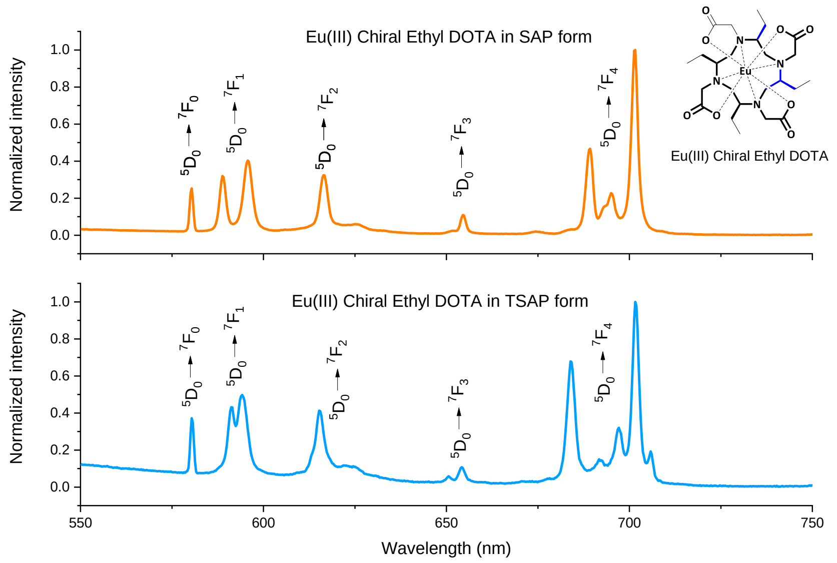

Figure S10. Emission spectra of Eu(III) chiral ethyl DOTA in SAP and TSAP geometries in D_2O (λ_{ex} = 396 nm, with 455 nm long pass filter).[11]

3.2 Low temperature measurement of GdL3A

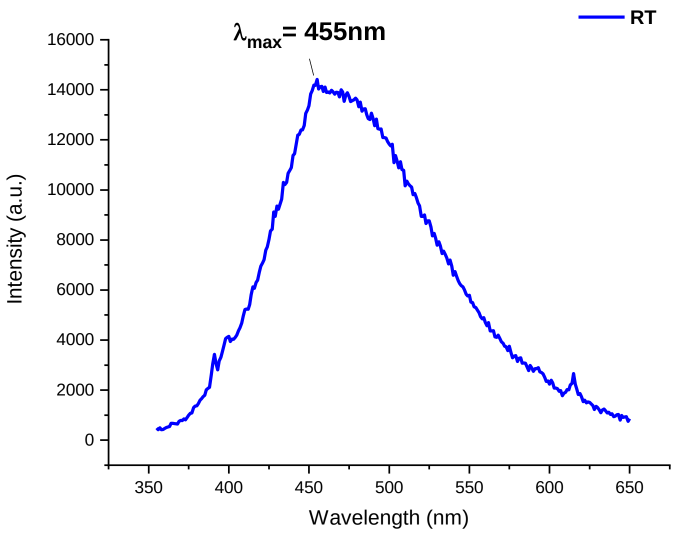

Figure S11. Emission spectrum of (SAP)GdL3 at room temperature, λ_{ex} = 350 nm, in H_2O : DMSO = 1 : 1.

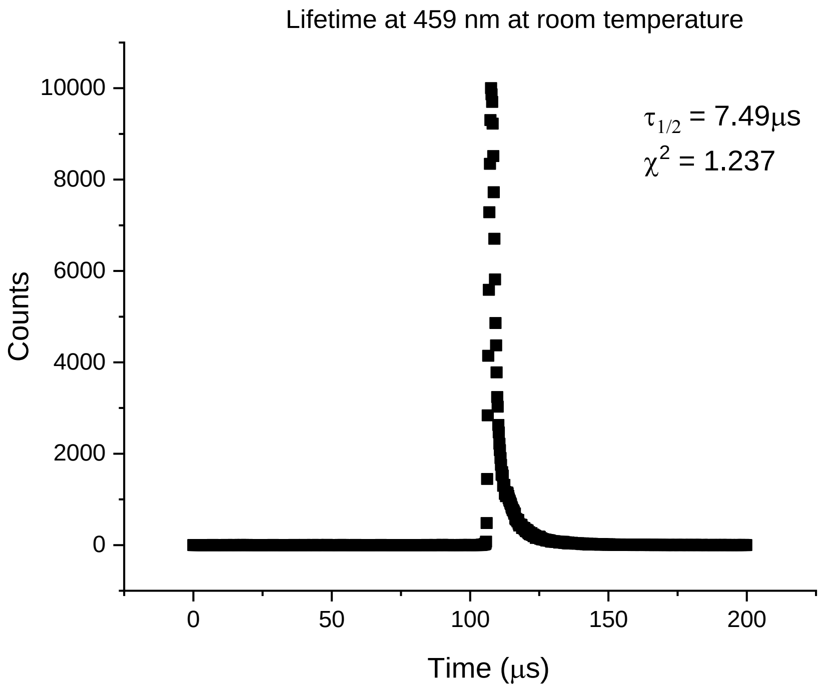

Figure S12. Excited state decay curve of (SAP)GdL3 at 459 nm at room temperature, λ_{ex} = 350 nm, in H_2O : DMSO = 1 : 1.

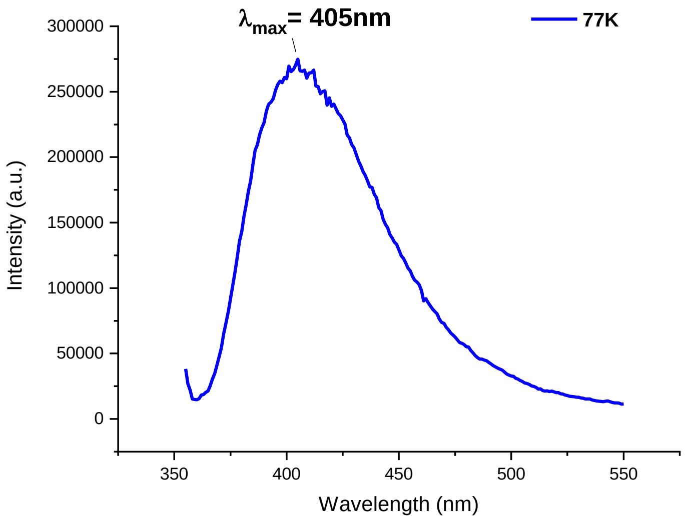

Figure S13. Emission spectrum of (SAP)GdL3 at 77 K, λ_{ex} = 350 nm, in H_2O : DMSO = 1 : 1.

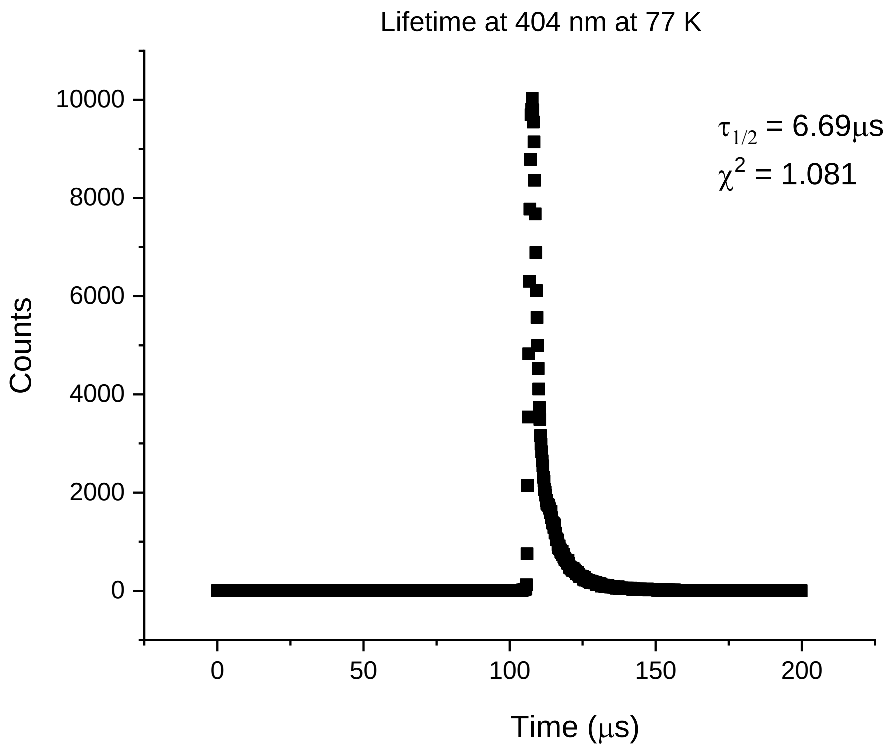

Figure S14. Excited state decay curve of (SAP)GdL3 at 404 nm at 77 K, λ_{ex} = 350 nm, in H_2O : DMSO = 1 : 1.

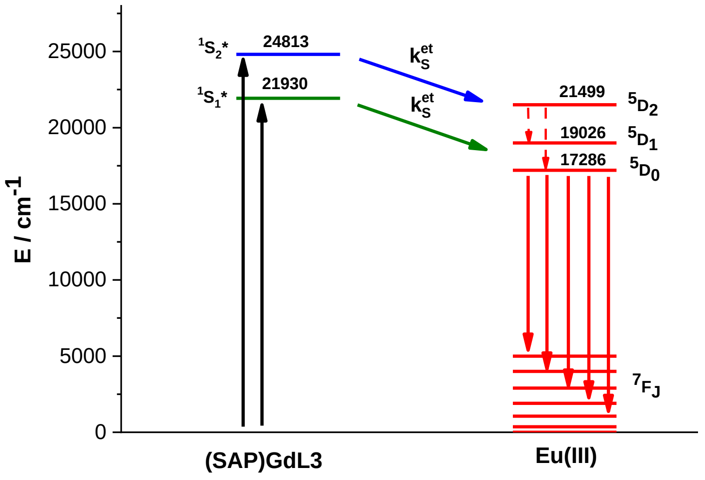

Figure S15. Jablonski diagram of (SAP)GdL3.

3.3 pH titration

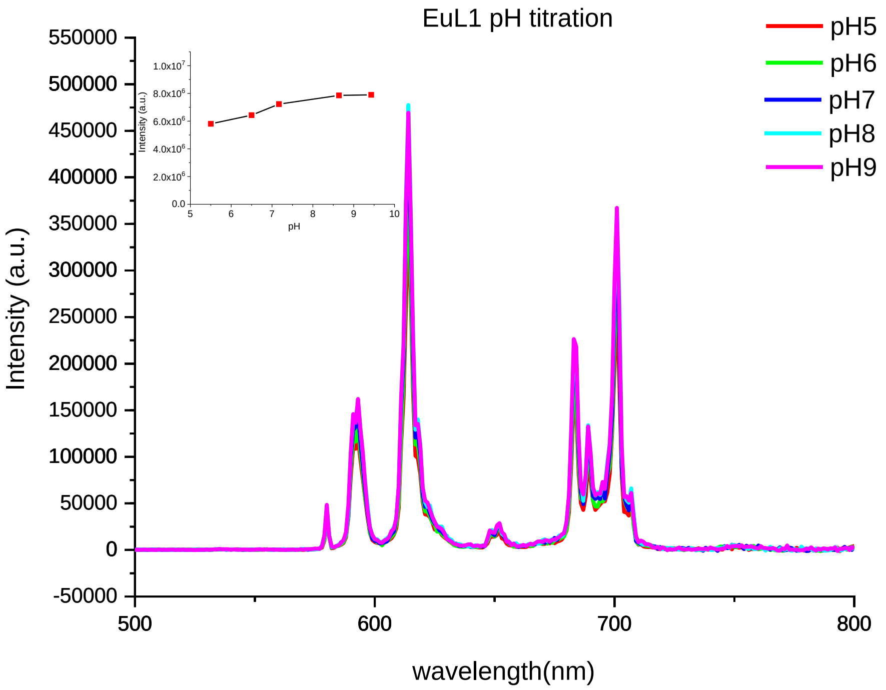

Figure S16. Emission spectra of EuL1 in different pH, λ_{ex} = 350 nm, in water.

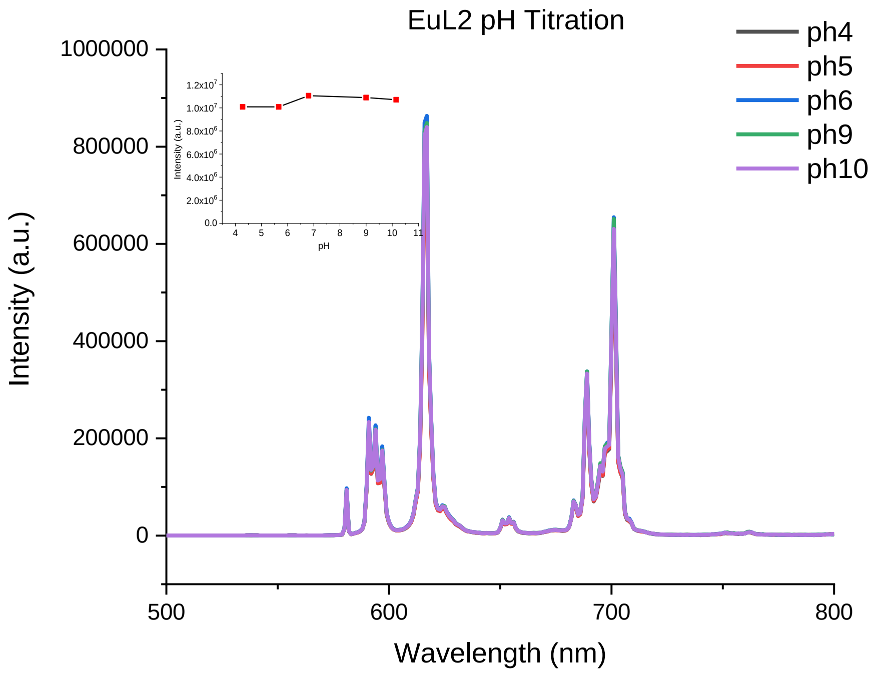

Figure S17. Emission spectra of EuL2 in different pH, λ_{ex} = 350 nm, in water.

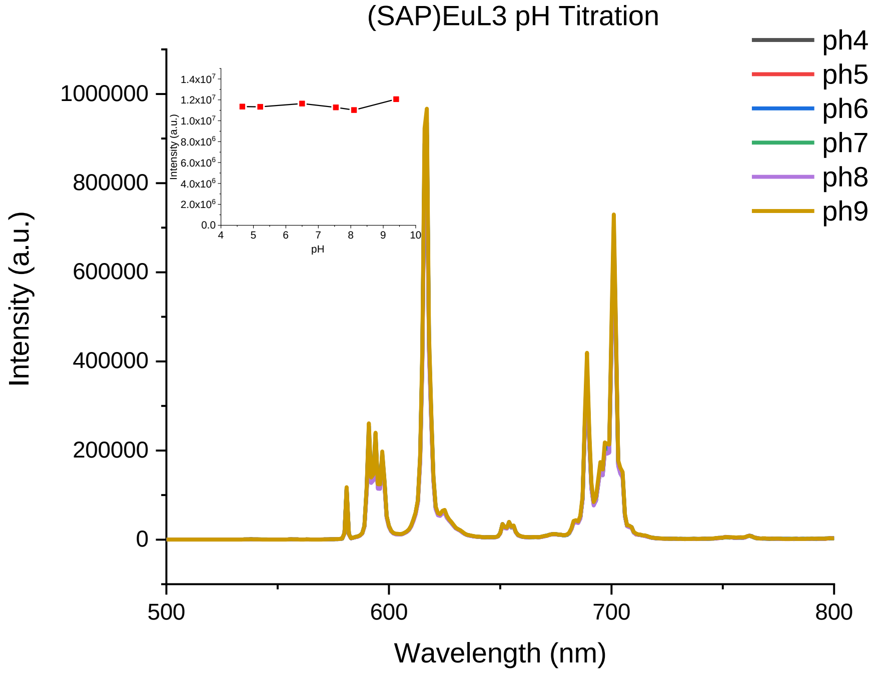

Figure S18. Emission spectra of (SAP)EuL3 in different pH, λ_{ex} = 350 nm, in water.

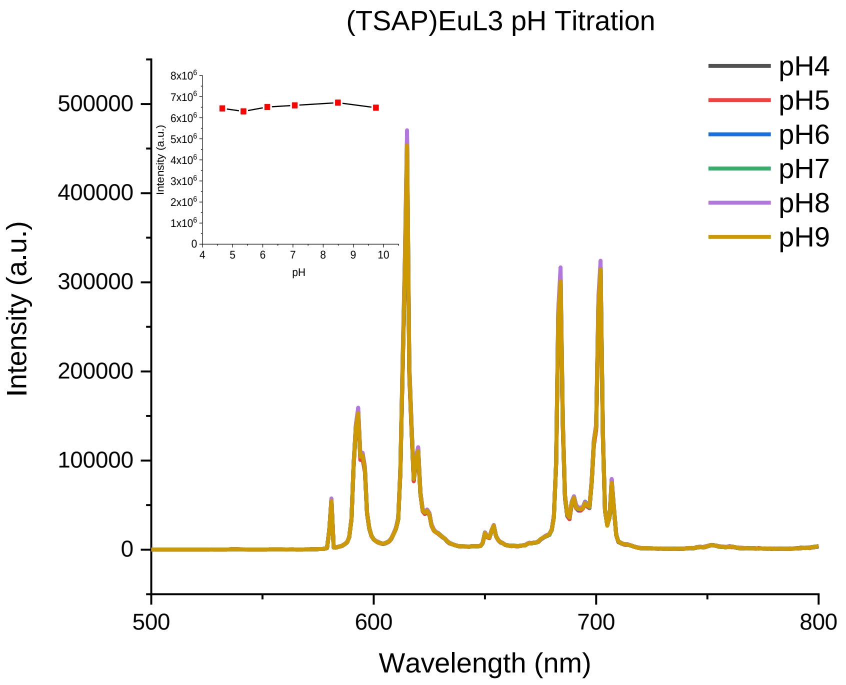

Figure S19. Emission spectra of (TSAP)EuL3 in different pH, λ_{ex} = 350 nm, in water.

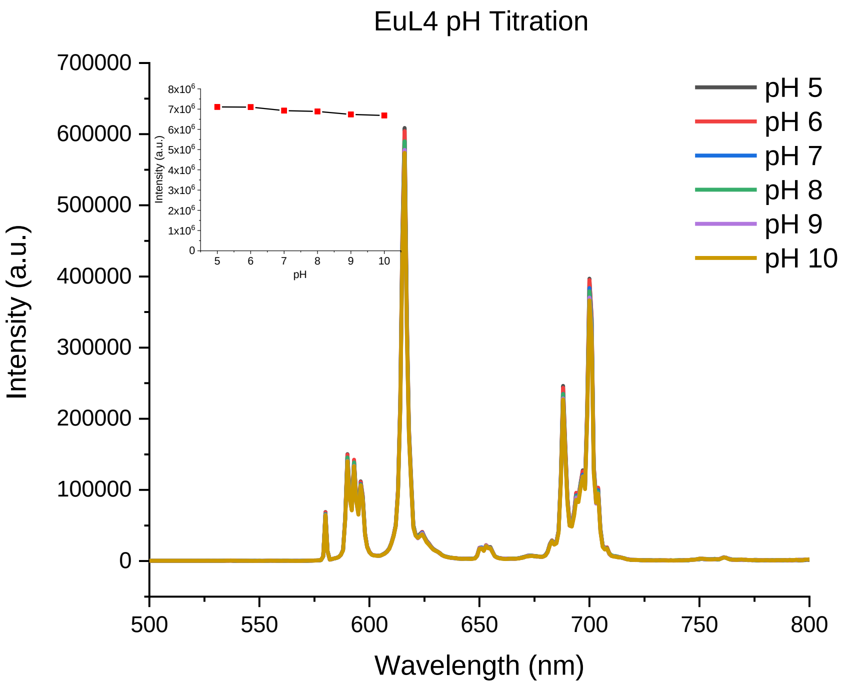

Figure S20. Emission spectra of EuL4 in different pH, λ_{ex} = 350 nm, in water.

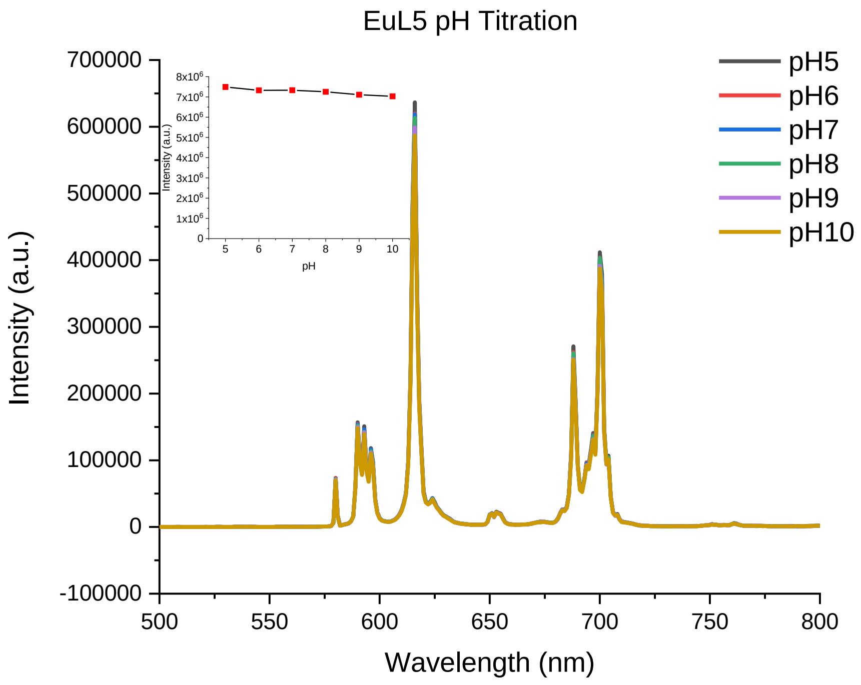

Figure S21. Emission spectra of EuL5 in different pH, λ_{ex} = 350 nm, in water.

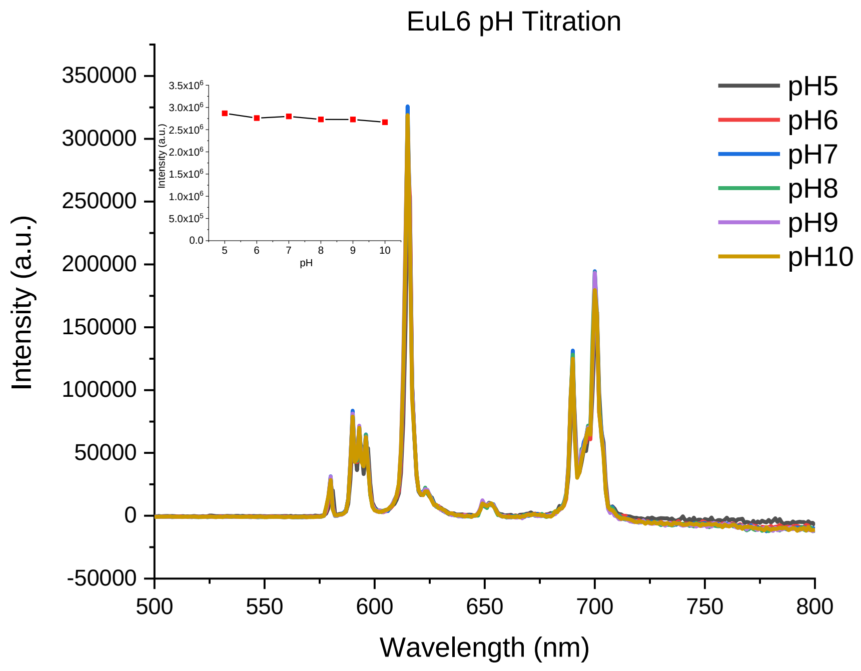

Figure S22. Emission spectra of EuL6 in different pH, λ_{ex} = 350 nm, in water.

3.4 Anion titration

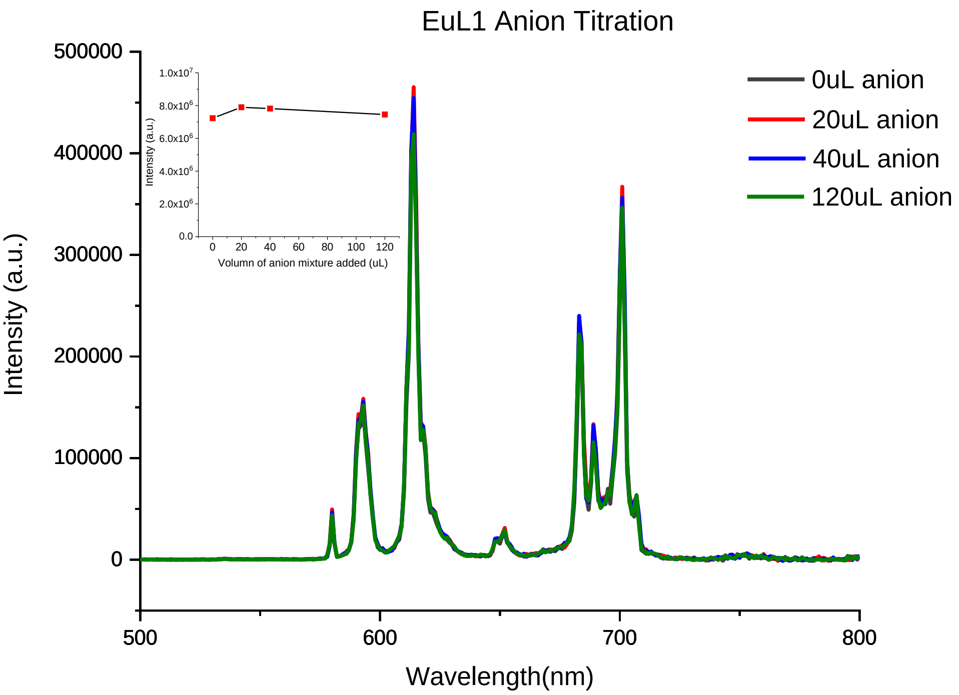

Figure S23. Emission spectra of EuL1 in anion titration.

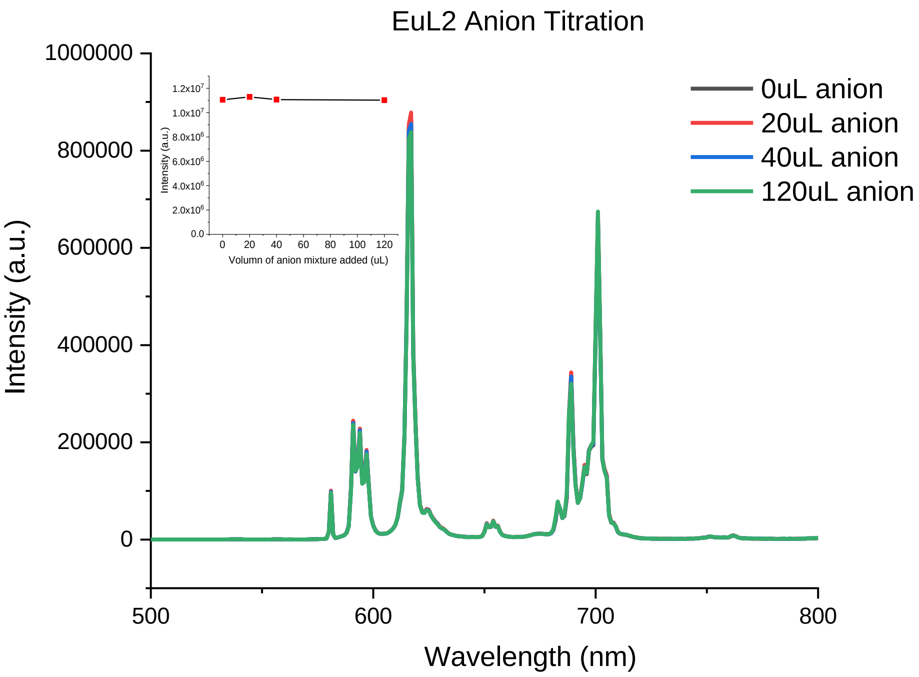

Figure S24. Emission spectra of EuL2 in anion titration.

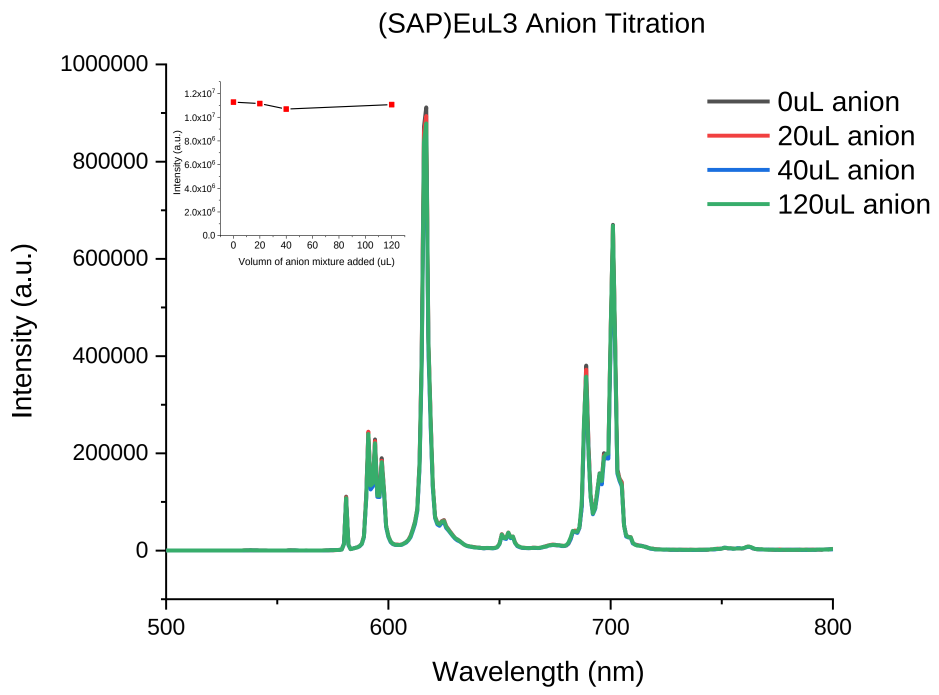

Figure S25. Emission spectra of (SAP)EuL3 in anion titration.

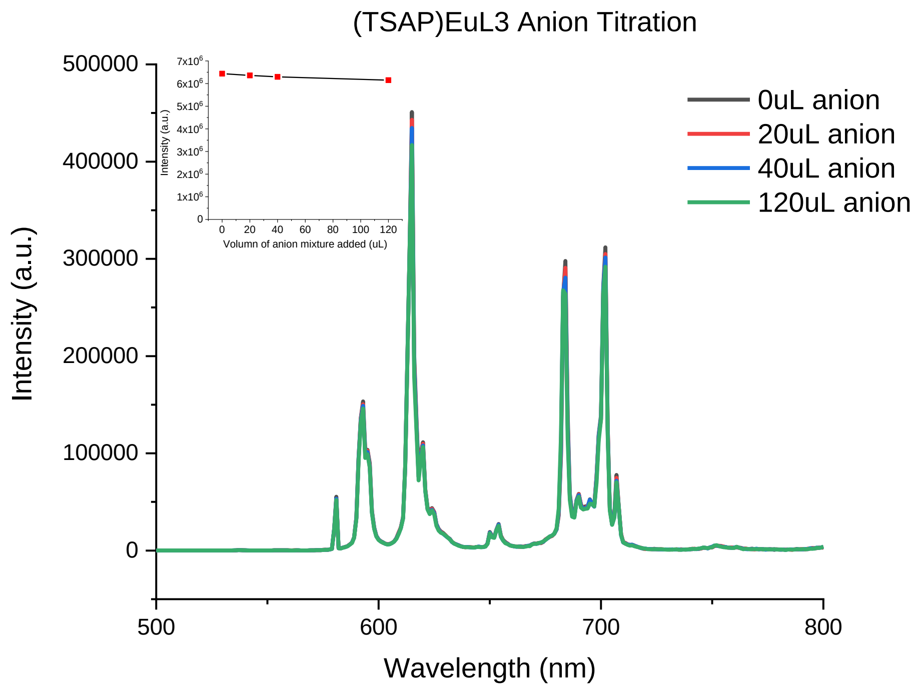

Figure S26. Emission spectra of (TSAP)EuL3 in anion titration.

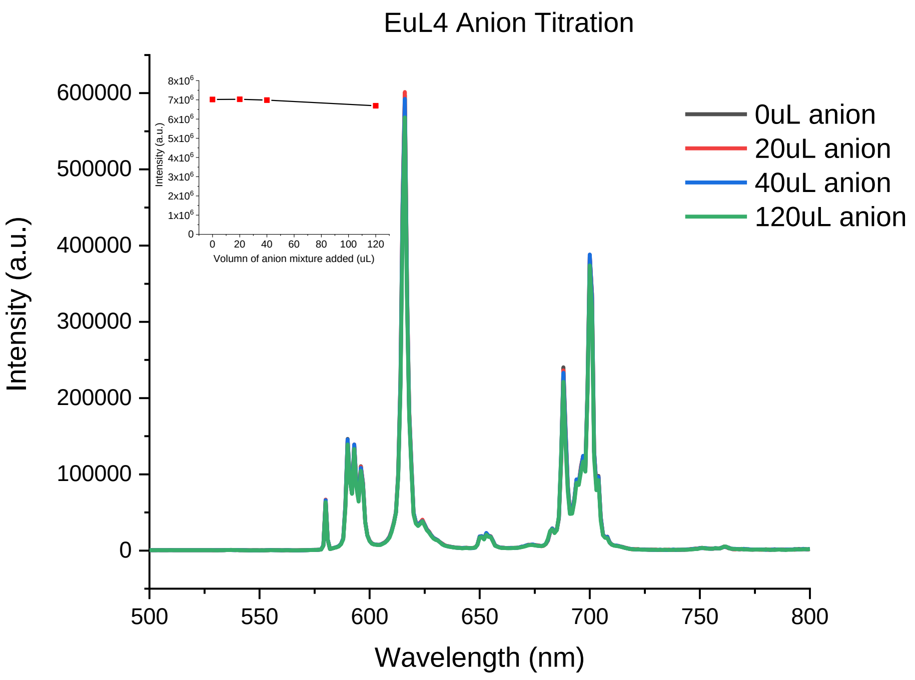

Figure S27. Emission spectra of EuL4 in anion titration.

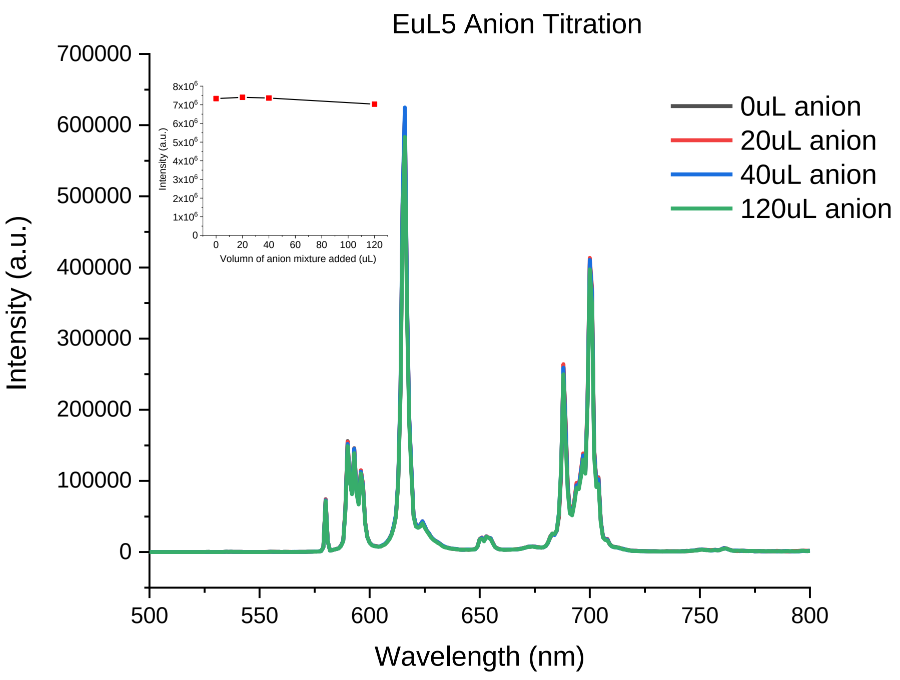

Figure S28. Emission spectra of EuL5 in anion titration.

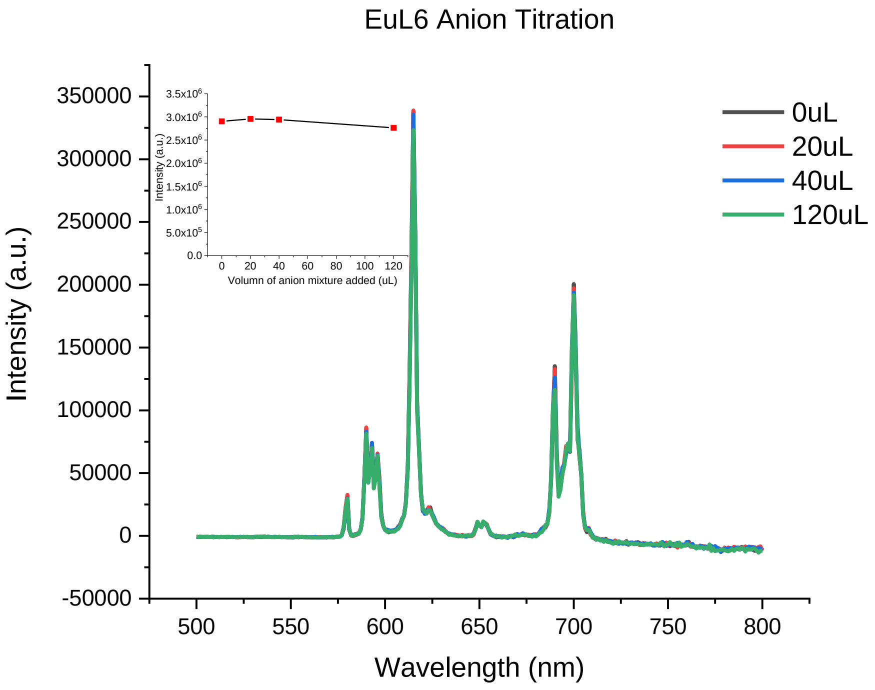

Figure S29. Emission spectra of EuL6 in anion titration.

3.5 CD spectrum

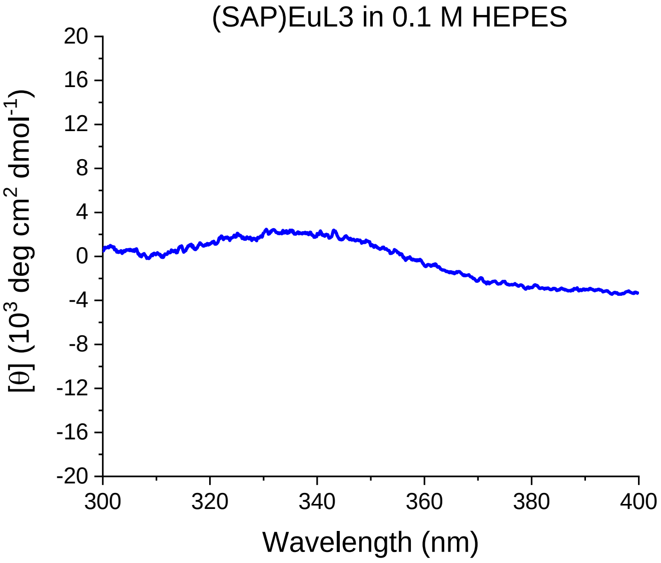

Figure S30. CD spectrum of (SAP)EuL3 in 0.1 M HEPES.

3.6 CPL spectra

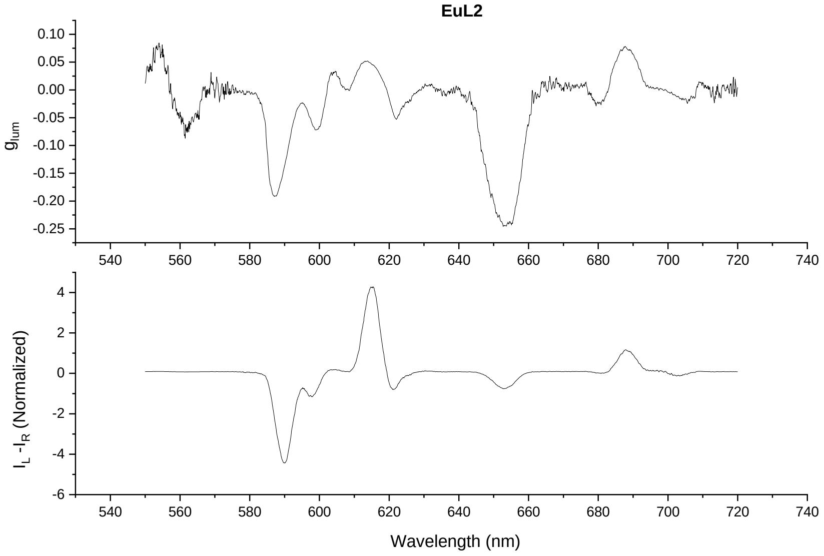

Figure S31. CPL and g_{lum} spectra of EuL2 in 0.1 M HEPES, pH 7.3, λ_{ex} = 340 nm.

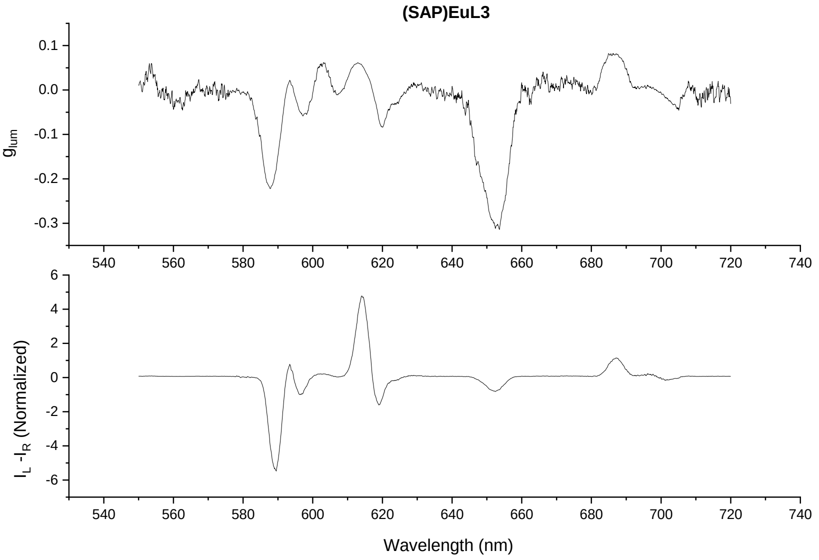

Figure S32. CPL and g_{lum} spectra of (SAP)EuL3 in 0.1 M HEPES, pH 7.3, λ_{ex} = 340 nm.

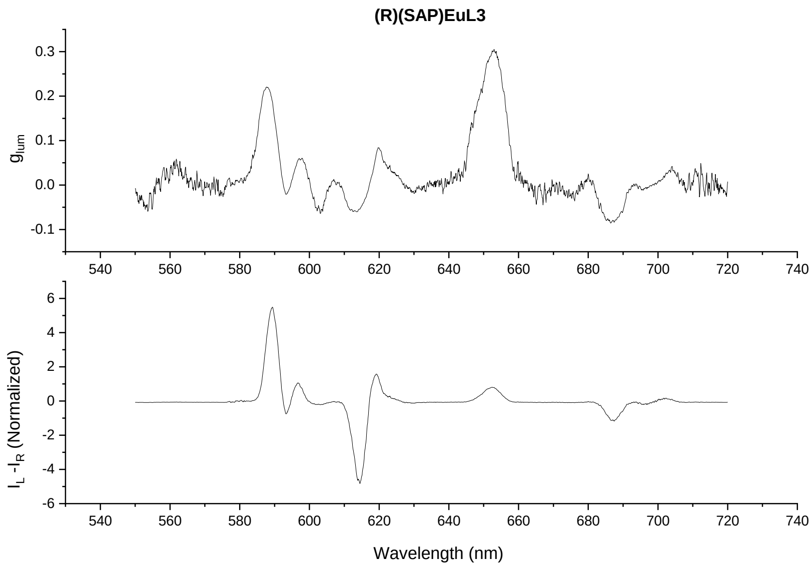

Figure S33. CPL and g_{lum} spectra of (*R*)(SAP)EuL3 in 0.1 M HEPES, pH 7.3, λ_{ex} = 340 nm.

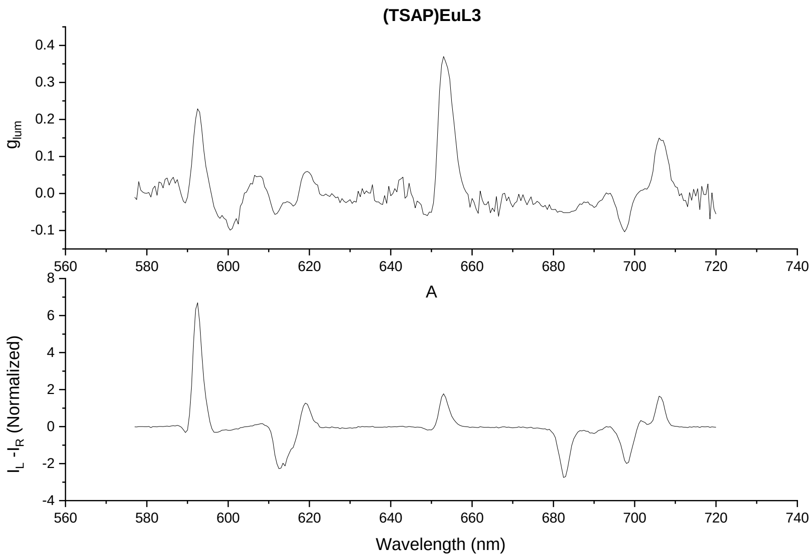

Figure S34. CPL and g_{lum} spectra of (TSAP)EuL3 in 0.1 M HEPES, pH 7.3, λ_{ex} = 340 nm.

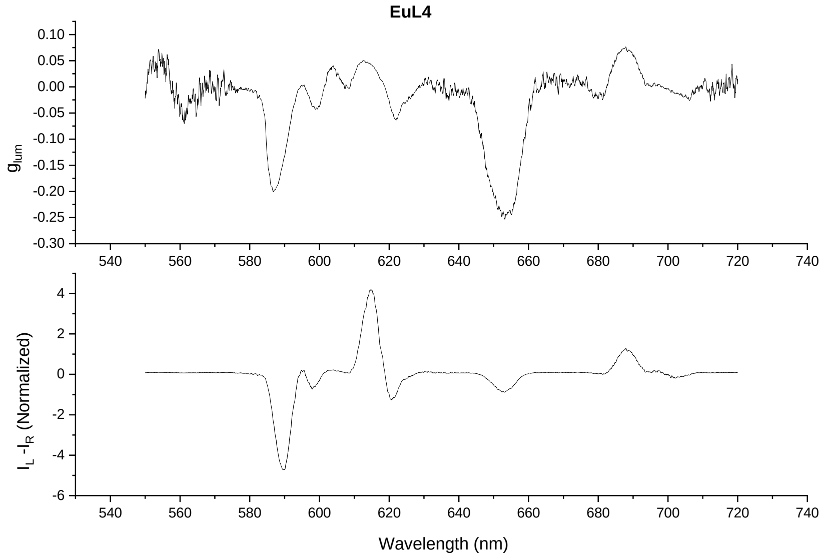

Figure S35. CPL and g_{lum} spectra of EuL4 in 0.1 M HEPES, pH 7.3, λ_{ex} = 340 nm.

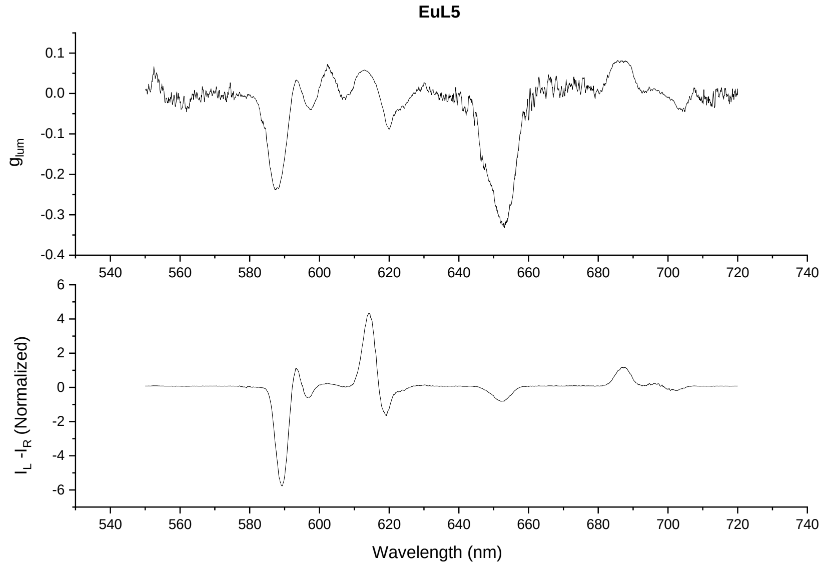

Figure S36. CPL and g_{lum} spectra of EuL5 in 0.1 M HEPES, pH 7.3, λ_{ex} = 340 nm.

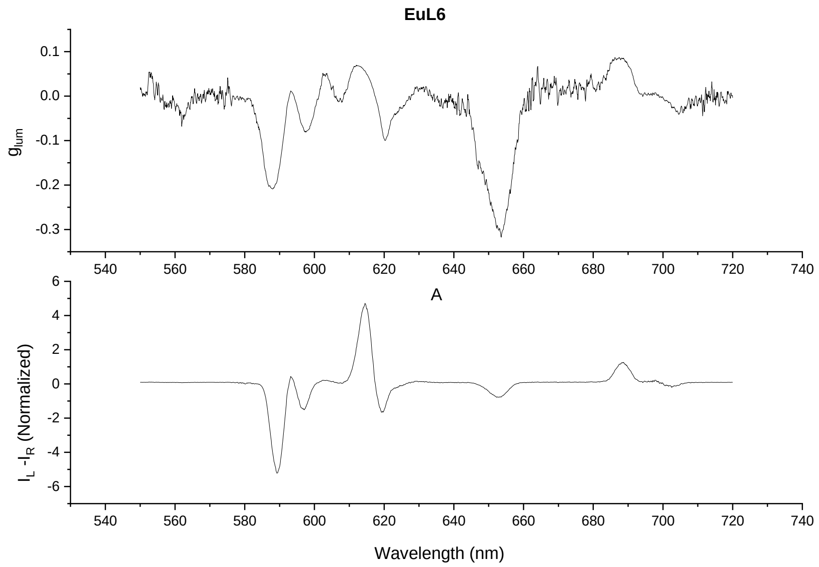

Figure S37. CPL and g_{lum} spectra of EuL6 in 0.1 M HEPES, pH 7.3, λ_{ex} = 340 nm.

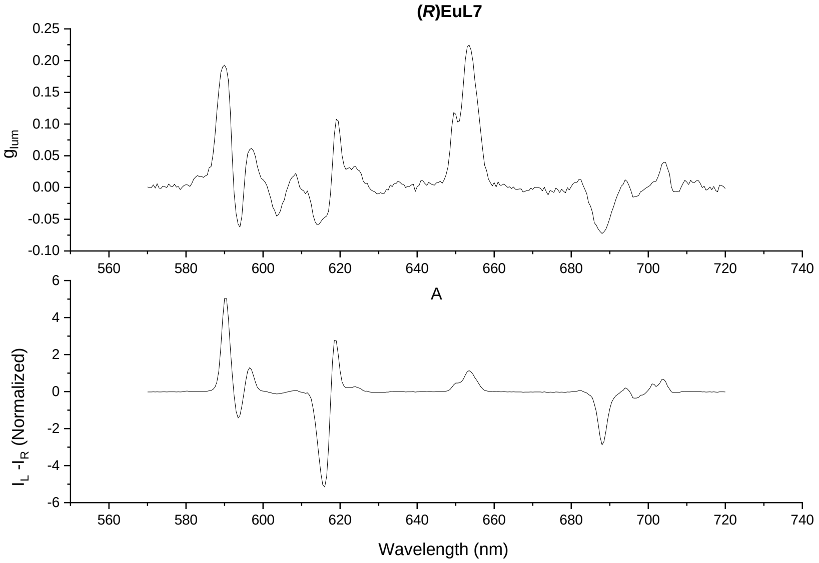

Figure S38. CPL and g_{lum} spectra of (*R*)EuL7 in 0.1 M HEPES, pH 7.3, λ_{ex} = 340 nm.

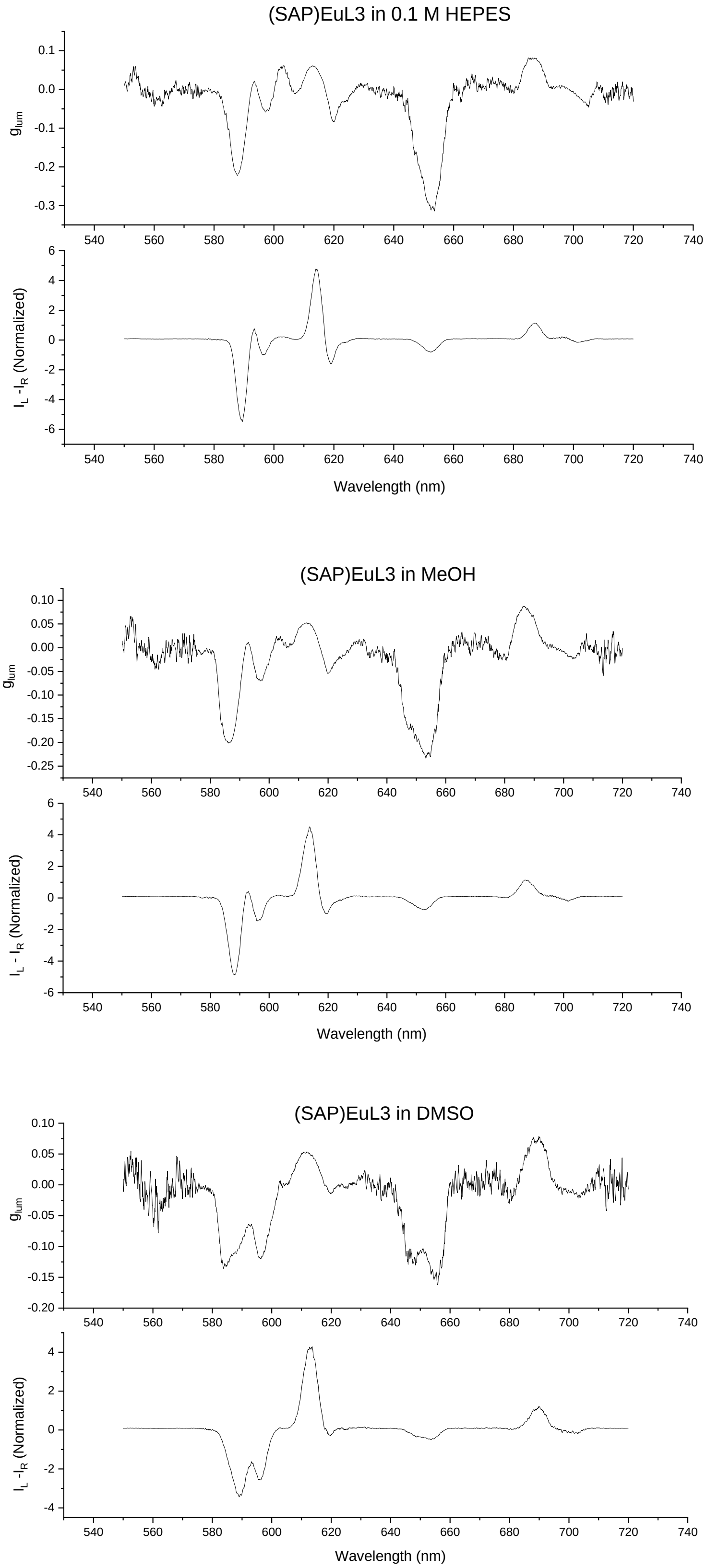

Figure S39. g_{lum} spectra of (SAP)EuL3 in different solvents.

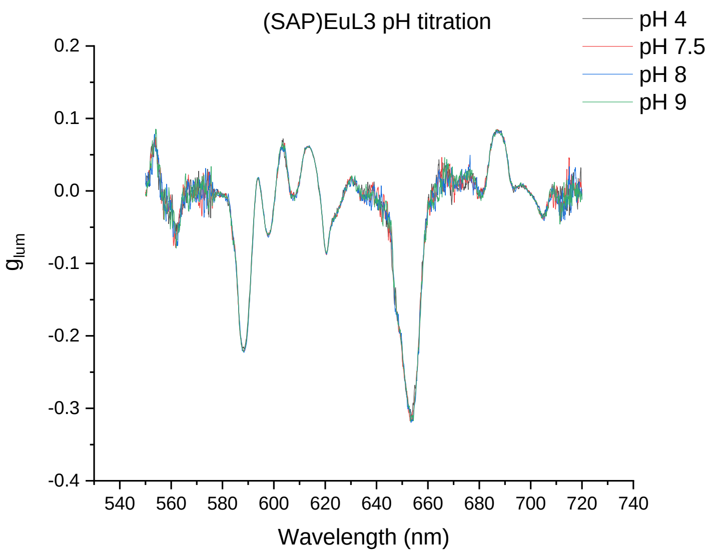

Figure S40. g_{lum} spectra of (SAP)EuL3 at different pH, in water, λ_{ex} = 340 nm.

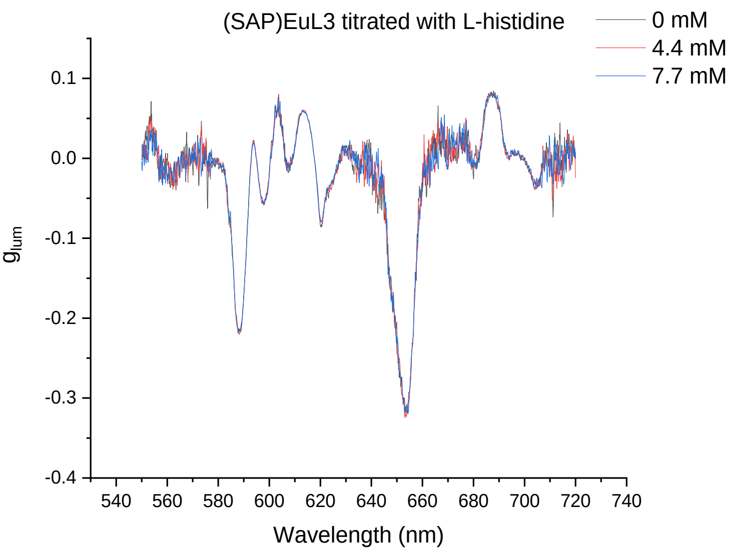

Figure S41. ***L*-Histidine titration of (SAP)EuL3 in 0.1 M HEPES, pH 7.3, λ_{ex} = 340 nm.**

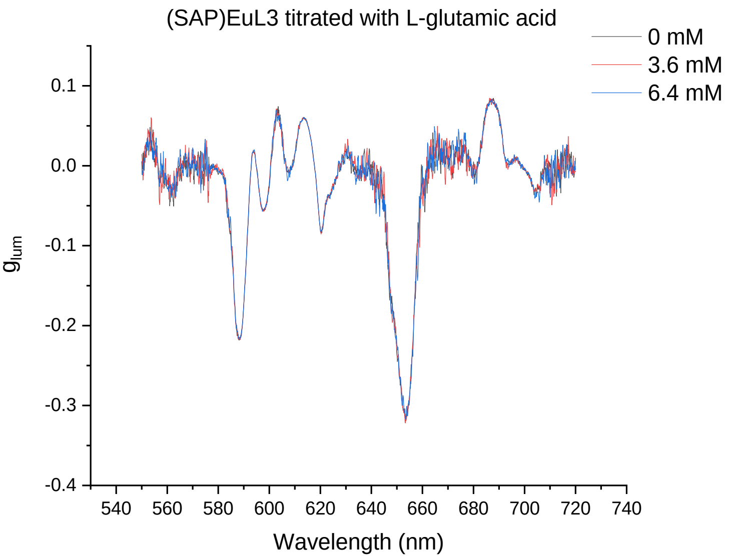

Figure S42. ***L*-Glutamic acid titration of (SAP)EuL3 in 0.1 M HEPES, pH 7.3, λ_{ex} = 340 nm.**

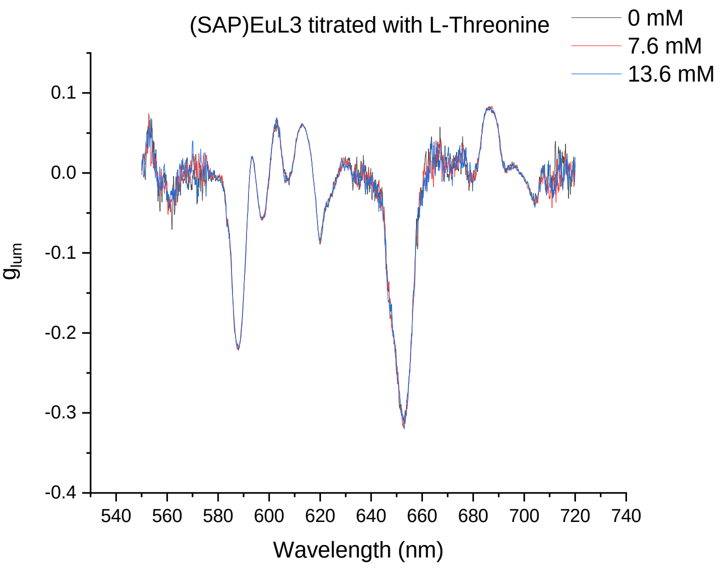

Figure S43. *L*-Threonine titration of (SAP)EuL3 in 0.1 M HEPES, pH 7.3, λ_{ex} = 340 nm.

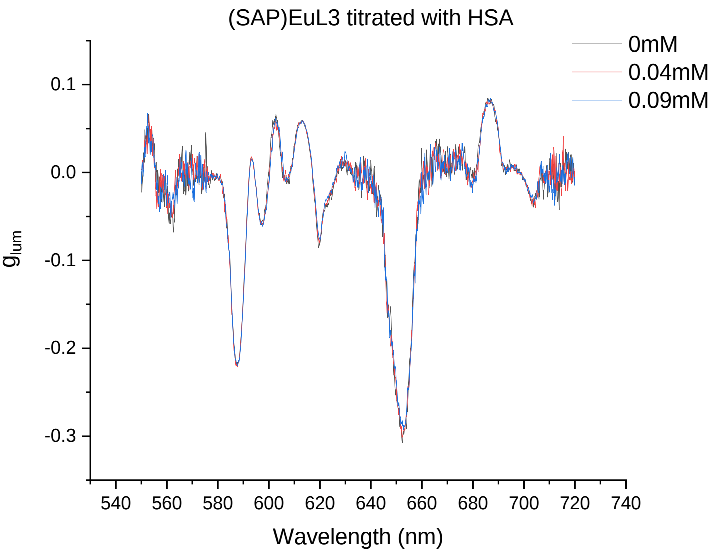

Figure S44. HSA titration of (SAP)EuL3 in 0.1 M HEPES, pH 7.3, λ_{ex} = 340 nm.

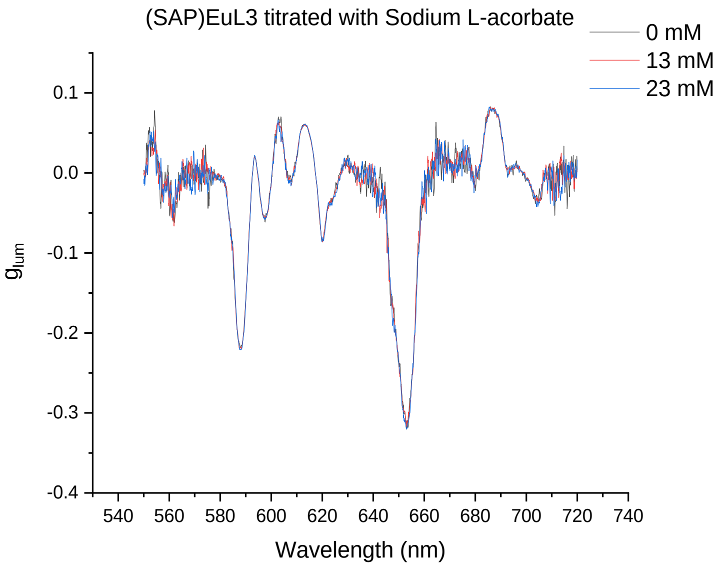

Figure S45. Sodium *L*-Ascorbate titration of (SAP)EuL3 in 0.1 M HEPES, pH 7.3, λ_{ex} = 340 nm.

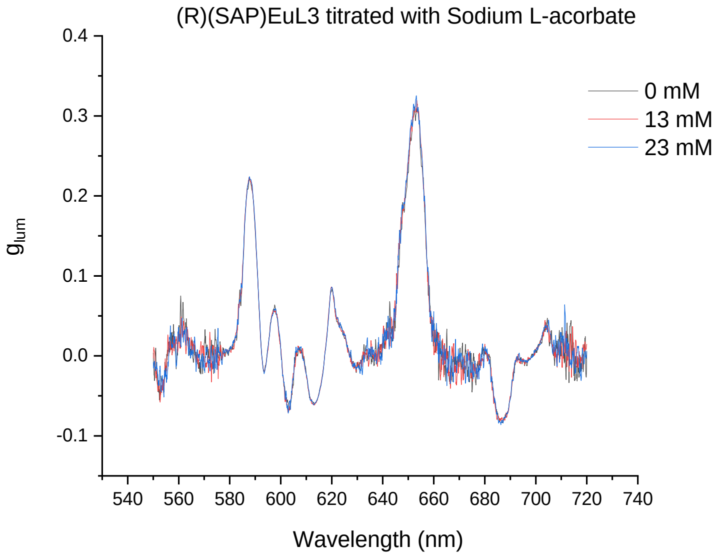

Figure S46. Sodium *L*-Ascorbate titration of (*R*)(SAP)EuL3 in 0.1 M HEPES, pH 7.3, λ_{ex} = 340 nm.

3.6 MCPL

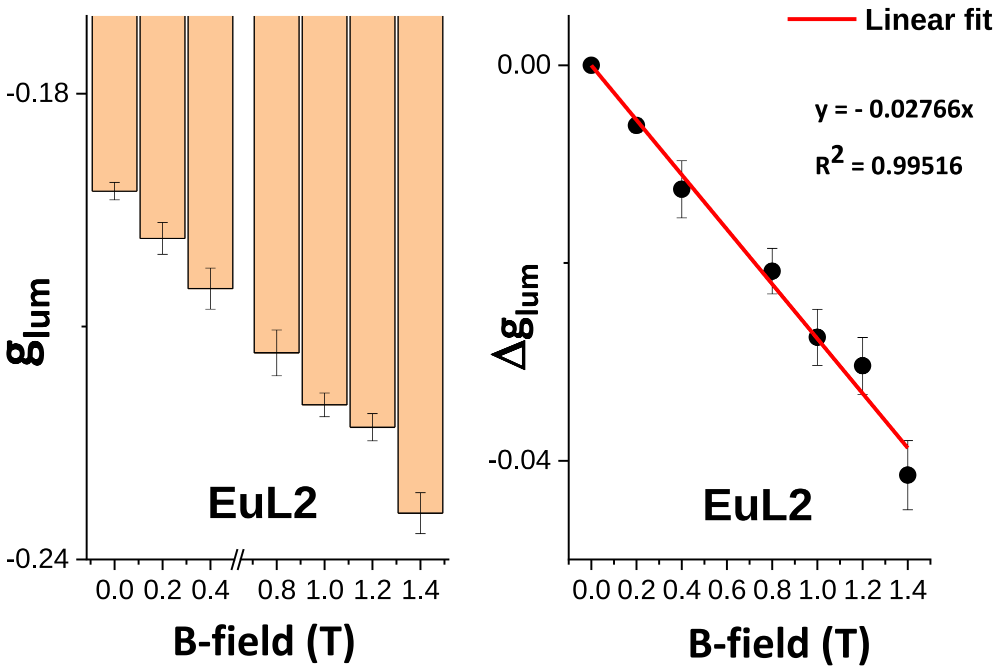

Figure S47. MCPL study of EuL2 in 0.1 M HEPES, pH 7.3, λ_{ex} = 340 nm.

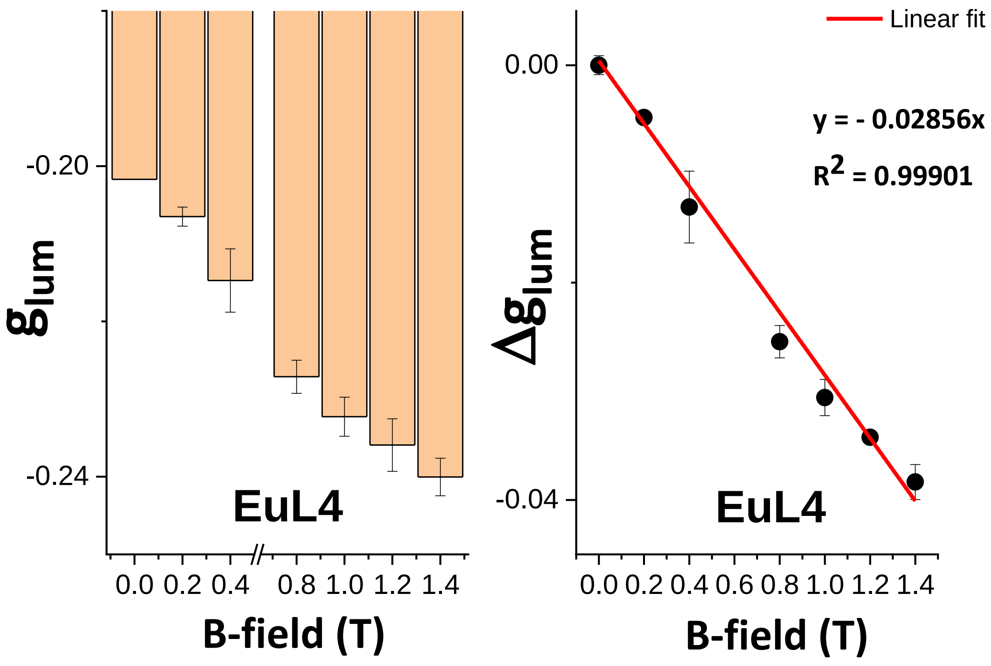

Figure S48. MCPL study of EuL4 in 0.1 M HEPES, pH 7.3, λ_{ex} = 340 nm.

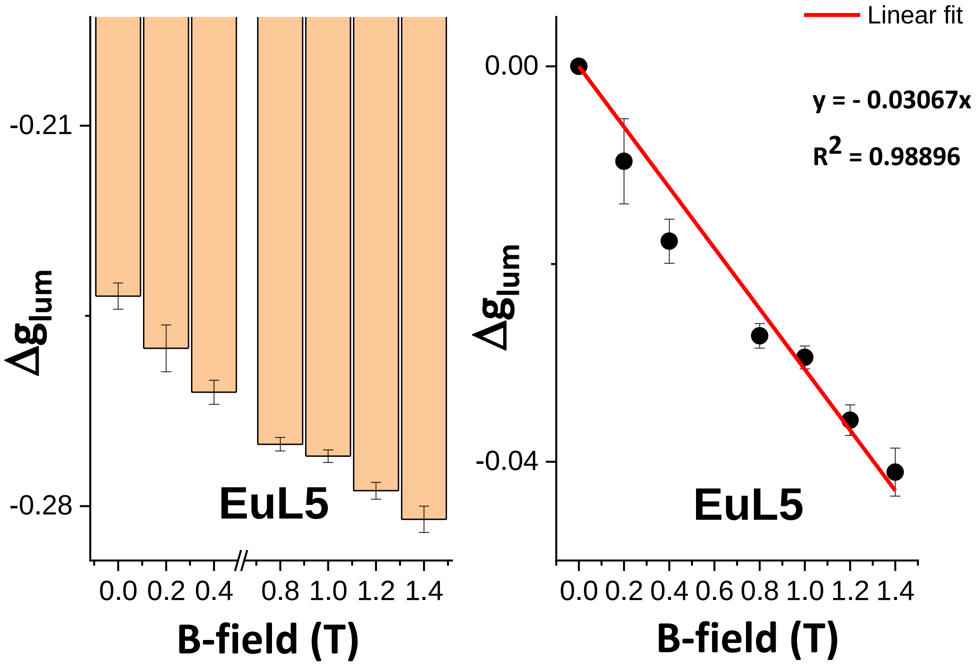

Figure S49. MCPL study of EuL5 in 0.1 M HEPES, pH 7.3, λ_{ex} = 340 nm.

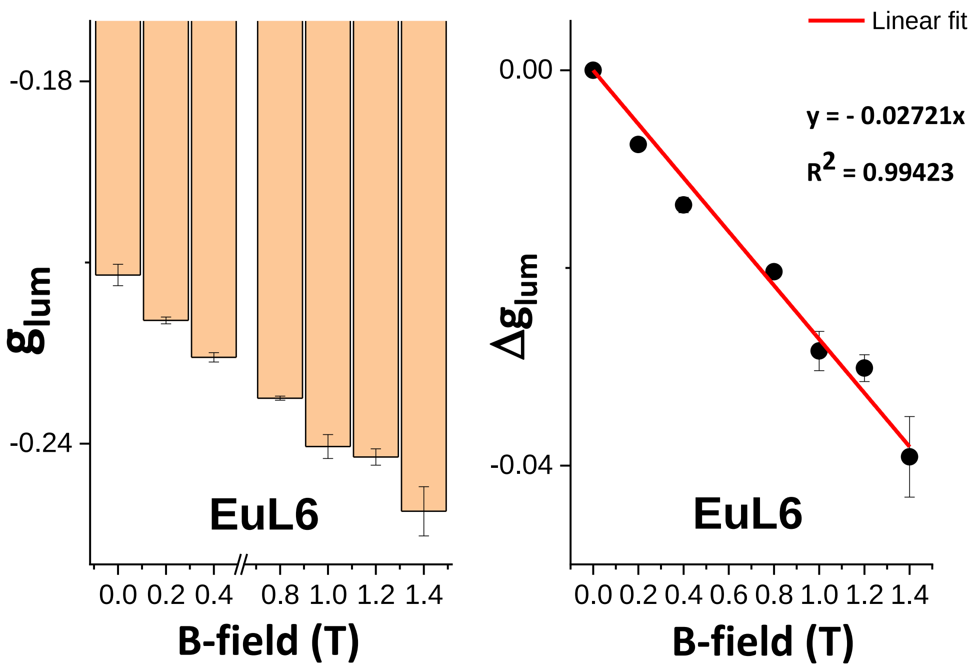

Figure S50. MCPL study of EuL6 in 0.1 M HEPES, pH 7.3, λ_{ex} = 340 nm.

4. Mass Spectra

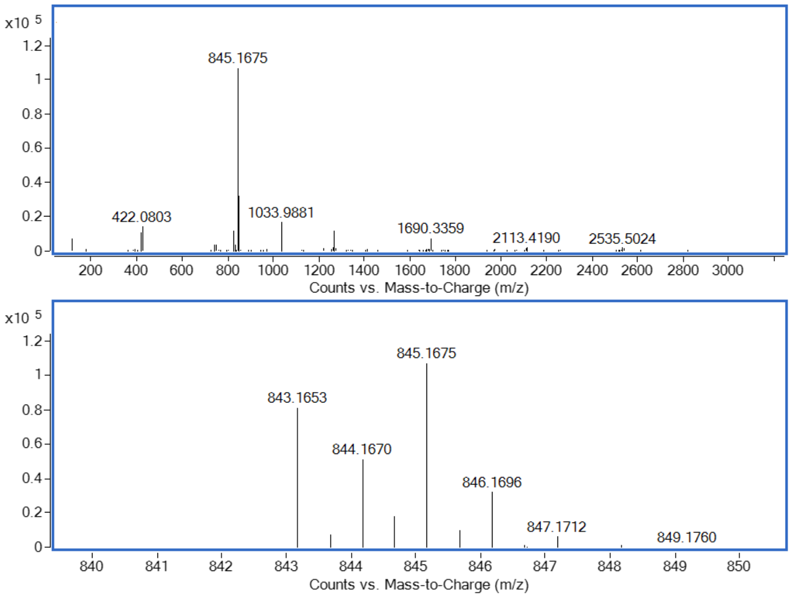

Figure S51. Mass spectrum of EuL1. m/z (ESI-MS^{-}) 845.1675 ([M]$^{-}$ calculated: 845.1654).

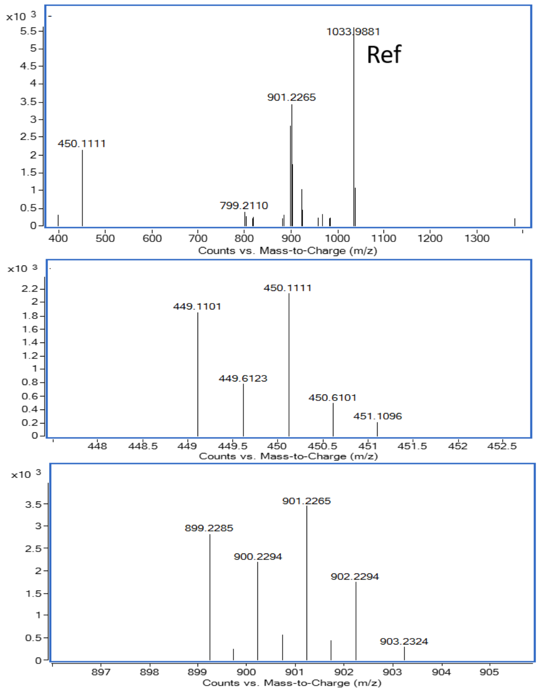

Figure S52. Mass spectrum of EuL2. m/z (ESI-MS^{-}) 901.2280 ([M]$^{-}$ calculated: 901.2265).

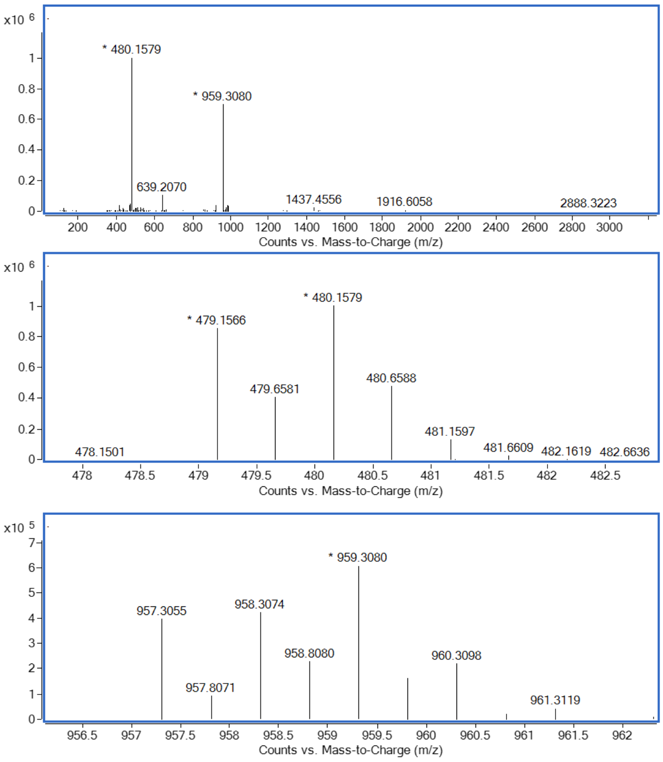

Figure S53. Mass spectrum of (SAP)EuL3. m/z (ESI-MS$^+$) 959.3080 ([M+2H]$^+$ calculated: 959.3063).

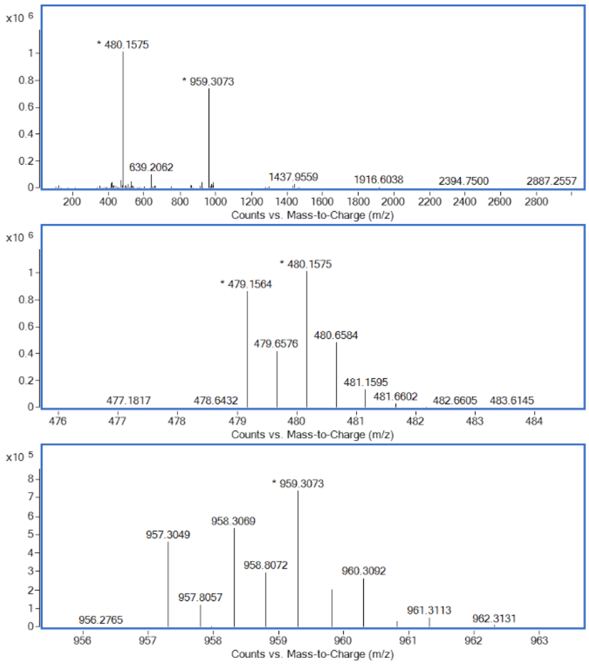

Figure S54. Mass spectrum of (TSAP)EuL3. m/z (ESI-MS$^+$) 959.3073 ([M+2H]$^+$ calculated: 959.3063).

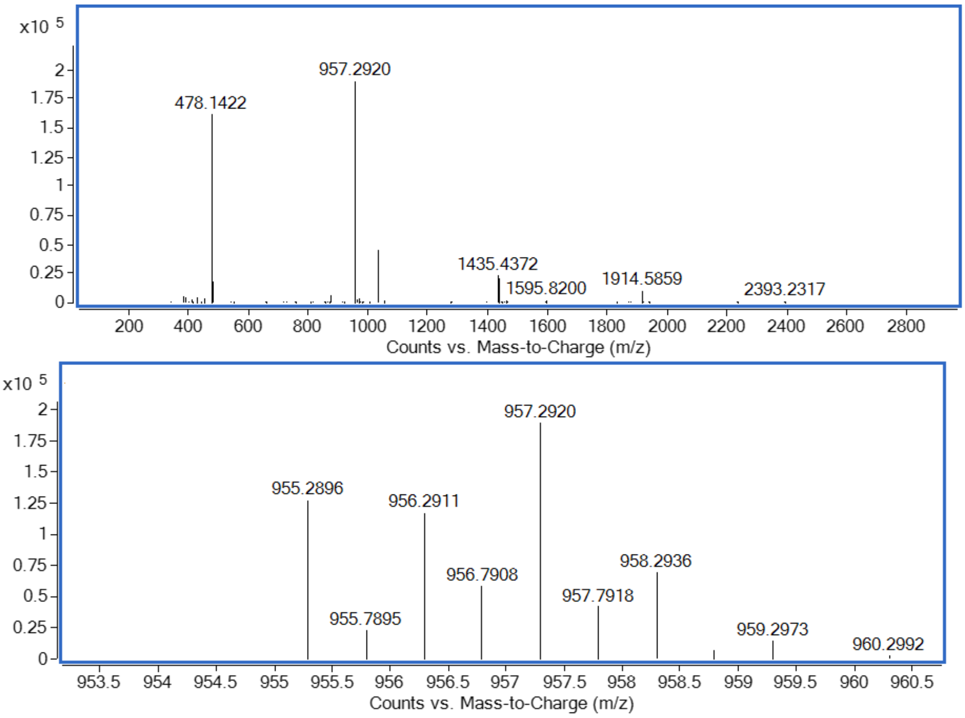

Figure S55. Mass spectrum of (*R*)(SAP)EuL3. m/z (ESI-MS$^-$) 957.2920 ([M]$^-$ calculated: 957.2907).

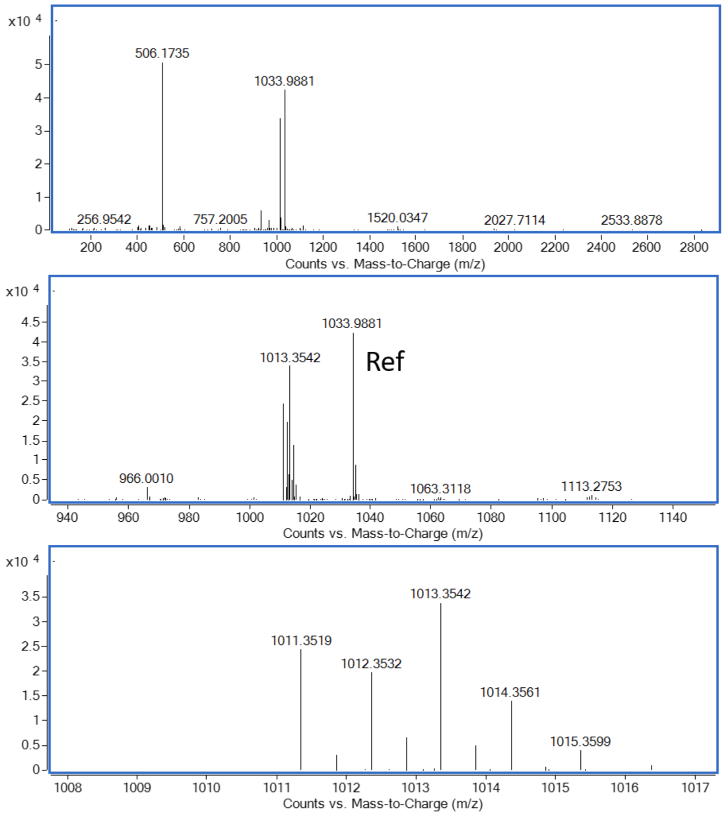

Figure S56. Mass spectrum of EuL4. m/z (ESI-MS$^-$) 1013.3542 ([M]$^-$ calculated: 1013.3533).

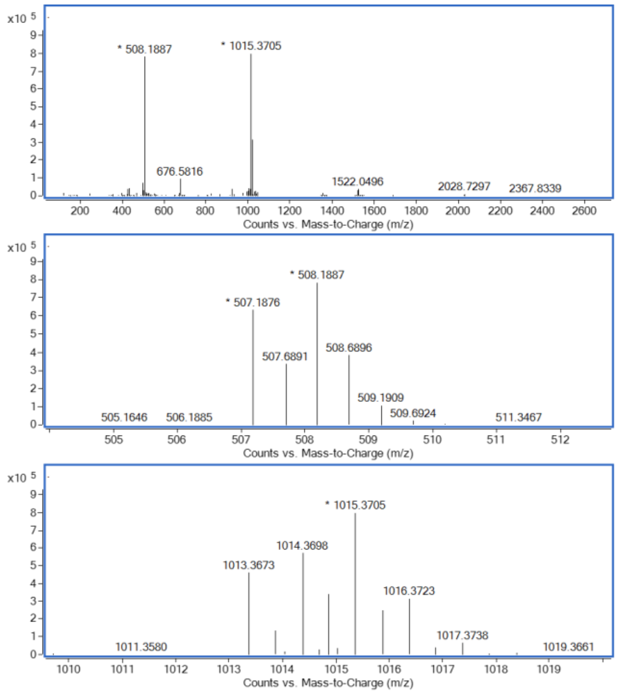

Figure S57. Mass spectrum of EuL5. m/z (ESI-MS$^+$) 1015.3705 ([M+2H]$^+$ calculated: 1015.3689).

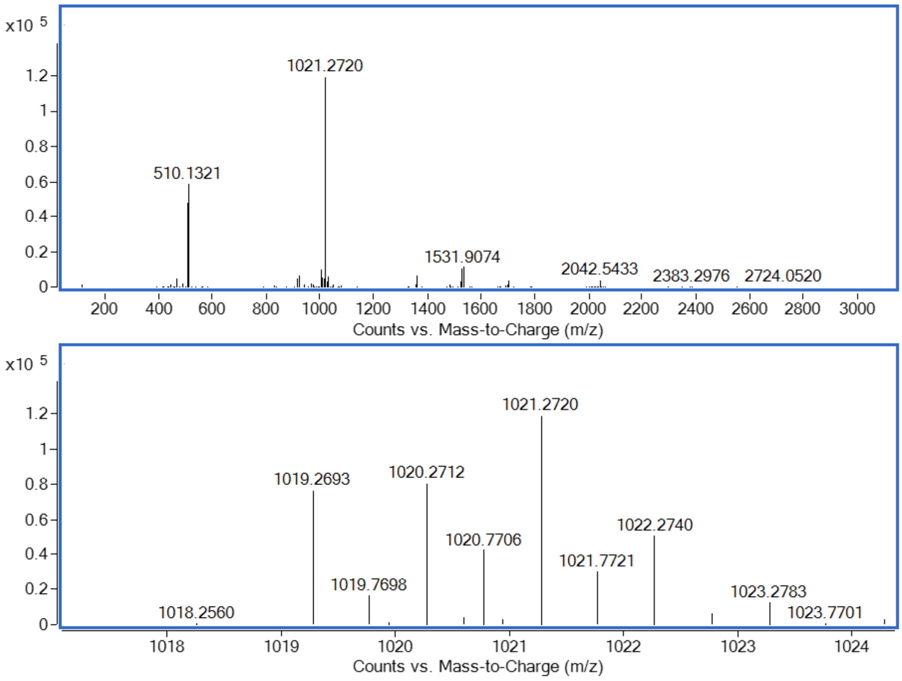

Figure S58. Mass spectrum of EuL6. m/z (ESI-MS$^-$) 1021.2720 ([M]$^-$ calculated: 1021.2703).

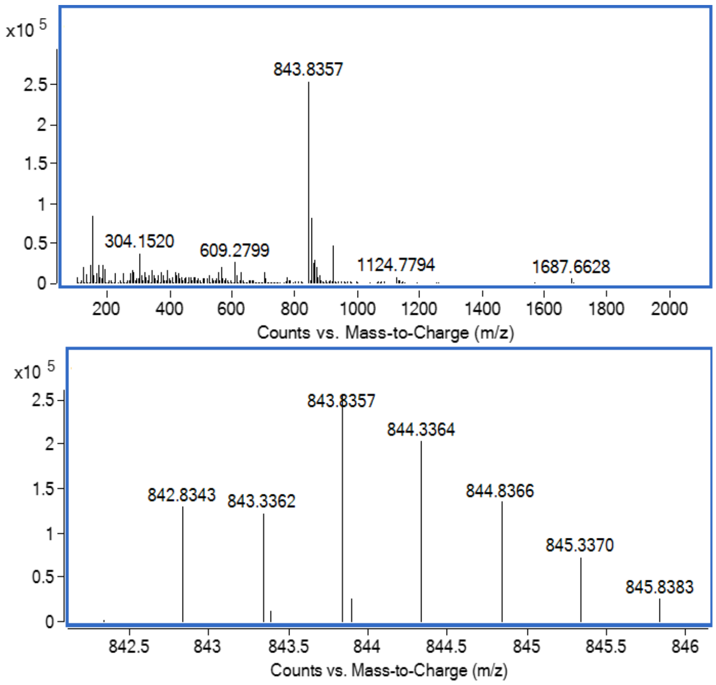

Figure S59. Mass spectrum of EuL7. m/z 1/2(ESI-MS$^+$) 843.8357 (1/2[M+3H]$^{2+}$ calculated: 843.8351).

5. HPLC Traces

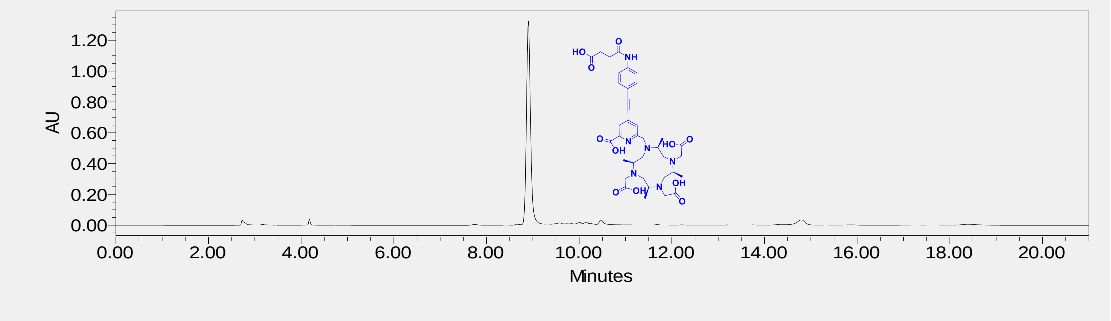

Figure S60. HPLC trace of L2 (UV 350 nm).

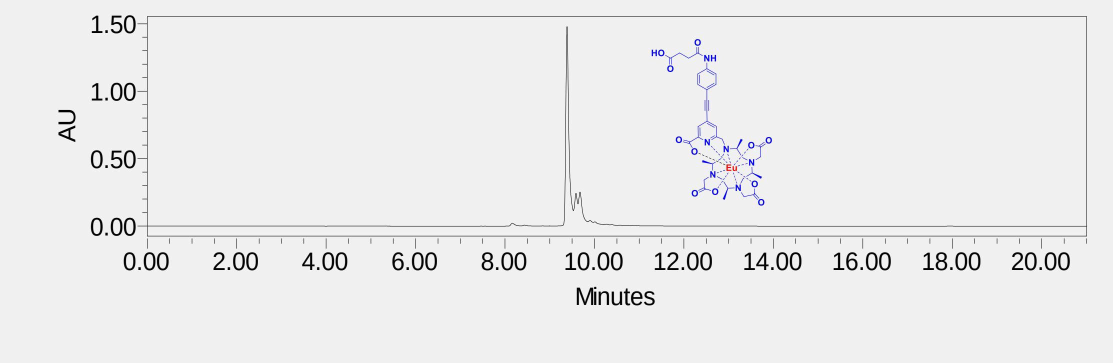

Figure S61. HPLC trace of EuL2 (before purification) (UV 350 nm).

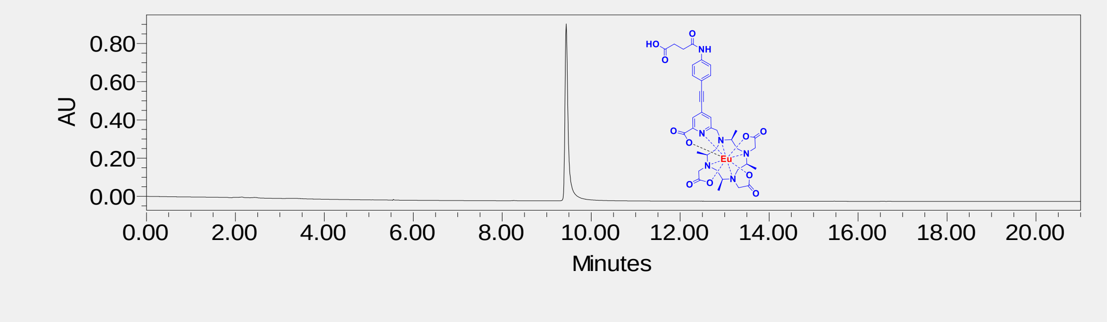

Figure S62. HPLC trace of EuL2 (after purification) (UV 350 nm).

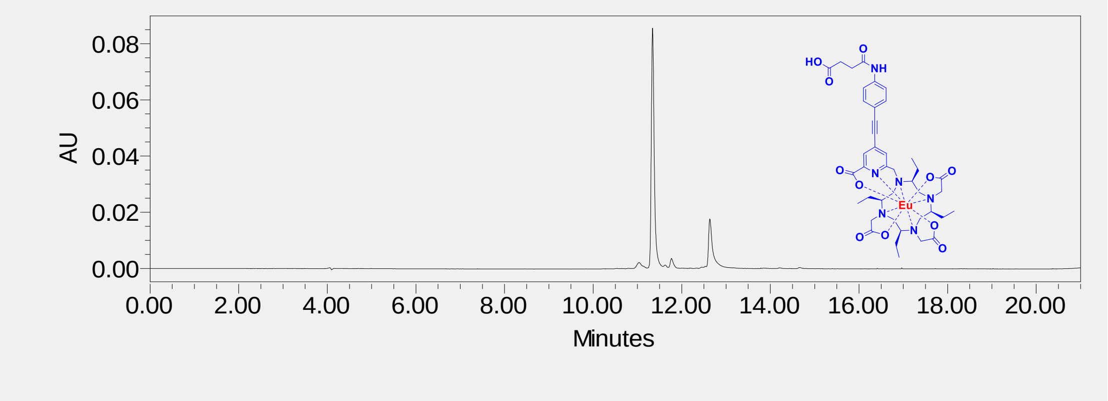

Figure S63. HPLC trace of EuL3 (before purification) (UV 350 nm).

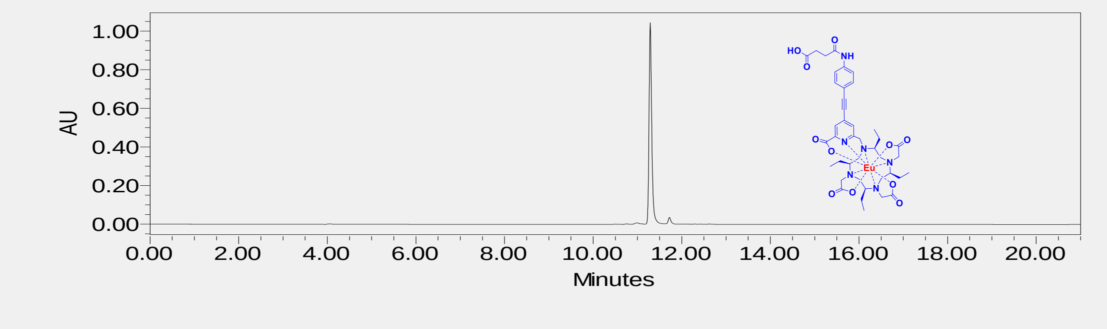

Figure S64. HPLC trace of (SAP)EuL3 (the 1st isomer) (UV 350 nm).

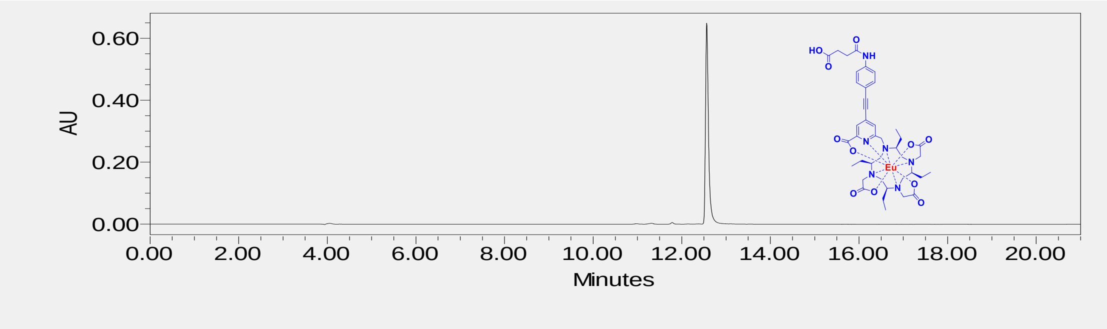

Figure S65. HPLC trace of (TSAP)EuL3 (the 2nd isomer) (UV 350 nm).

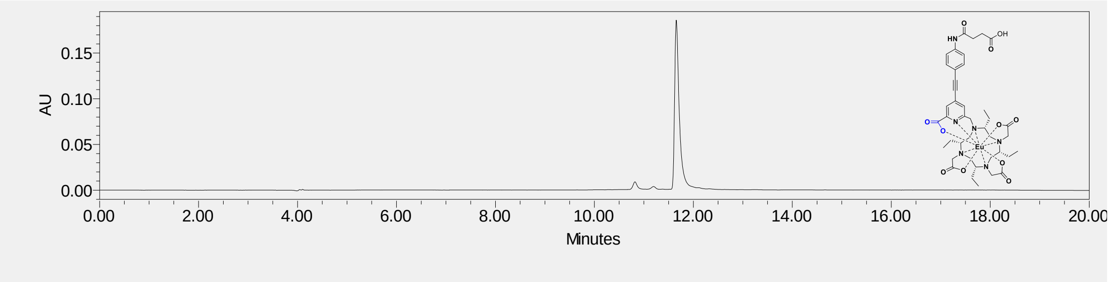

Figure S66. HPLC trace of (*R*)(SAP)EuL3 (UV 350 nm).

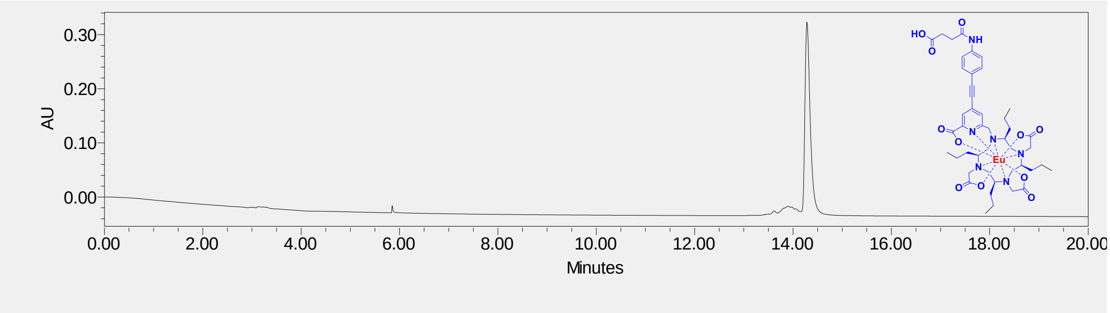

Figure S67. HPLC trace of EuL4 (UV 350 nm).

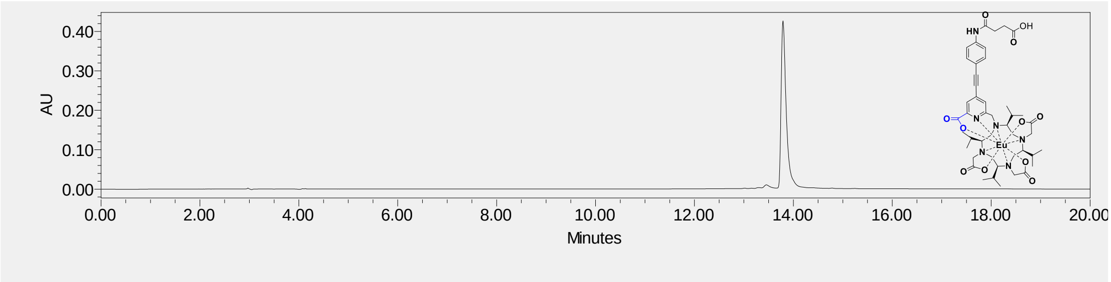

Figure S68. HPLC trace of EuL5 (UV 350 nm).

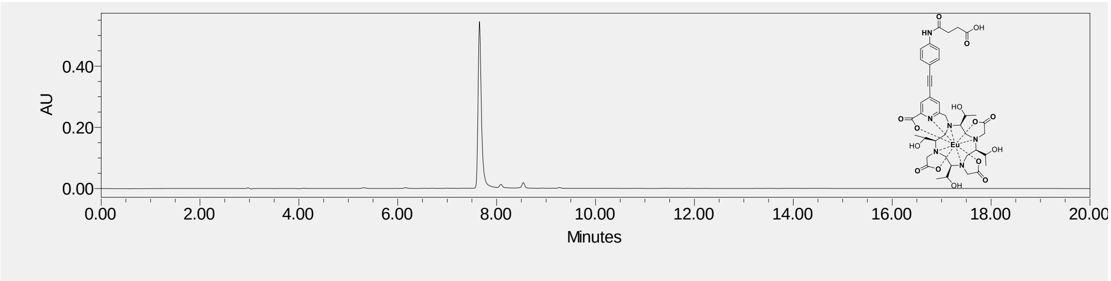

Figure S69. HPLC trace of EuL6 (UV 350 nm).

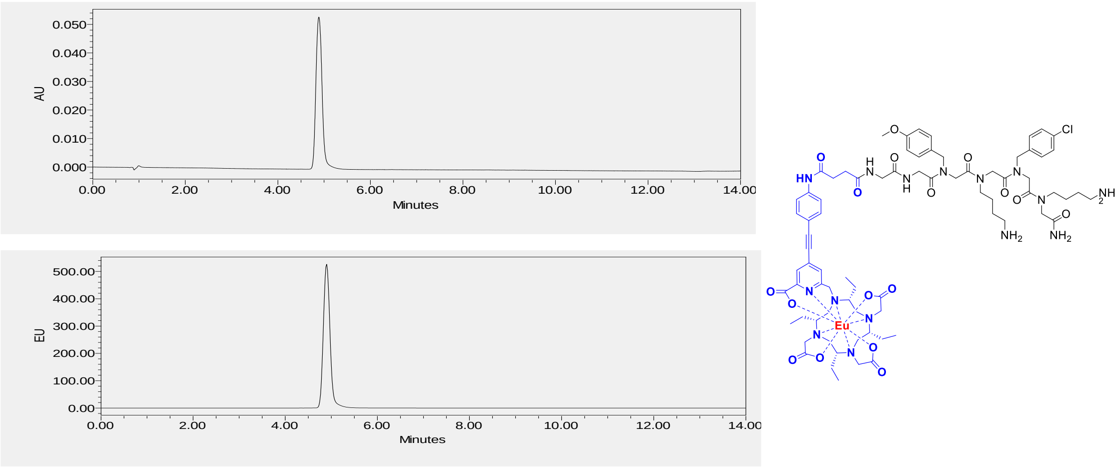

Figure S70. HPLC trace of EuL7 (UV 350 nm and fluorescence 615 nm).

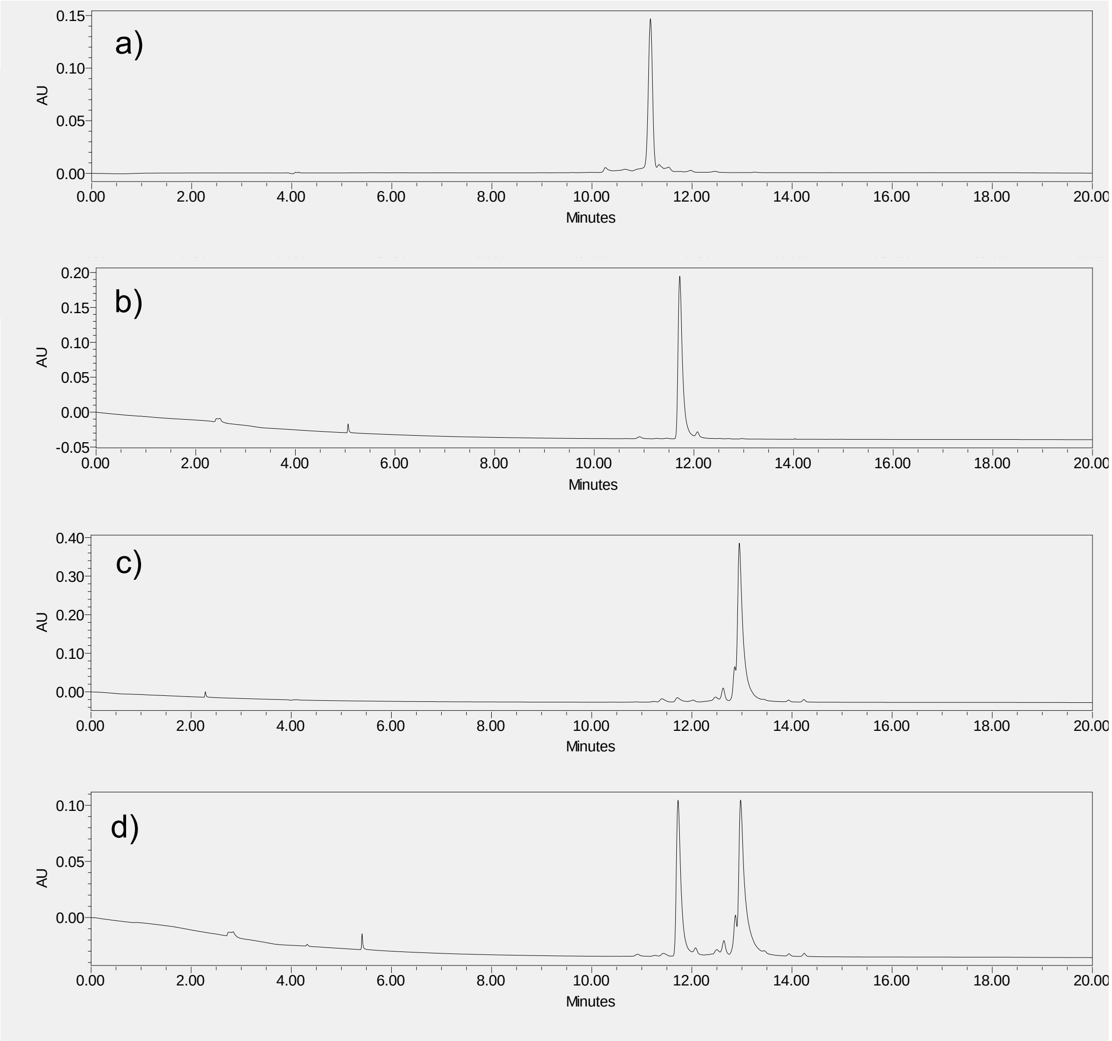

Figure S71. HPLC trace of a) pure ligand L3; b) (SAP)EuL3 after 3 days dissolved in water; c) (TSAP)EuL3 after 3 days dissolved in water; d) mixture of (SAP) and (TSAP)EuL3 after 3 days dissolved in water (UV 350 nm).

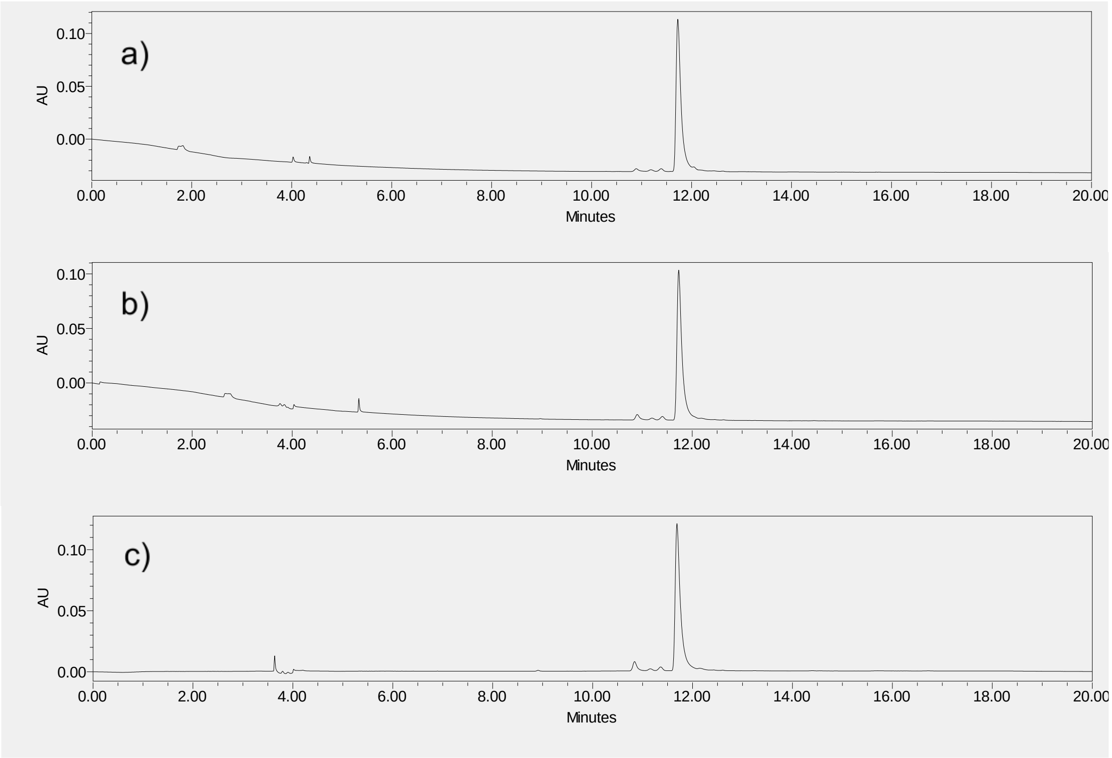

Figure S72. HPLC trace of a) (SAP)EuL3 after 10 mins dissolved in 95% TFA; b) (SAP)EuL3 after 1 hr dissolved in 95% TFA; c) (SAP)EuL3 after 2 hrs dissolved in 95% TFA (UV 350 nm).

6. **Single Crystal X-ray Diffraction**

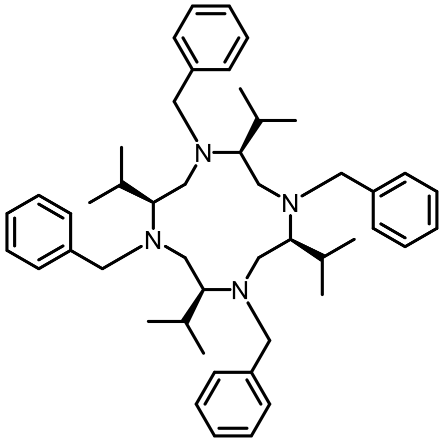

Figure S73. Structure of Compound 13.

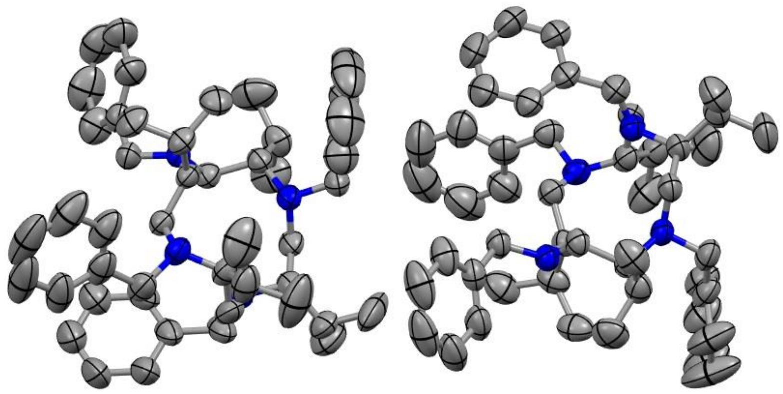

Figure S74. Crystal structures of Compound 13. Hydrogen atoms are omitted for clarity. Thermal ellipsoids are drawn at 50% probability. Color code: blue (N); grey (C).

7. NMR Spectra

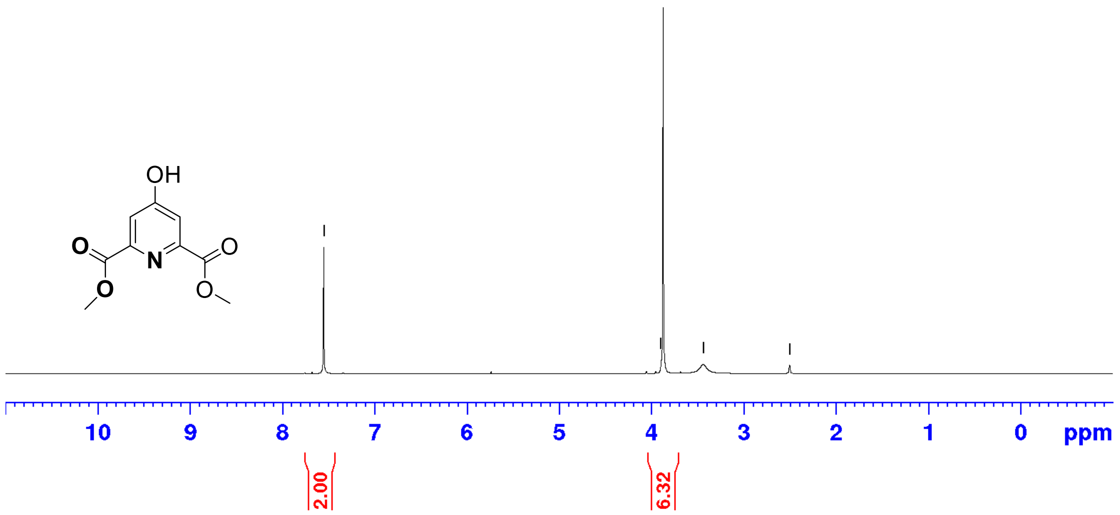

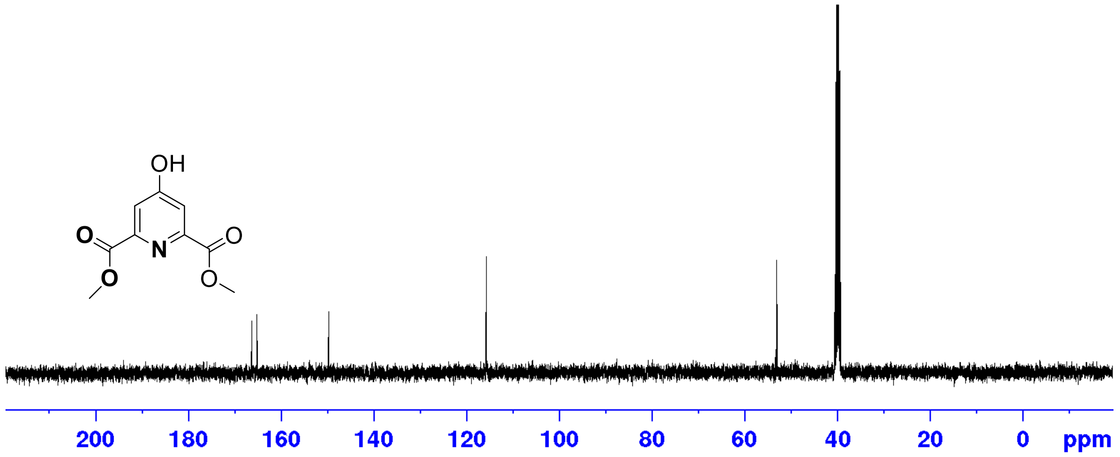

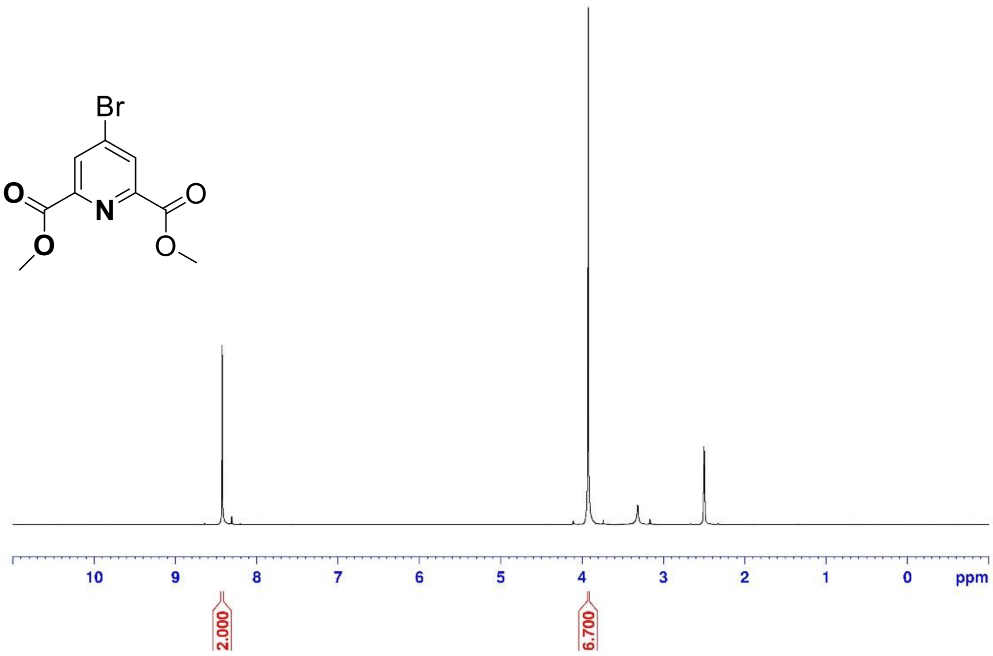

Br
O
N
O
O
O
10
9
8
7
6
5
4
3
2
1
0
ppm
2.000
6.700

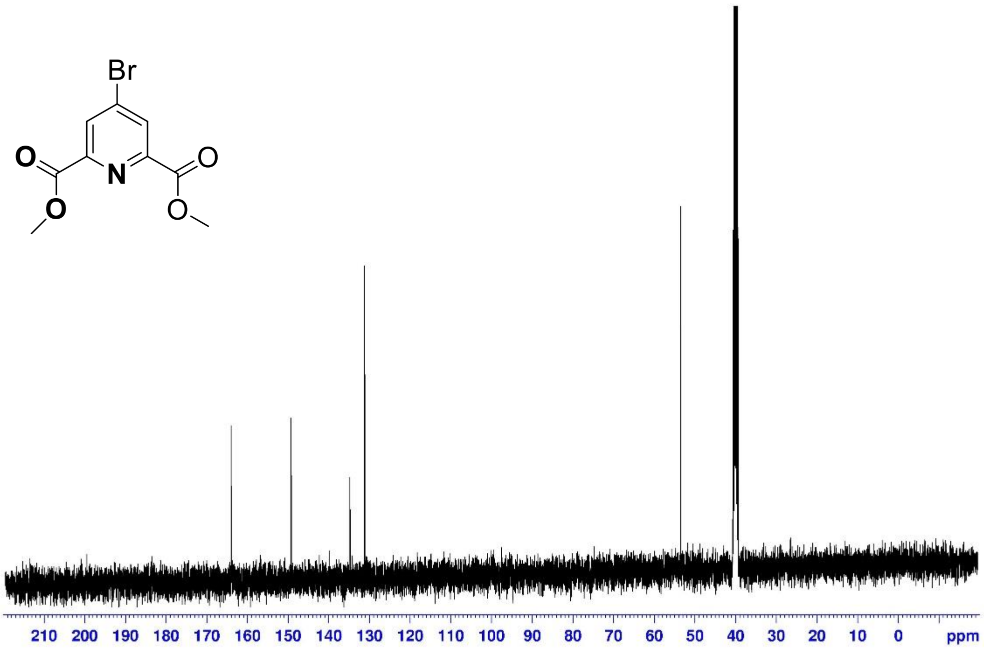

Br
O
N
O
O
O
210 200 190 180 170 160 150 140 130 120 110 100 90 80 70 60 50 40 30 20 10 0 ppm

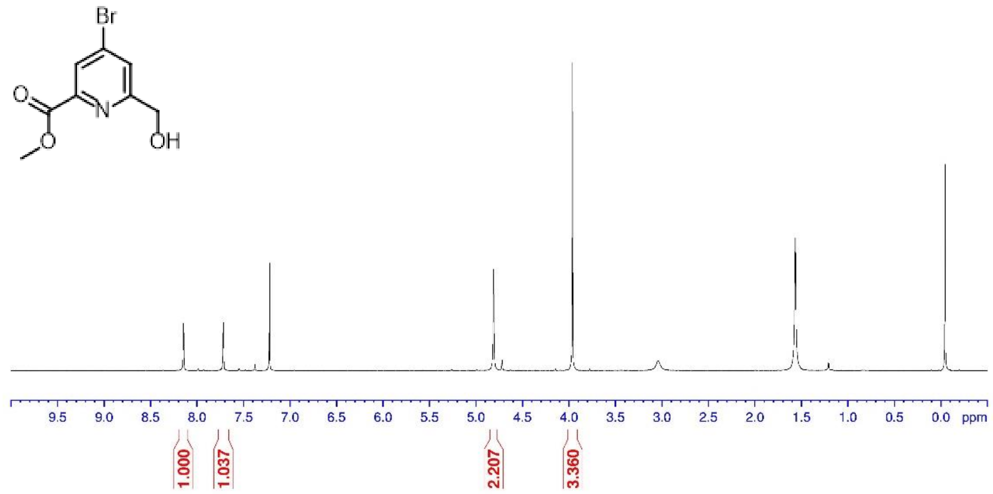
Br
O
N
O
OH
9.5 9.0 8.5 8.0 7.5 7.0 6.5 6.0 5.5 5.0 4.5 4.0 3.5 3.0 2.5 2.0 1.5 1.0 0.5 0.0 ppm
1.000
1.037
2.207
3.360

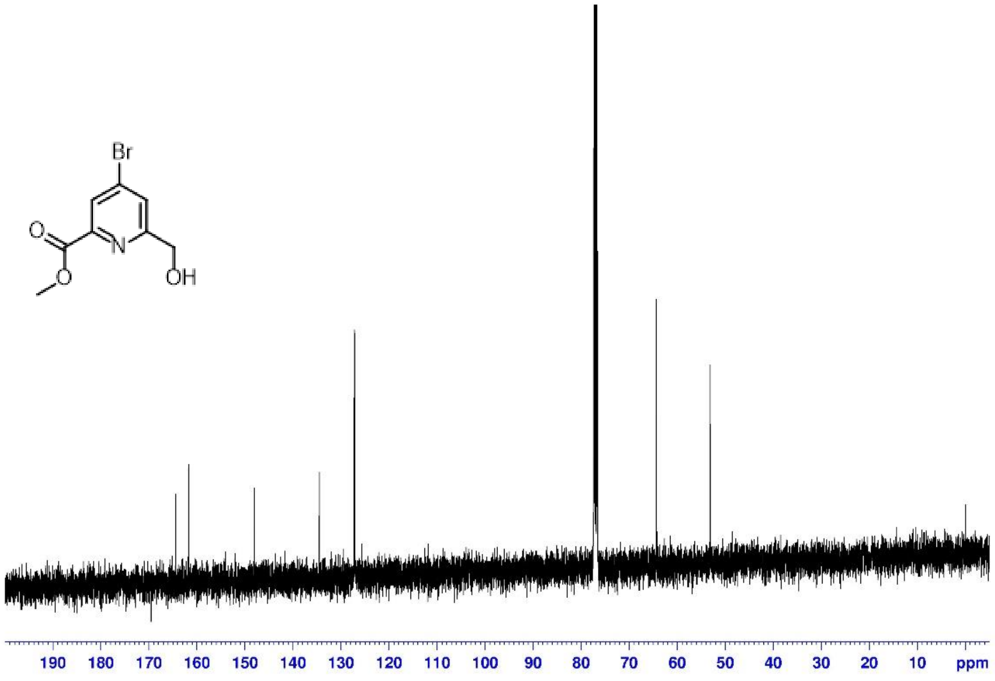
Br
O
N
O
OH
190 180 170 160 150 140 130 120 110 100 90 80 70 60 50 40 30 20 10 ppm

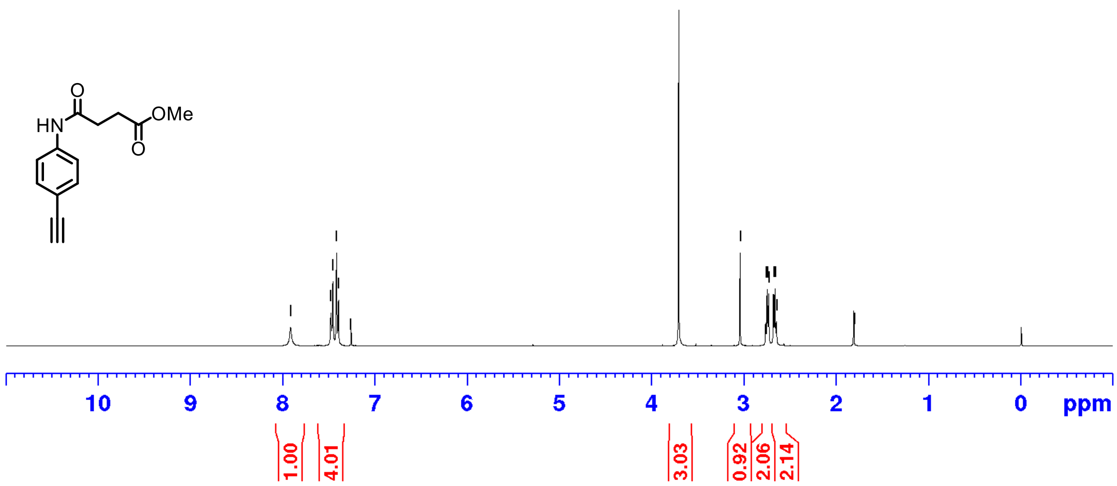

HN
O
OMe
O
10
9
8
7
6
5
4
3
2
1
0
ppm
1.00
4.01
3.03
0.92
2.06
2.14

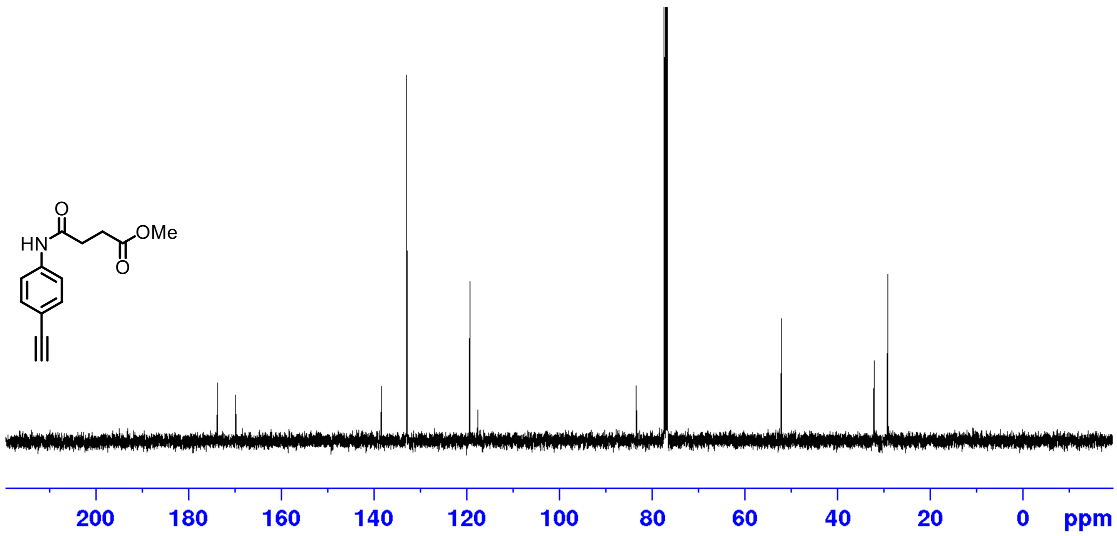

HN
O
OMe
O
200
180
160
140
120
100
80
60
40
20
0
ppm

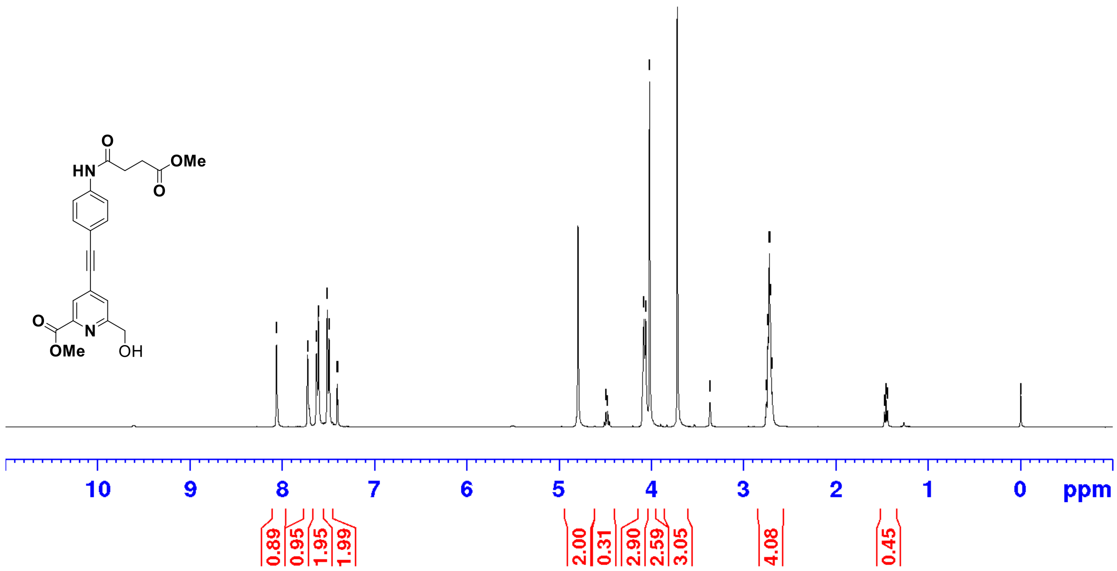

HN
O
OMe
O
O
N
OMe
OH
10
9
8
7
6
5
4
3
2
1
0
ppm
0.89
0.95
1.95
1.99
2.00
0.31
2.90
2.59
3.05
4.08
0.45

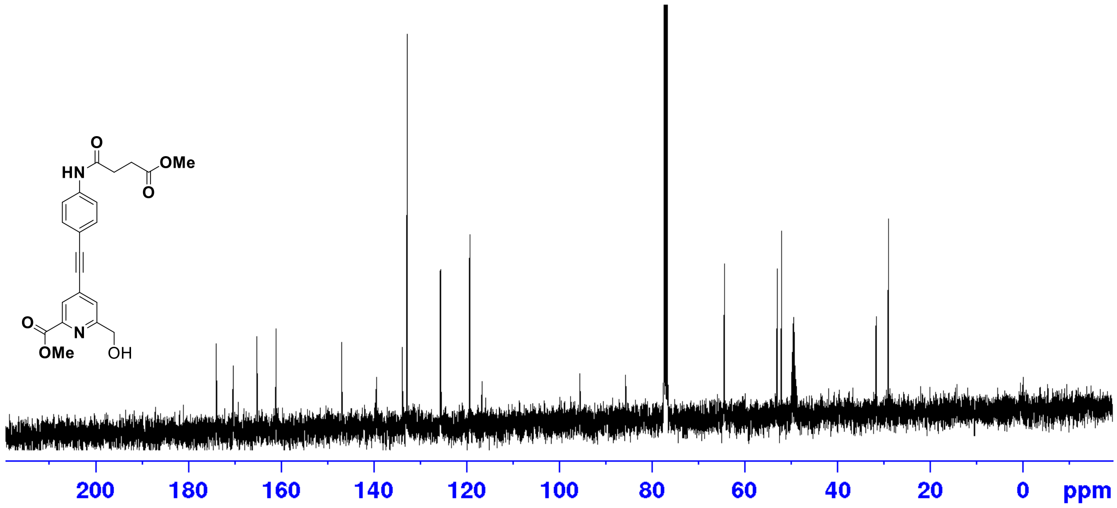
HN
O
OMe
O
N
O
OMe
OH
200 180 160 140 120 100 80 60 40 20 0 ppm

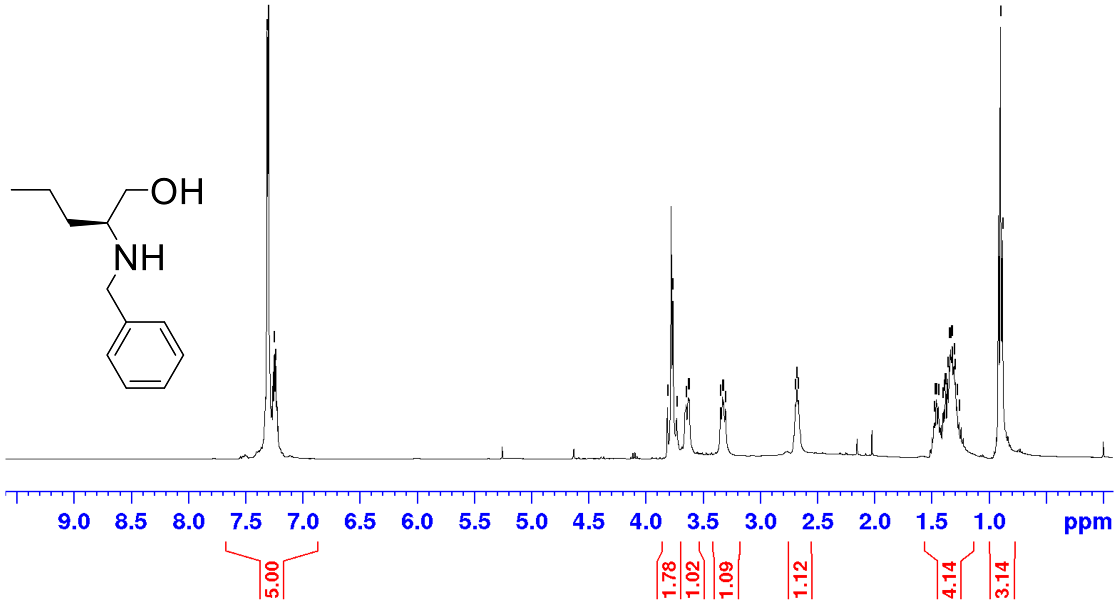
OH
NH
9.0 8.5 8.0 7.5 7.0 6.5 6.0 5.5 5.0 4.5 4.0 3.5 3.0 2.5 2.0 1.5 1.0 ppm
5.00
1.78
1.02
1.09
1.12
4.14
3.14

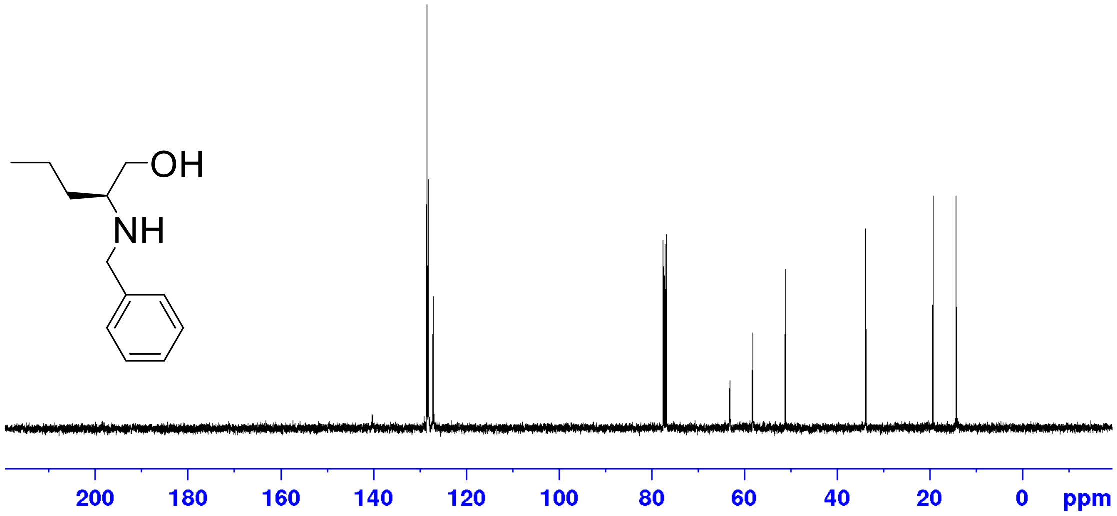
OH
NH
200 180 160 140 120 100 80 60 40 20 0 ppm

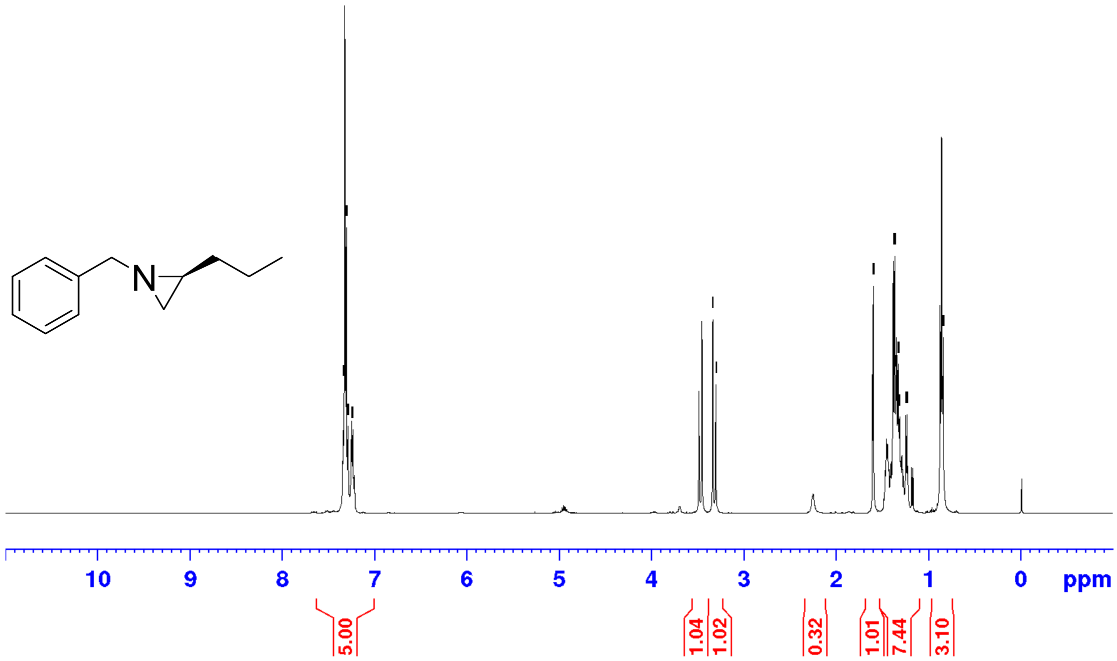
N
10
9
8
7
6
5
4
3
2
1
0
ppm
5.00
1.04
1.02
0.32
1.01
7.44
3.10

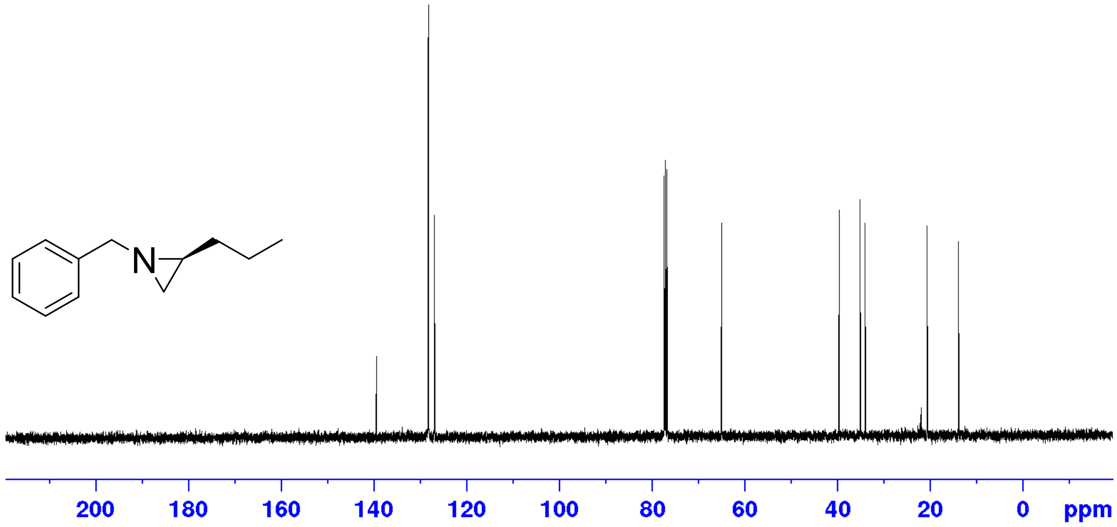
N
200
180
160
140
120
100
80
60
40
20
0
ppm

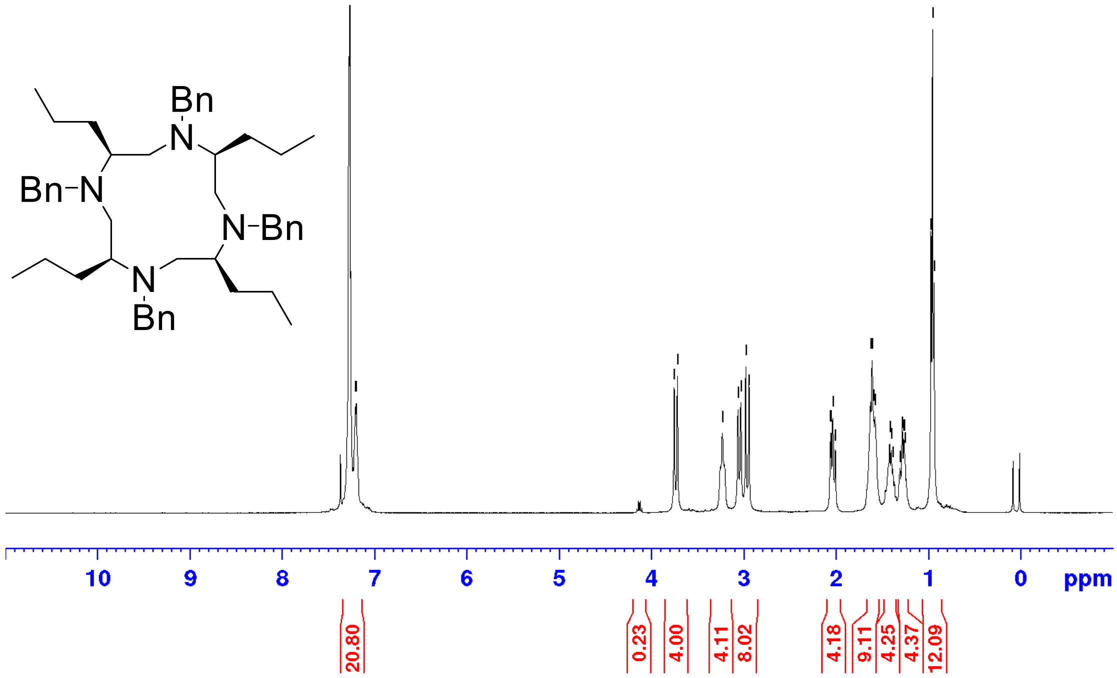
Bn
N
Bn–N
N–Bn
N
Bn
10
9
8
7
6
5
4
3
2
1
0
ppm
20.80
0.23
4.00
4.11
8.02
4.18
9.11
4.25
4.37
12.09

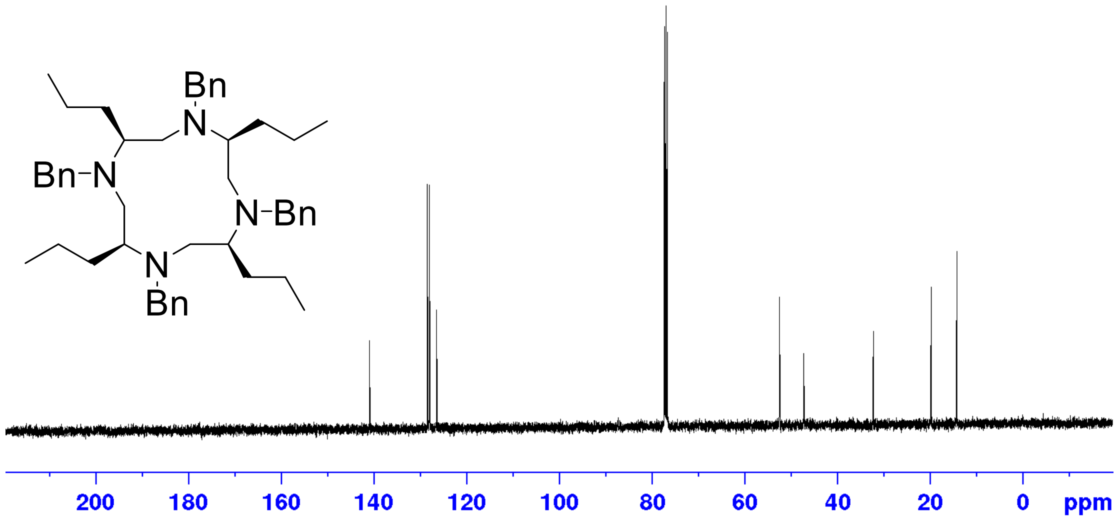
Bn
N
Bn–N
N–Bn
N
Bn
200
180
160
140
120
100
80
60
40
20
0
ppm

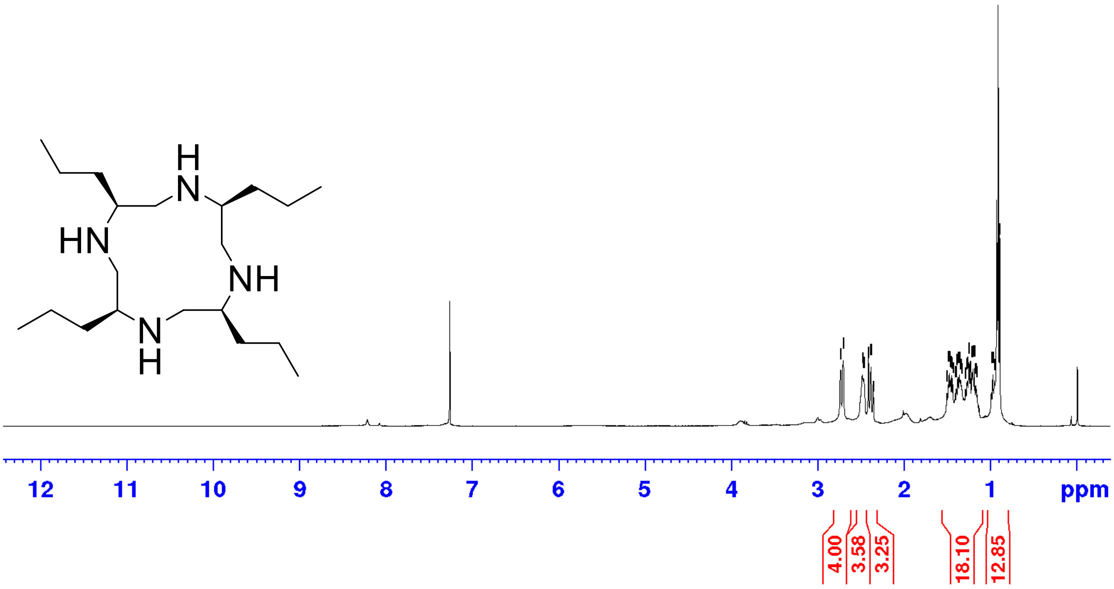
H
N
HN
NH
N
H
12 11 10 9 8 7 6 5 4 3 2 1 ppm
4.00 3.58 3.25 18.10 12.85

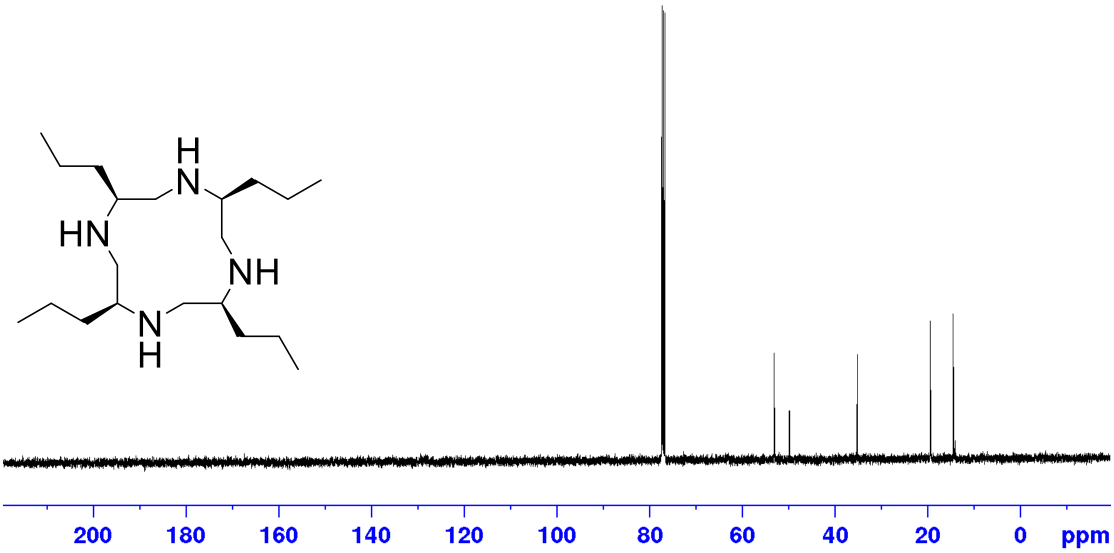
H
N
HN
NH
N
H
200 180 160 140 120 100 80 60 40 20 0 ppm

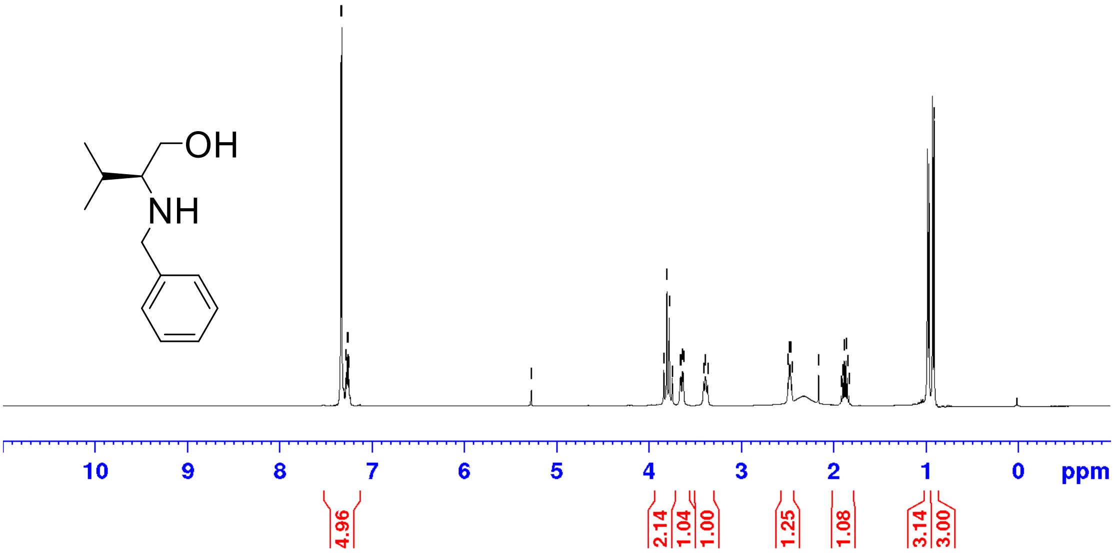
OH
NH
10 9 8 7 6 5 4 3 2 1 0 ppm
4.96 2.14 1.04 1.00 1.25 1.08 3.14 3.00

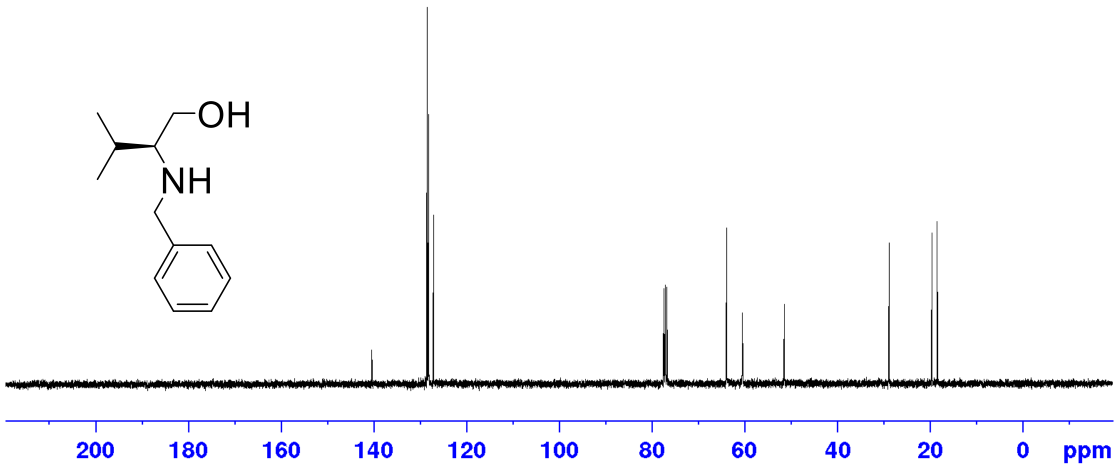
OH
NH
200
180
160
140
120
100
80
60
40
20
0
ppm

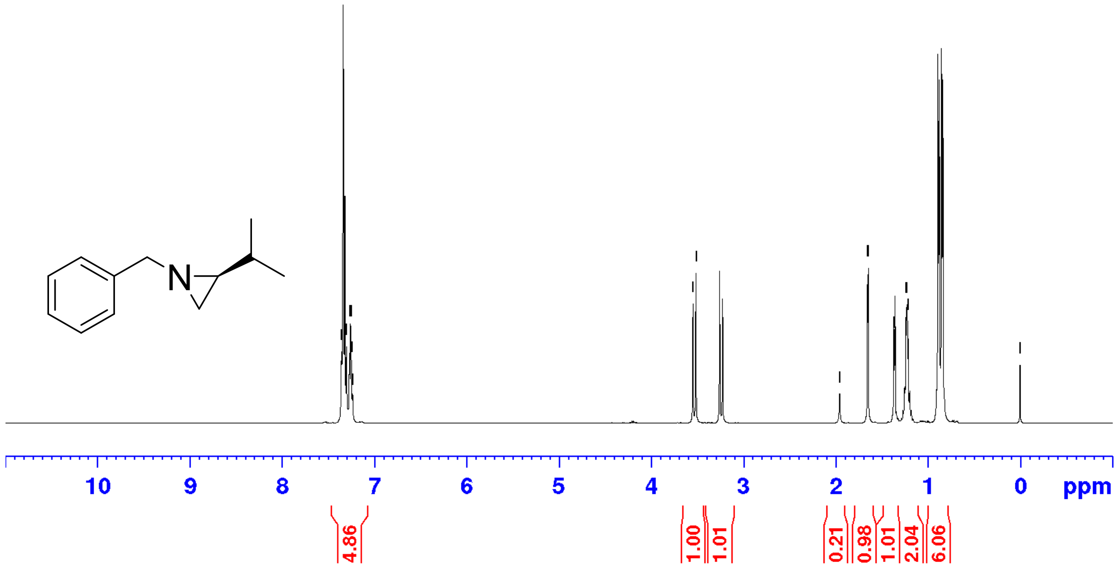
N
10
9
8
7
6
5
4
3
2
1
0
ppm
4.86
1.00
1.01
0.21
0.98
1.01
2.04
6.06

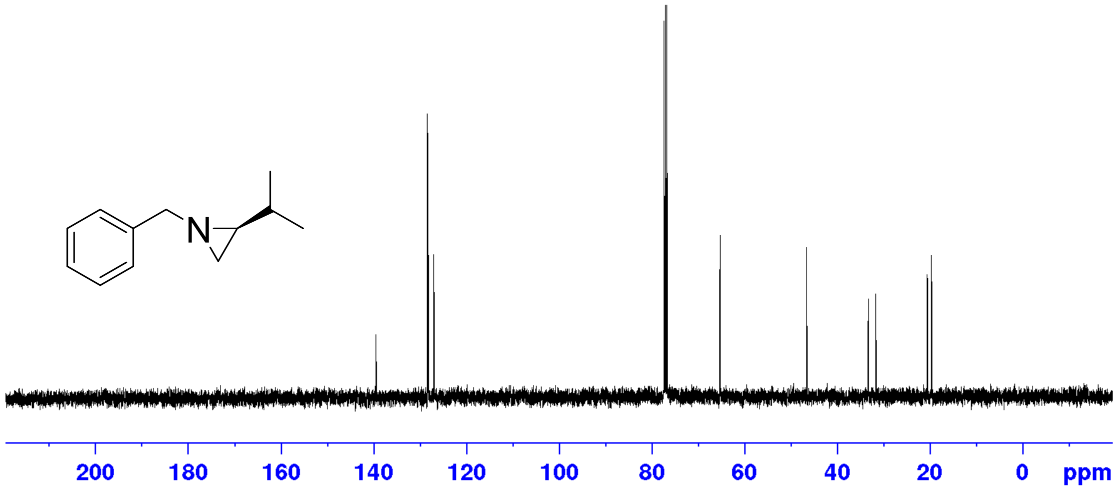
N
200
180
160
140
120
100
80
60
40
20
0
ppm

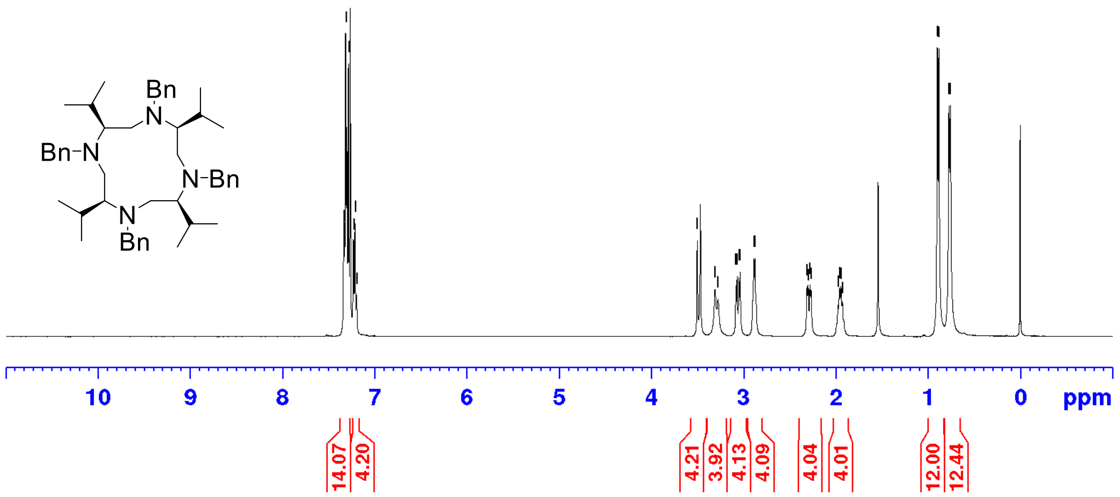

Bn
N
Bn–N
N–Bn
N
Bn
10
9
8
7
6
5
4
3
2
1
0
ppm
14.07
4.20
4.21
3.92
4.13
4.09
4.04
4.01
12.00
12.44

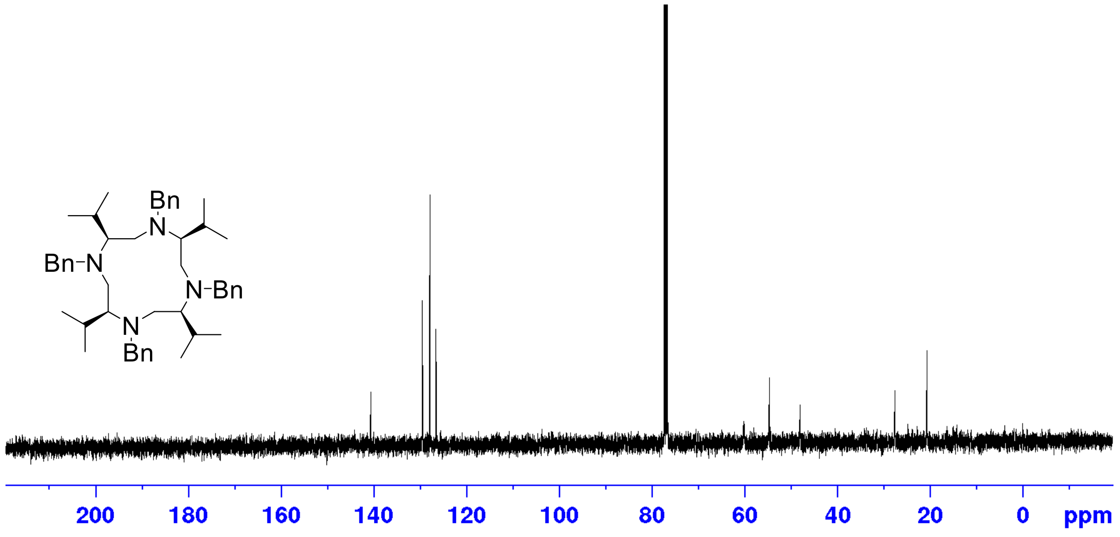

Bn
N
Bn–N
N–Bn
N
Bn
200
180
160
140
120
100
80
60
40
20
0
ppm

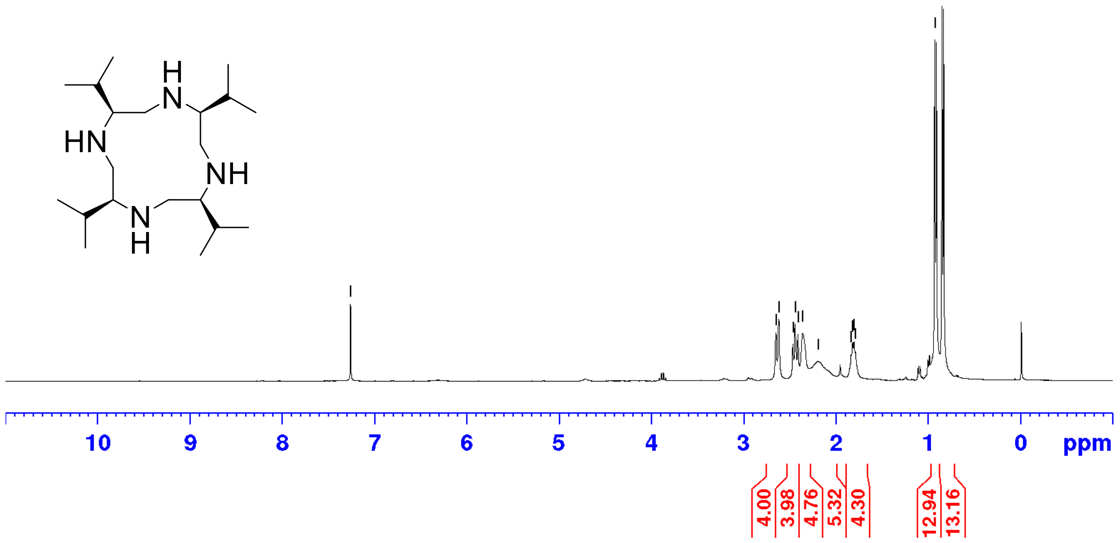

H
N
HN
NH
N
H
10
9
8
7
6
5
4
3
2
1
0
ppm
4.00
3.98
4.76
5.32
4.30
12.94
13.16

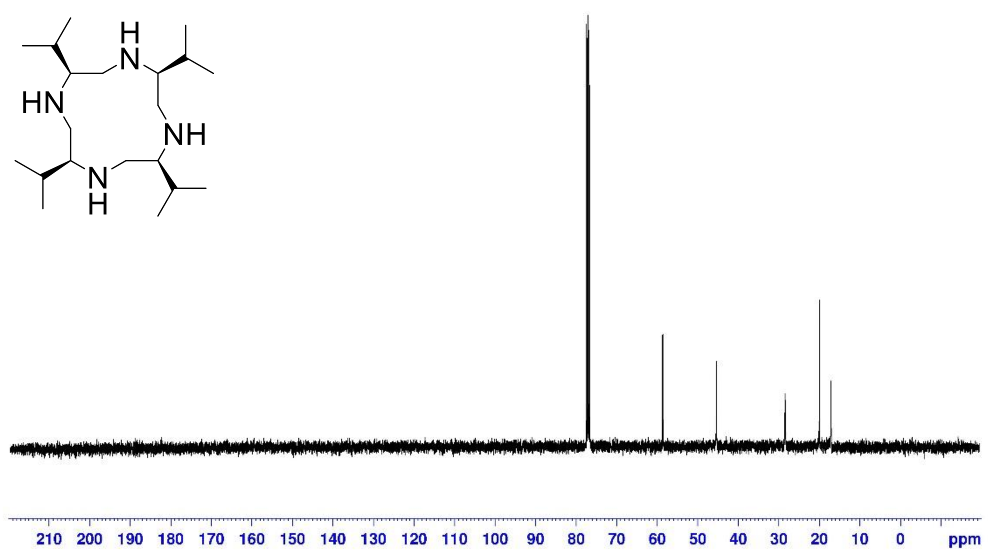

HN
NH
ppm

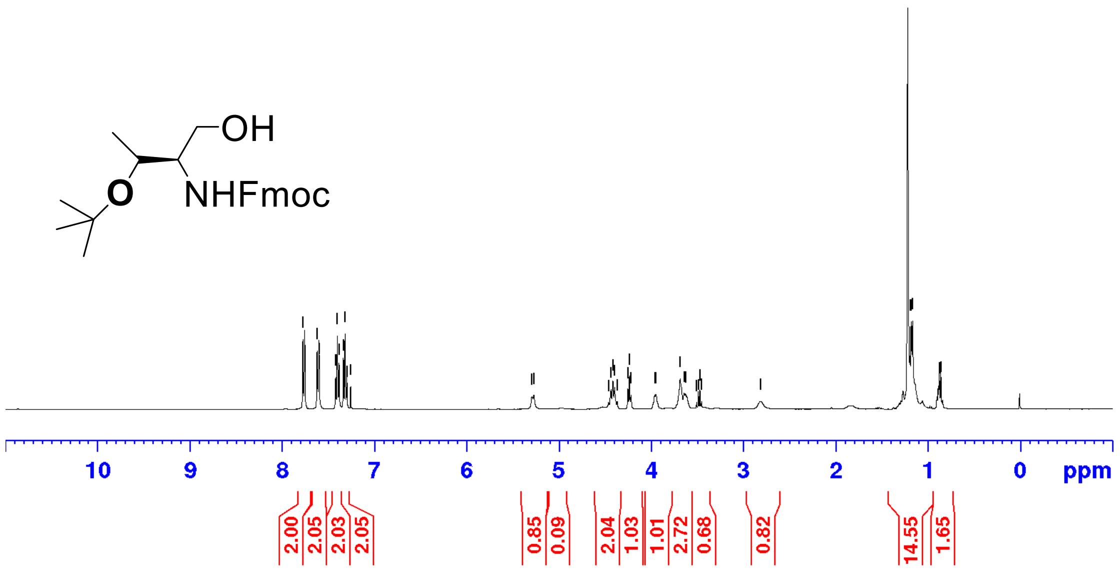

OH
O
NHFmoc
ppm
2.00
2.05
2.03
2.05
0.85
0.09
2.04
1.03
1.01
2.72
0.68
0.82
14.55
1.65

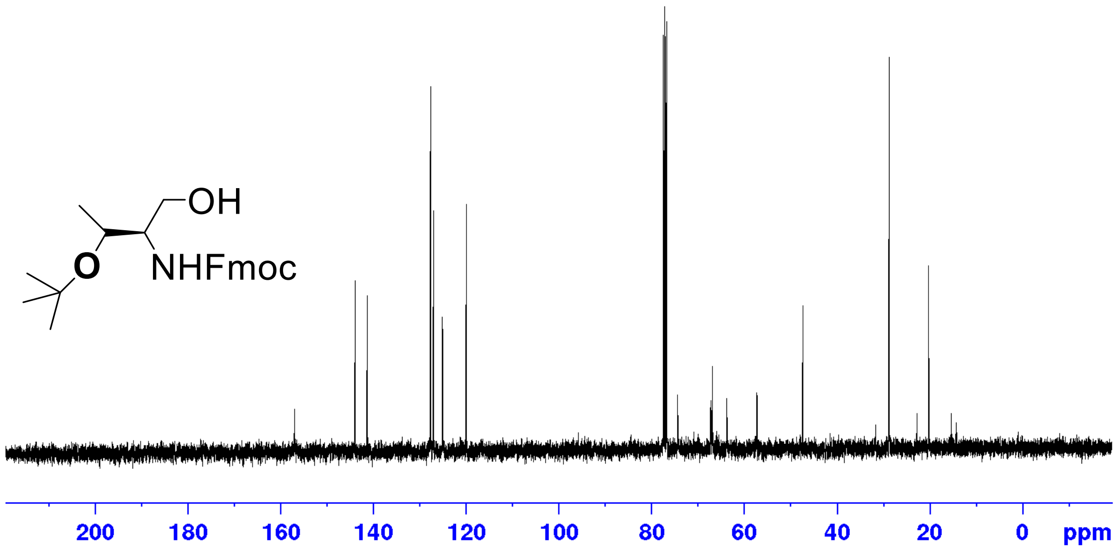

OH
O
NHFmoc
200
180
160
140
120
100
80
60
40
20
0
ppm

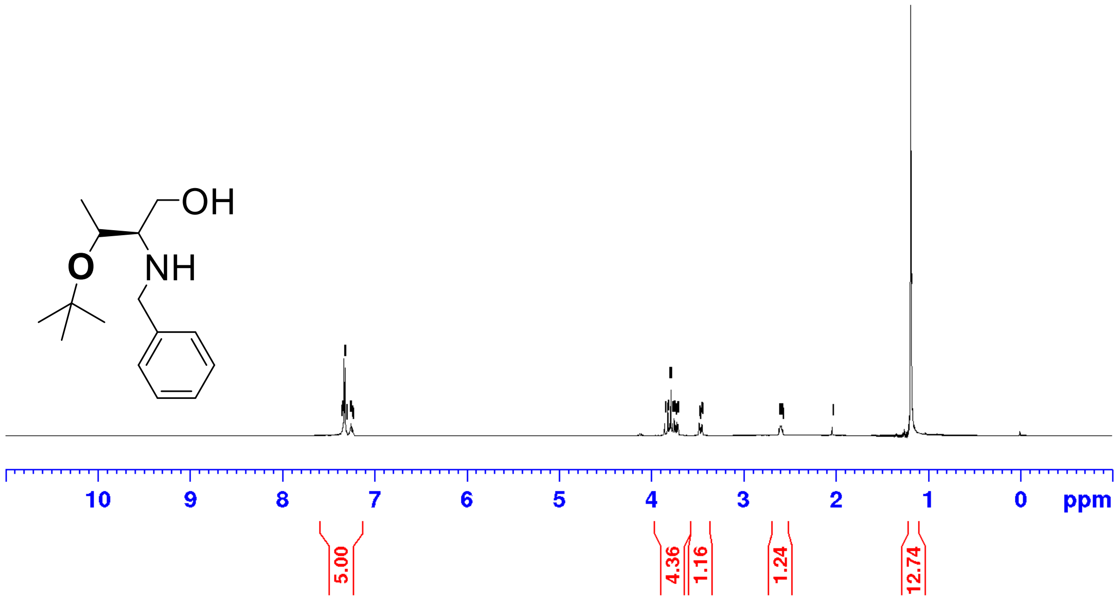

OH
O
NH
10
9
8
7
6
5
4
3
2
1
0
ppm
5.00
4.36
1.16
1.24
12.74

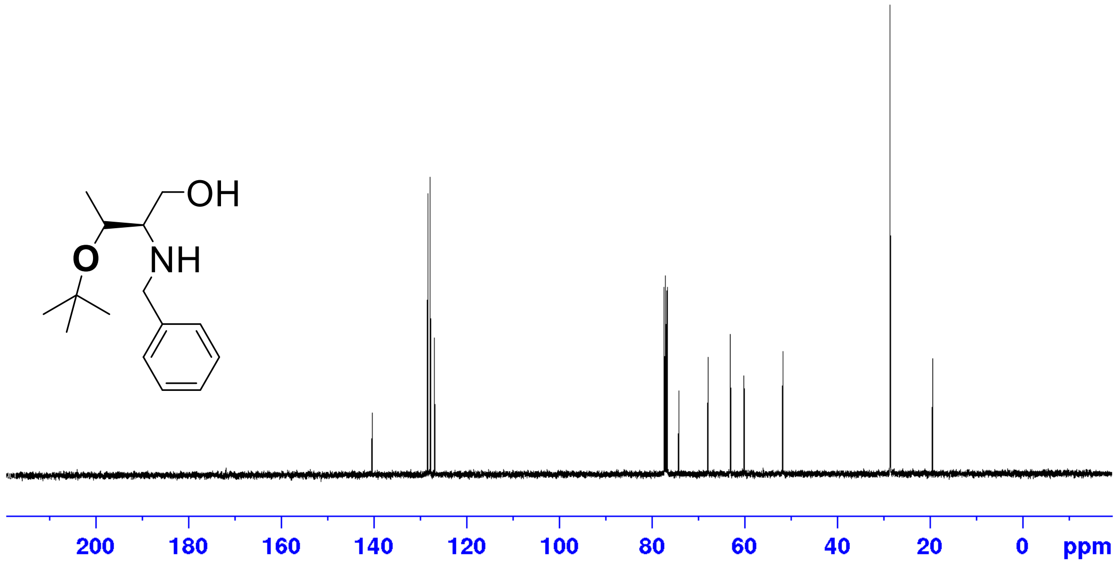

OH
O
NH
200
180
160
140
120
100
80
60
40
20
0
ppm

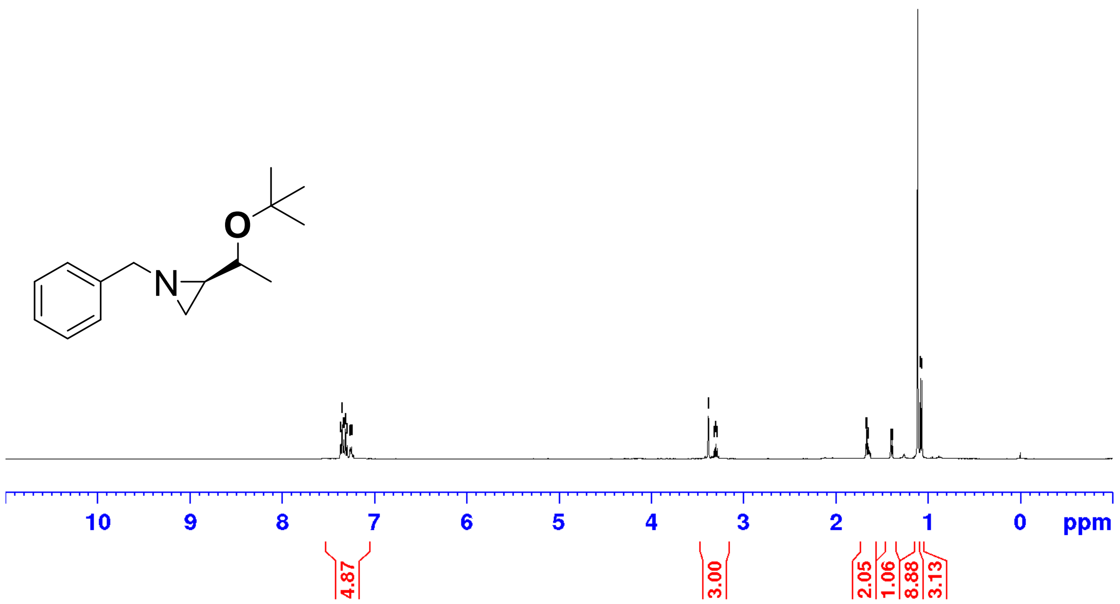
O
N
10
9
8
7
6
5
4
3
2
1
0
ppm
4.87
3.00
2.05
1.06
8.88
3.13

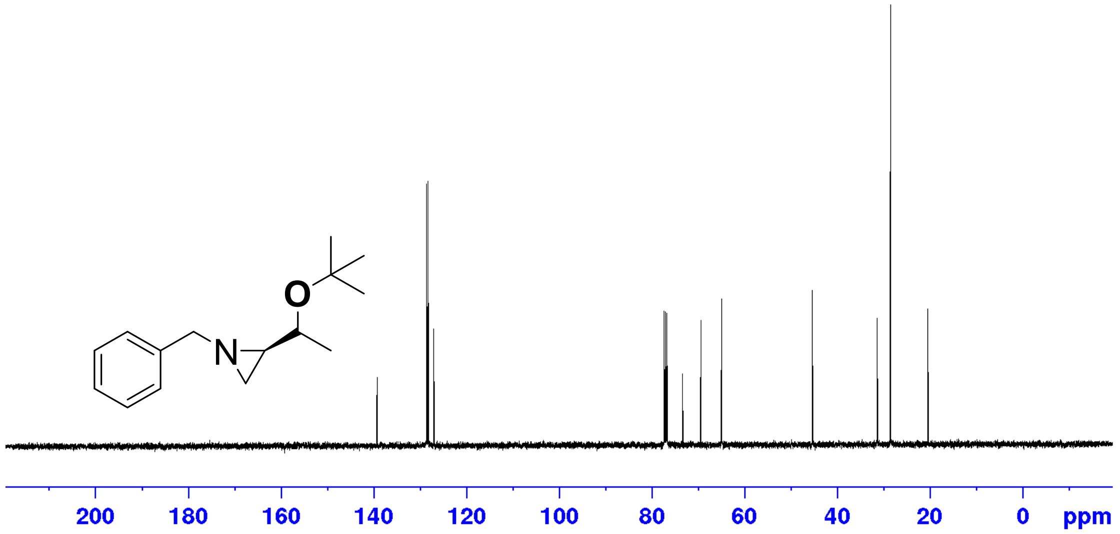
O
N
200
180
160
140
120
100
80
60
40
20
0
ppm

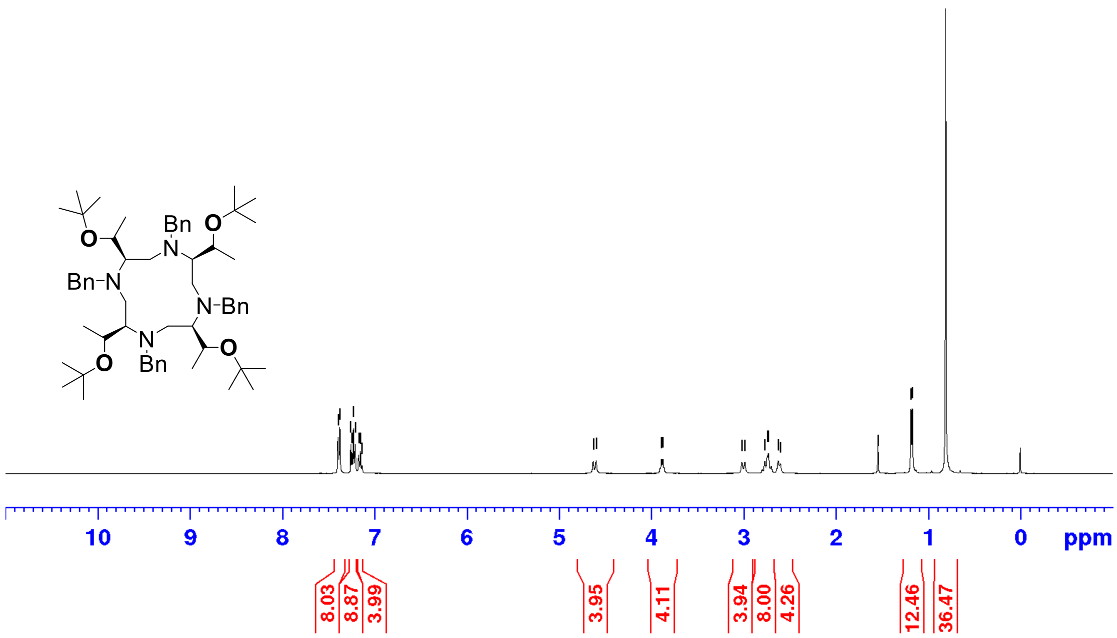
Bn
O
N
Bn-N
N-Bn
N
Bn
10 9 8 7 6 5 4 3 2 1 0 ppm
8.03
8.87
3.99
3.95
4.11
3.94
8.00
4.26
12.46
36.47

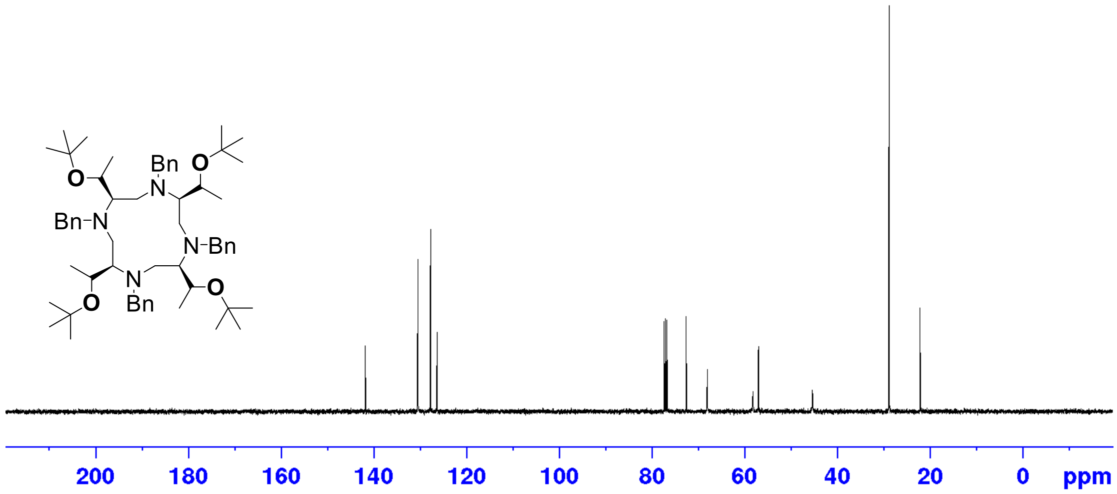
Bn
O
N
Bn-N
N-Bn
N
Bn
200 180 160 140 120 100 80 60 40 20 0 ppm

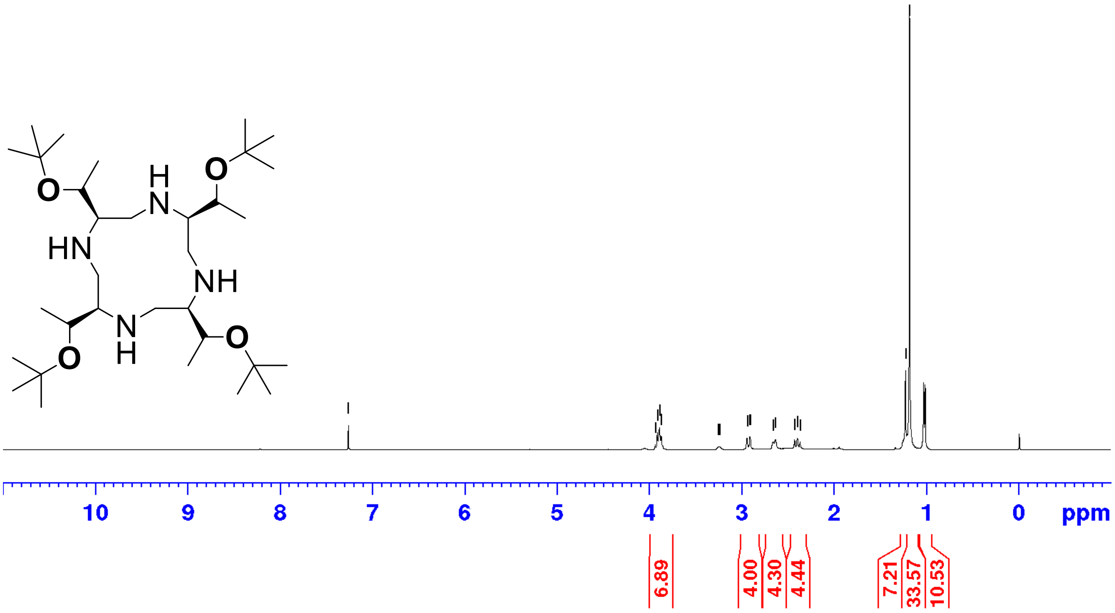
H
N
O
HN
NH
N
H
10 9 8 7 6 5 4 3 2 1 0 ppm
6.89
4.00
4.30
4.44
7.21
33.57
10.53

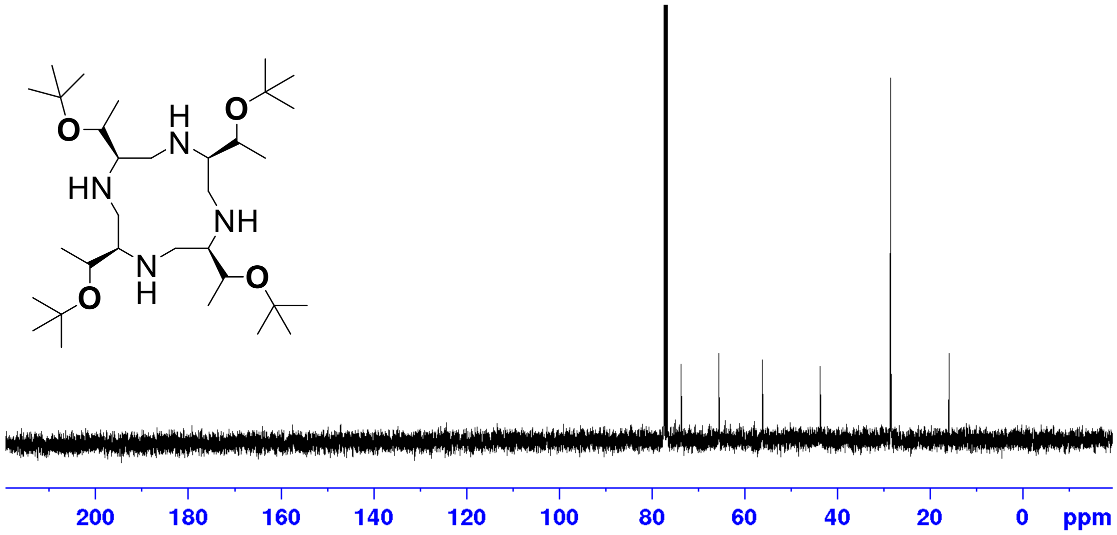
200
180
160
140
120
100
80
60
40
20
0
ppm

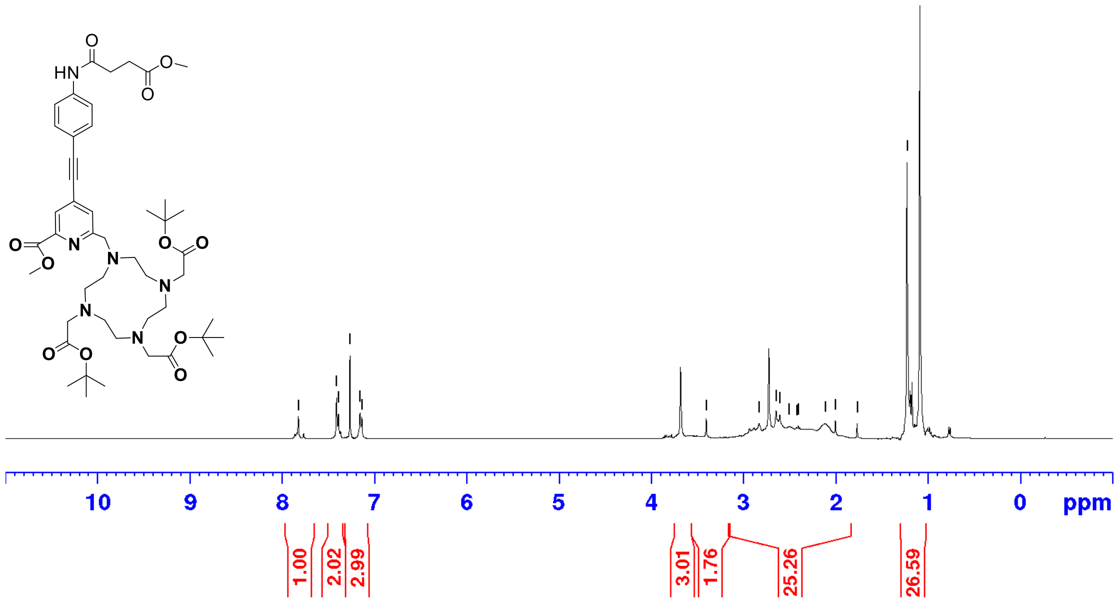
10
9
8
7
6
5
4
3
2
1
0
ppm
1.00
2.02
2.99
3.01
1.76
25.26
26.59

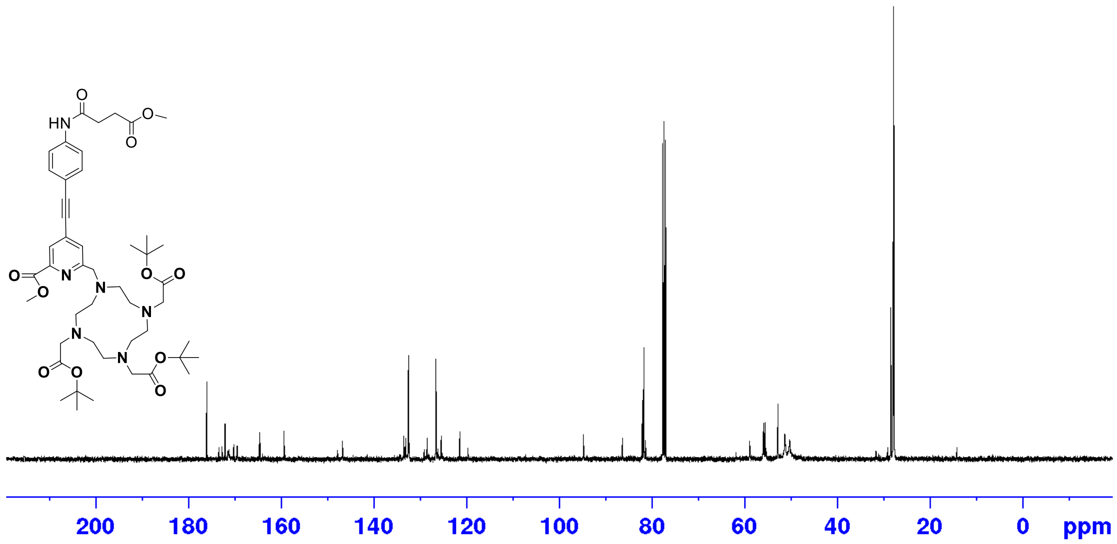
200
180
160
140
120
100
80
60
40
20
0
ppm

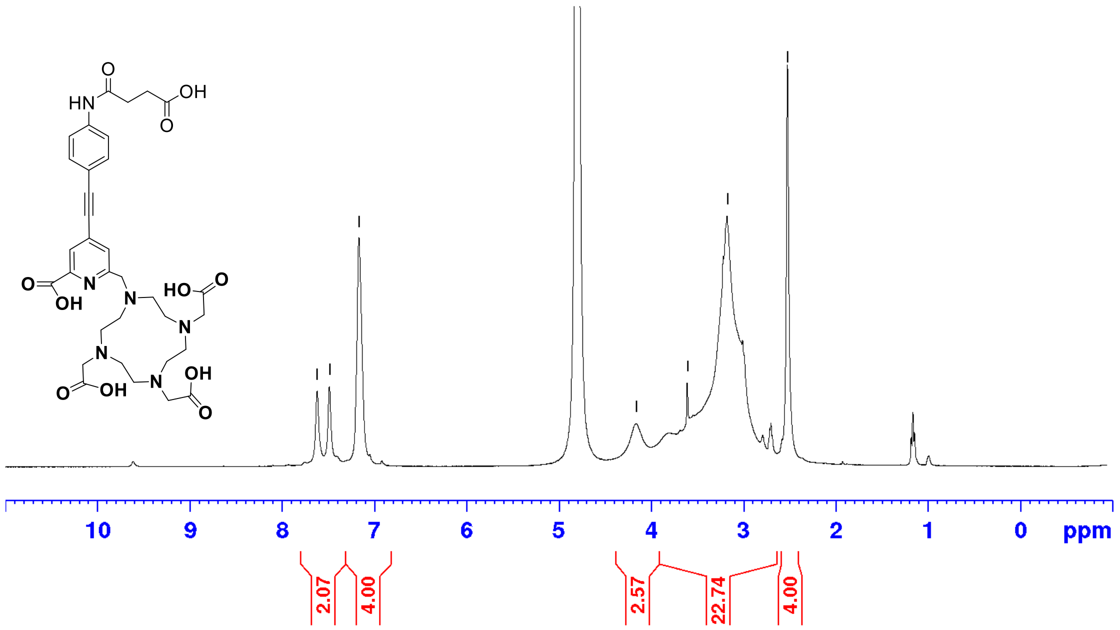
10
9
8
7
6
5
4
3
2
1
0
ppm
2.07
4.00
2.57
22.74
4.00

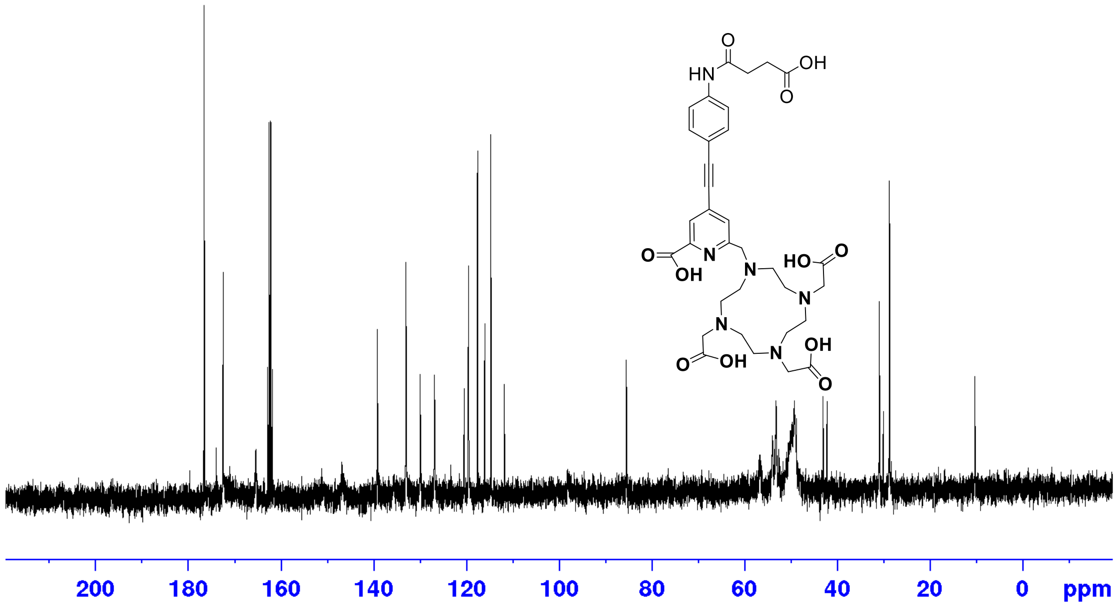
200
180
160
140
120
100
80
60
40
20
0
ppm

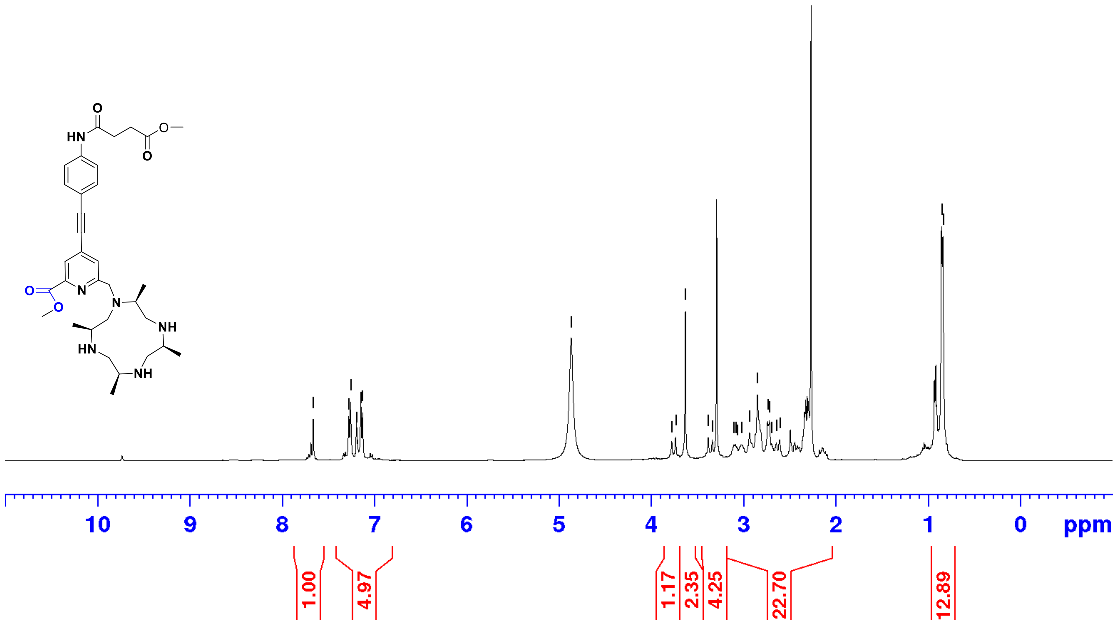
HN
O
O
O
N
N
NH
HN
NH
10
9
8
7
6
5
4
3
2
1
0
ppm
1.00
4.97
1.17
2.35
4.25
22.70
12.89

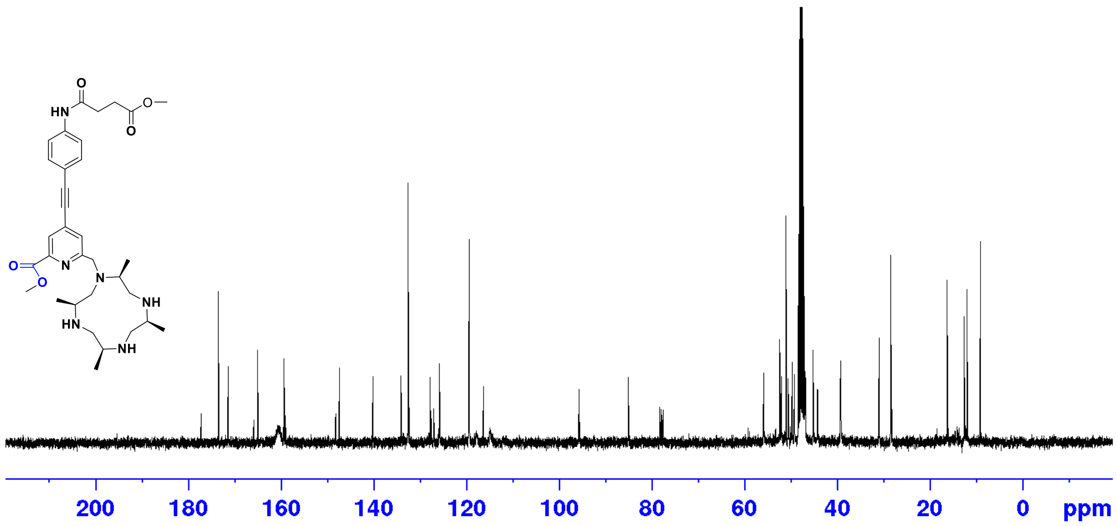
HN
O
O
O
N
N
NH
HN
NH
200
180
160
140
120
100
80
60
40
20
0
ppm

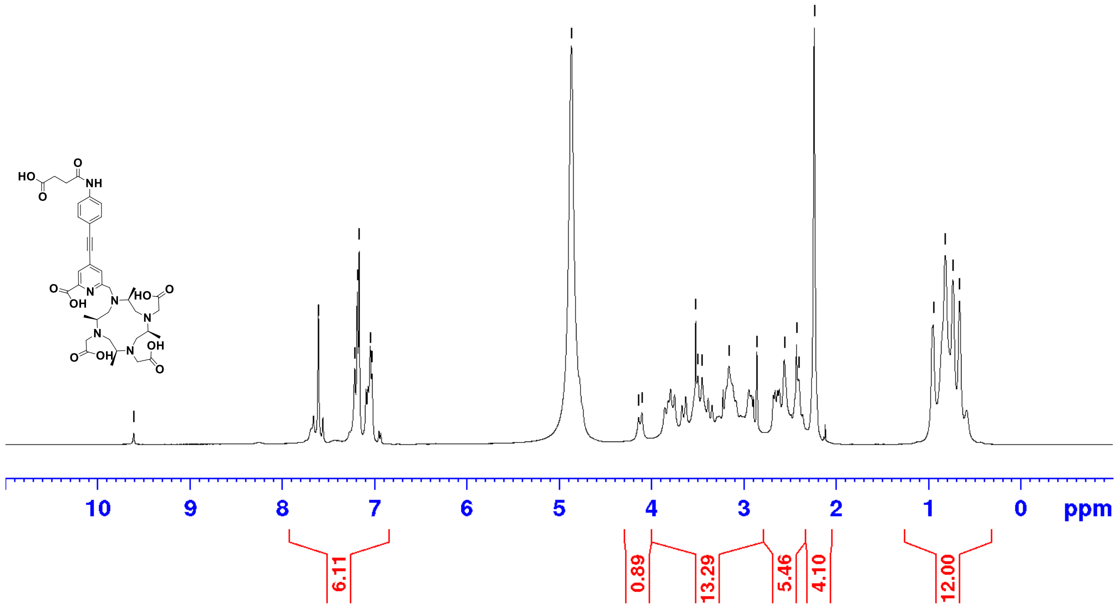
HO
O
NH
O
OH
N
N
HO
O
N
OH
N
OH
O
10
9
8
7
6
5
4
3
2
1
0
ppm
6.11
0.89
13.29
5.46
4.10
12.00

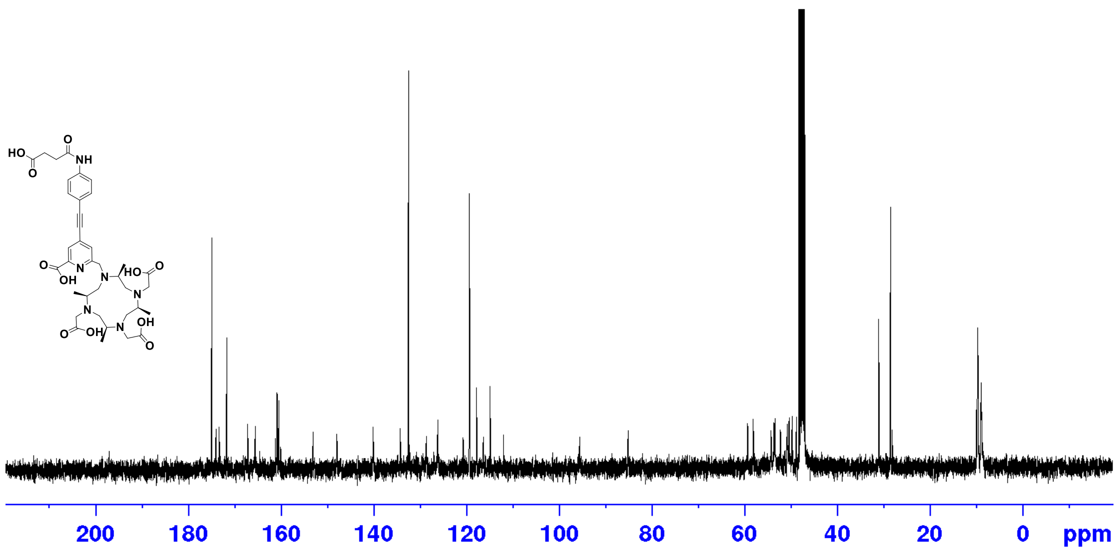
HO
O
NH
O
N
OH
HO
O
N
N
N
O
OH
OH
O
200
180
160
140
120
100
80
60
40
20
0
ppm

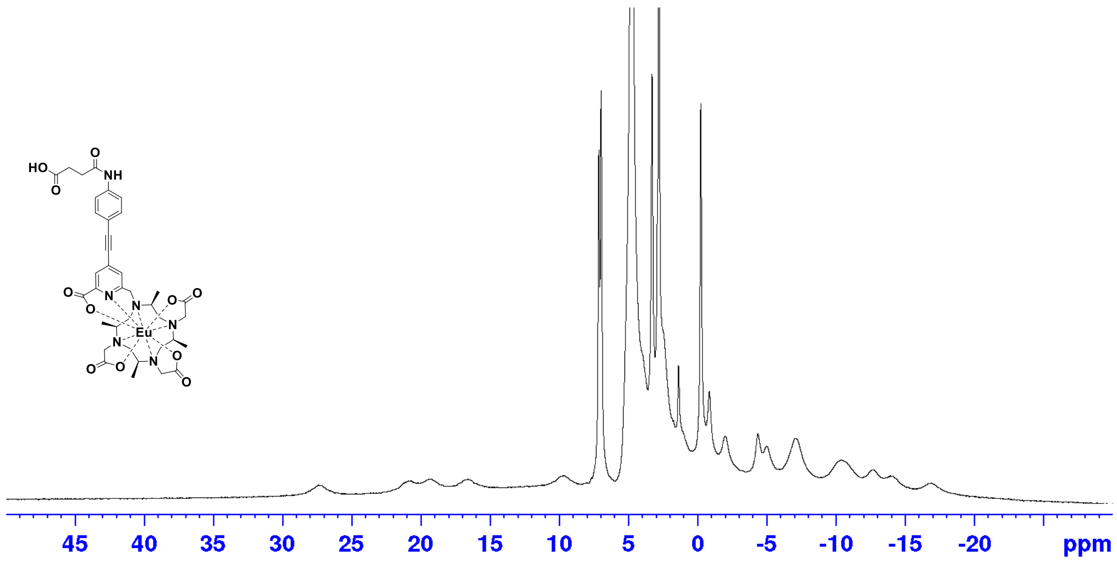
HO
O
NH
O
N
O
O
Eu
N
N
N
N
O
O
O
O
O
O
45
40
35
30
25
20
15
10
5
0
-5
-10
-15
-20
ppm

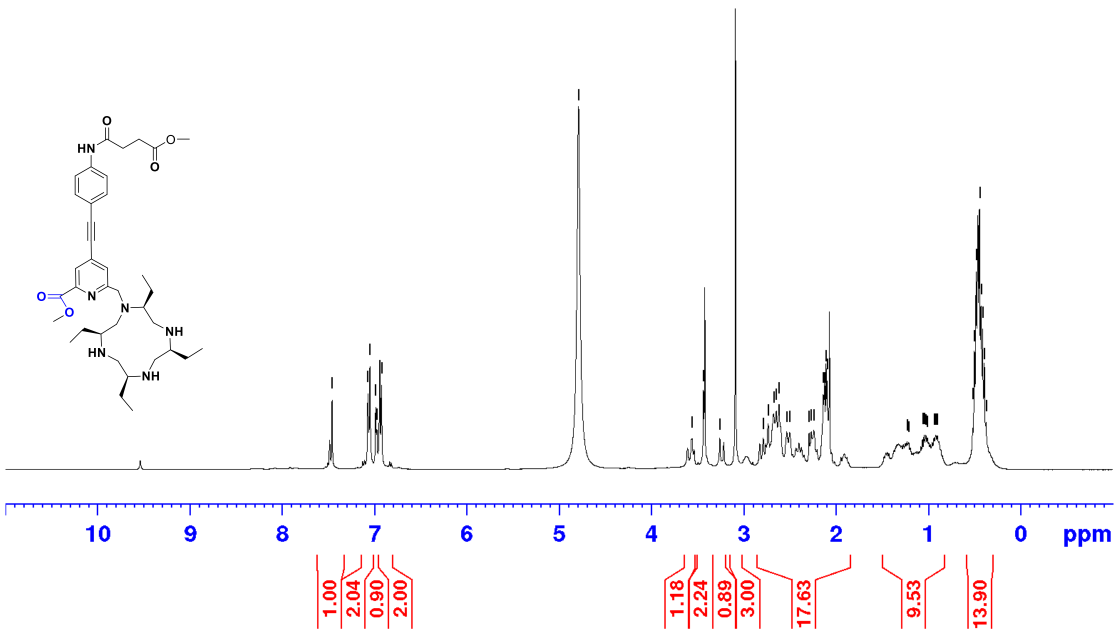
HN
O
O
O
N
O
O
N
NH
HN
NH
10
9
8
7
6
5
4
3
2
1
0
ppm
1.00
2.04
0.90
2.00
1.18
2.24
0.89
3.00
17.63
9.53
13.90

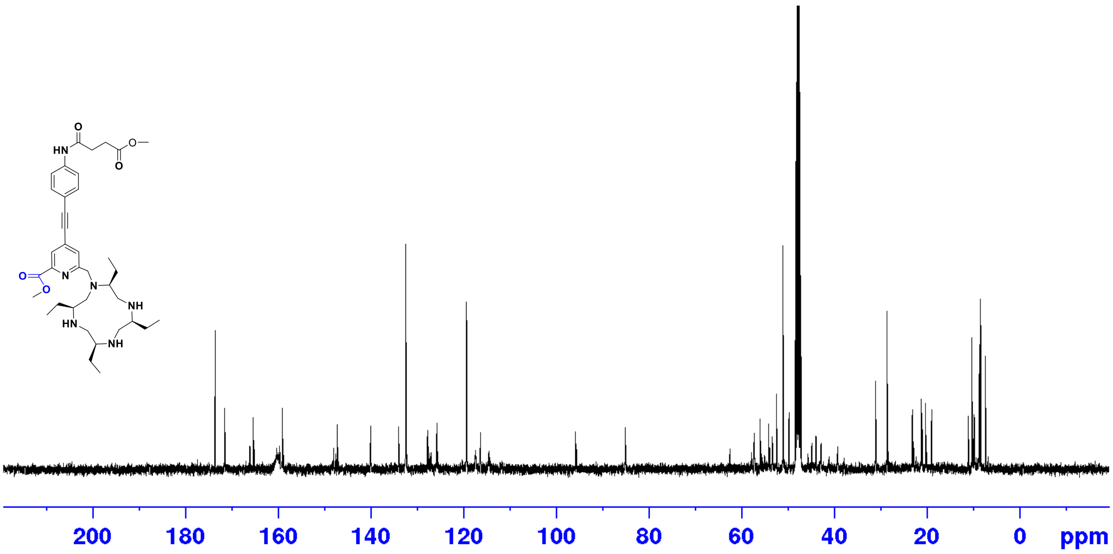
HN
O
O
O
N
N
NH
HN
NH
200
180
160
140
120
100
80
60
40
20
0
ppm

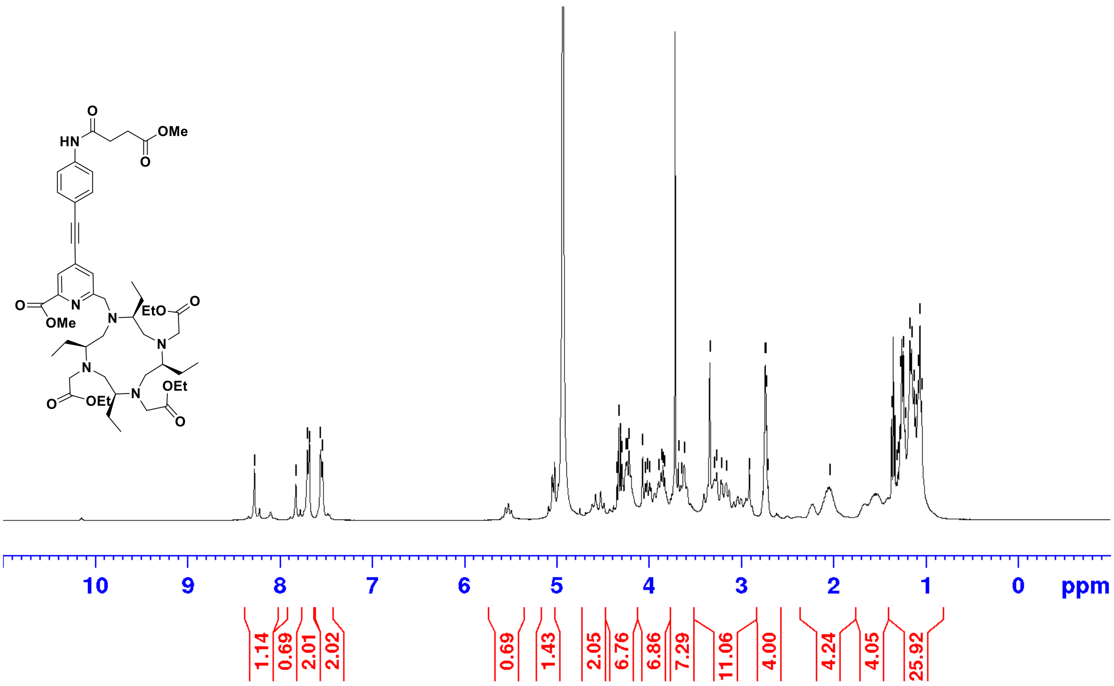
O
HN
OMe
O
N
O
OMe
EtO
O
N
N
N
N
O
OEt
OEt
O
10
9
8
7
6
5
4
3
2
1
0
ppm
1.14
0.69
2.01
2.02
0.69
1.43
2.05
6.76
6.86
7.29
11.06
4.00
4.24
4.05
25.92

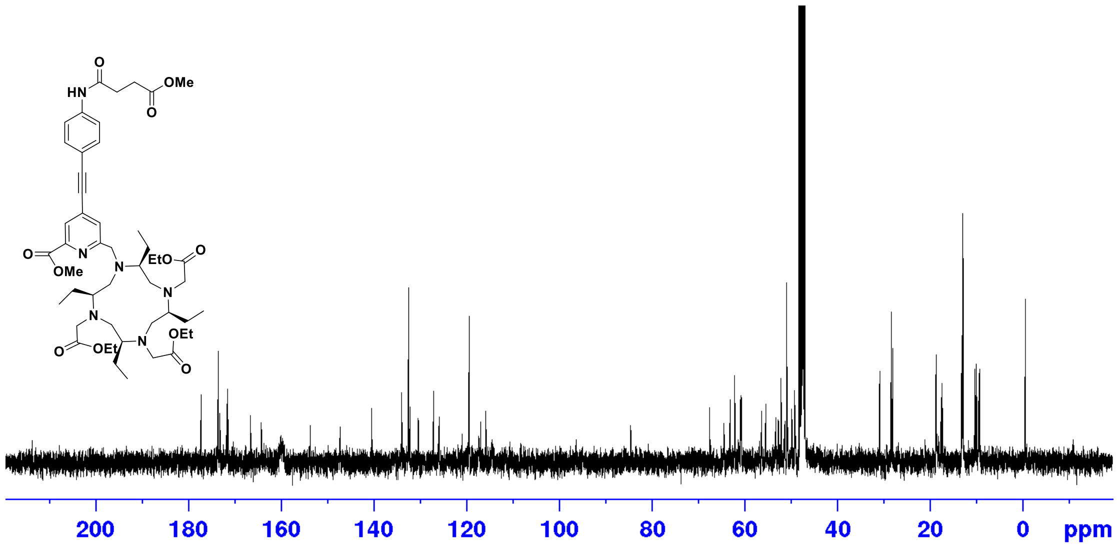

HN
O
OMe
O
N
O
OMe
N
EtO
O
N
N
N
O
OEt
OEt
O
200
180
160
140
120
100
80
60
40
20
0
ppm

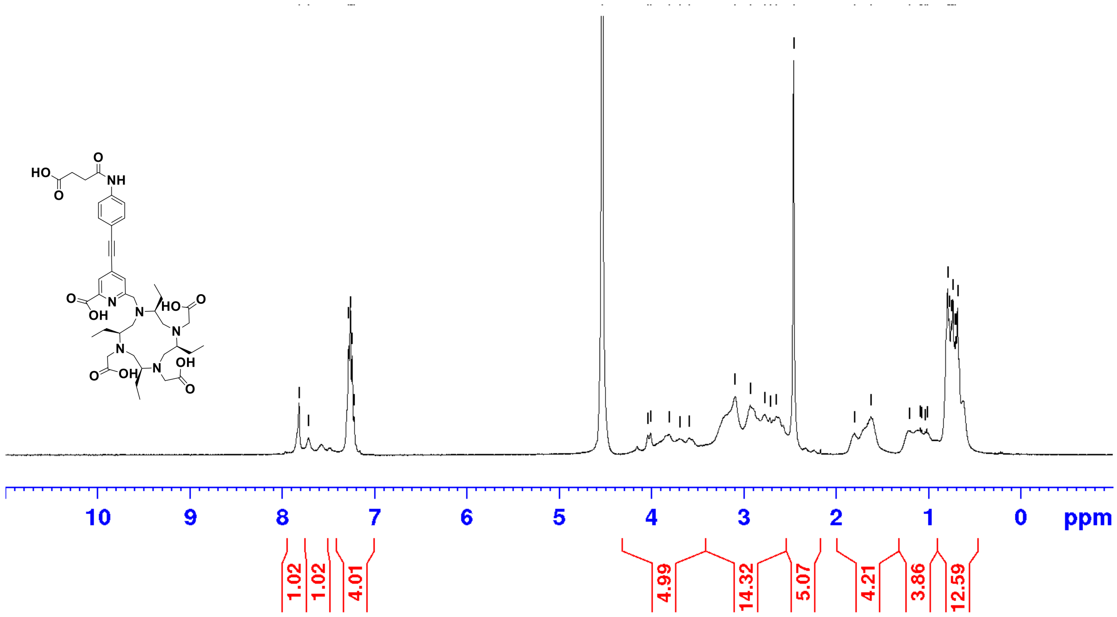

HO
O
O
NH
O
OH
N
N
HO
O
N
N
N
O
OH
OH
O
10
9
8
7
6
5
4
3
2
1
0
ppm
1.02
1.02
4.01
4.99
14.32
5.07
4.21
3.86
12.59

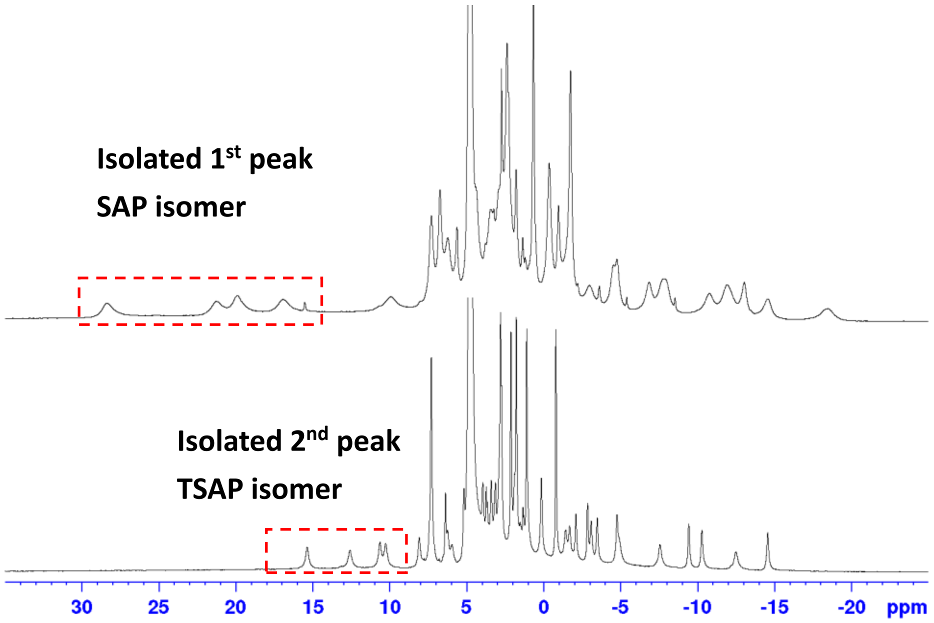
Isolated 1st peak
SAP isomer
Isolated 2nd peak
TSAP isomer
30
25
20
15
10
5
0
-5
-10
-15
-20
ppm

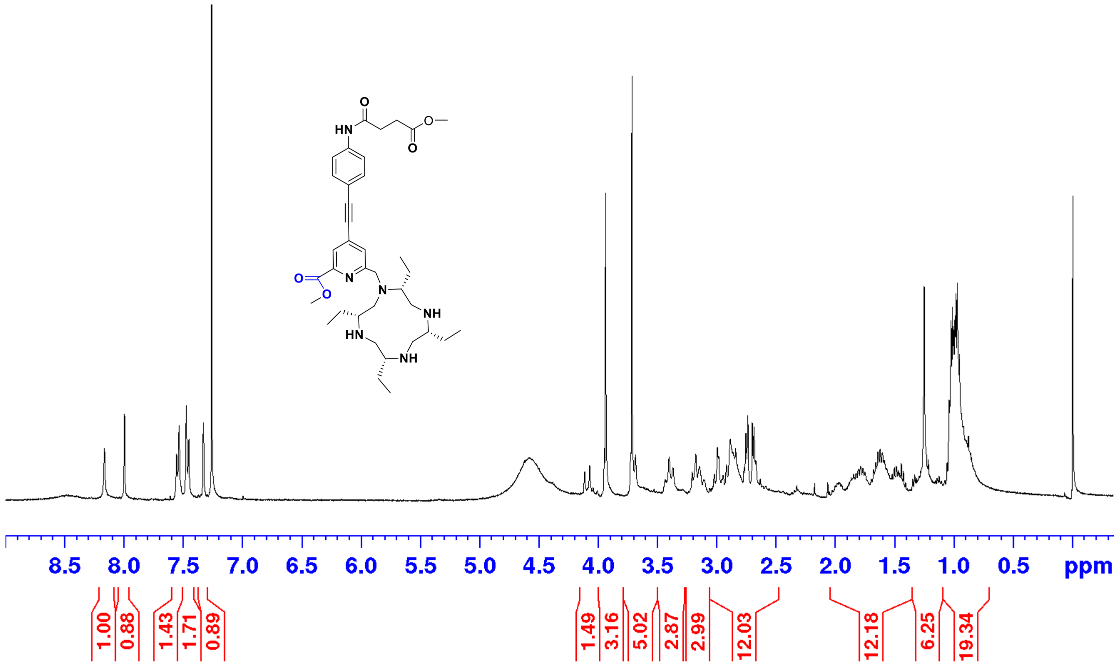
O
HN
O
O
N
O
O
N
NH
HN
NH
8.5
8.0
7.5
7.0
6.5
6.0
5.5
5.0
4.5
4.0
3.5
3.0
2.5
2.0
1.5
1.0
0.5
ppm
1.00
0.88
1.43
1.71
0.89
1.49
3.16
5.02
2.87
2.99
12.03
12.18
6.25
19.34

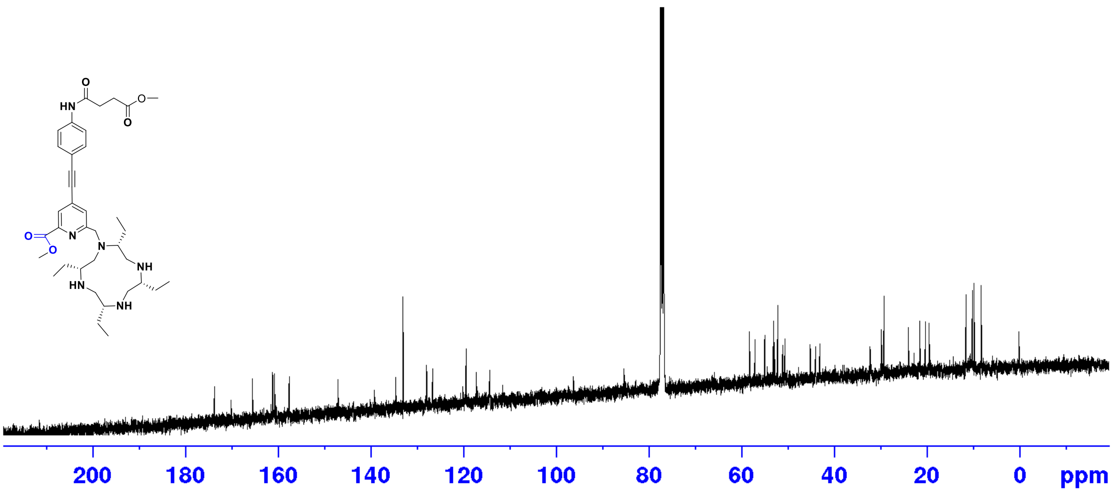
200
180
160
140
120
100
80
60
40
20
0
ppm

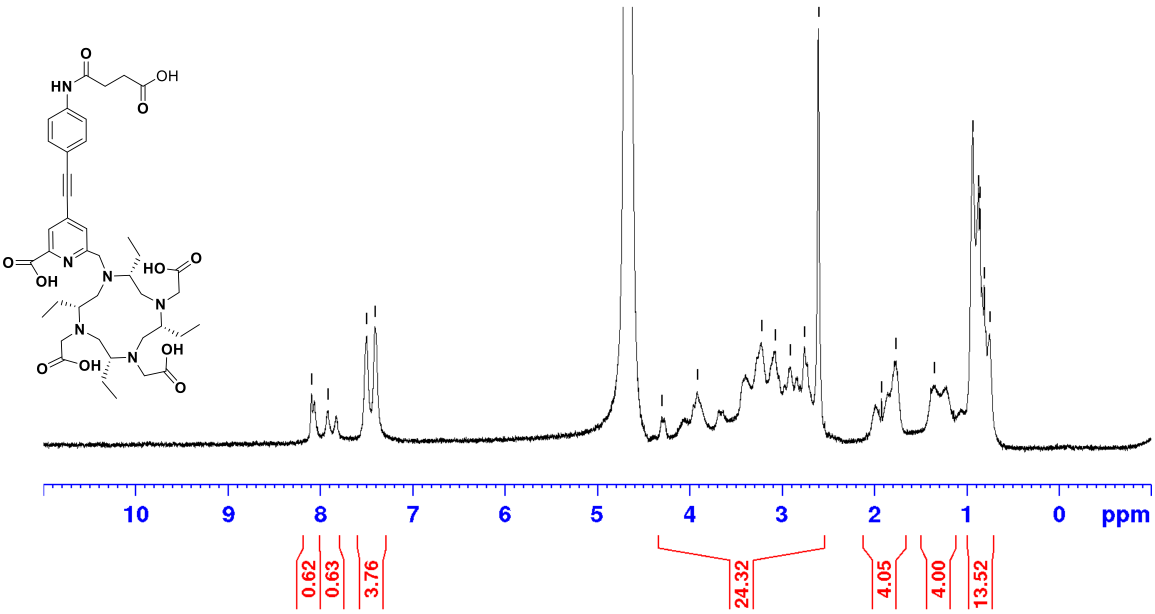
10
9
8
7
6
5
4
3
2
1
0
ppm
0.62
0.63
3.76
24.32
4.05
4.00
13.52

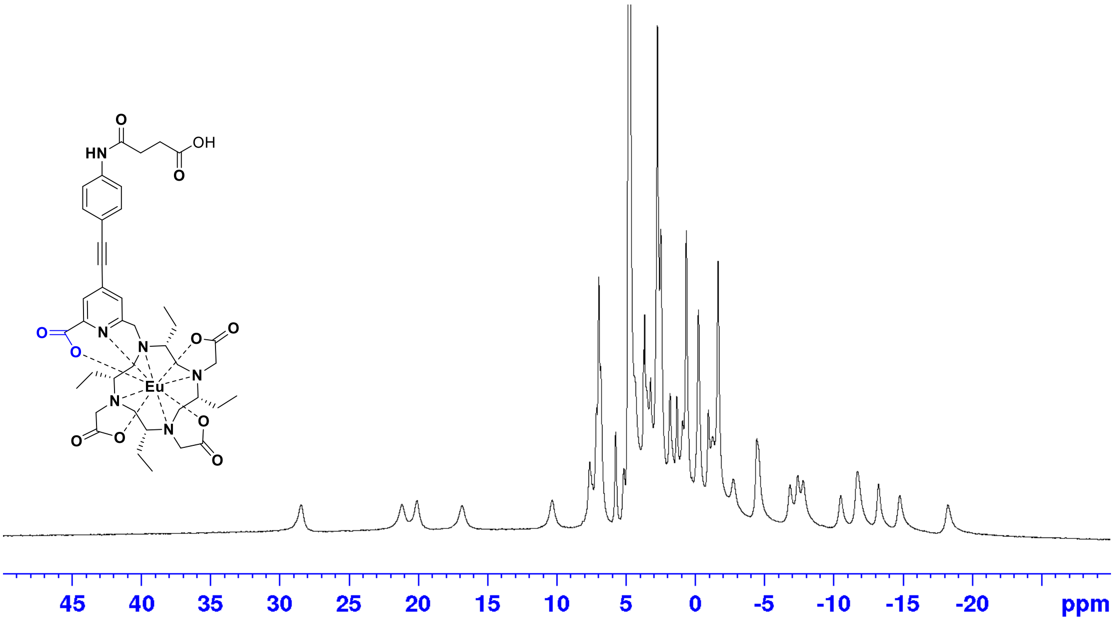
Eu
45
40
35
30
25
20
15
10
5
0
-5
-10
-15
-20
ppm

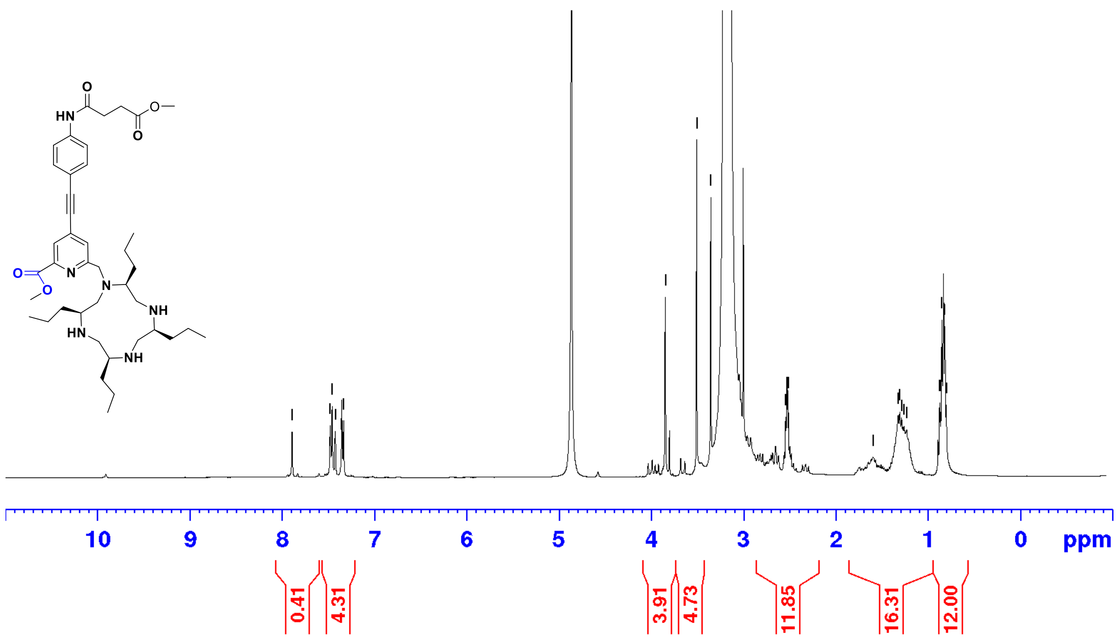

O
HN
O
O
O
N
O
O
N
NH
HN
NH
10
9
8
7
6
5
4
3
2
1
0
ppm
0.41
4.31
3.91
4.73
11.85
16.31
12.00

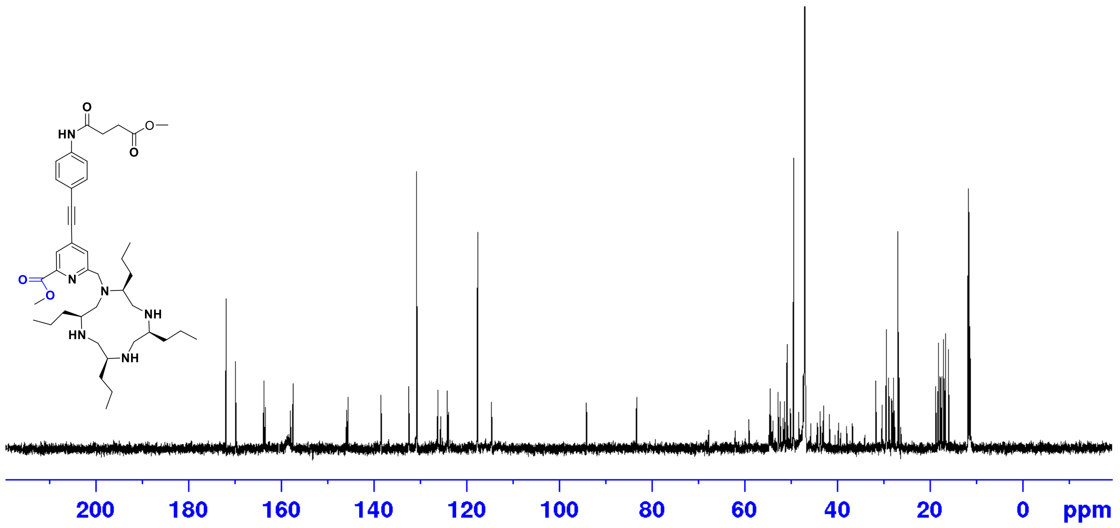

O
HN
O
O
O
N
O
O
N
NH
HN
NH
200
180
160
140
120
100
80
60
40
20
0
ppm

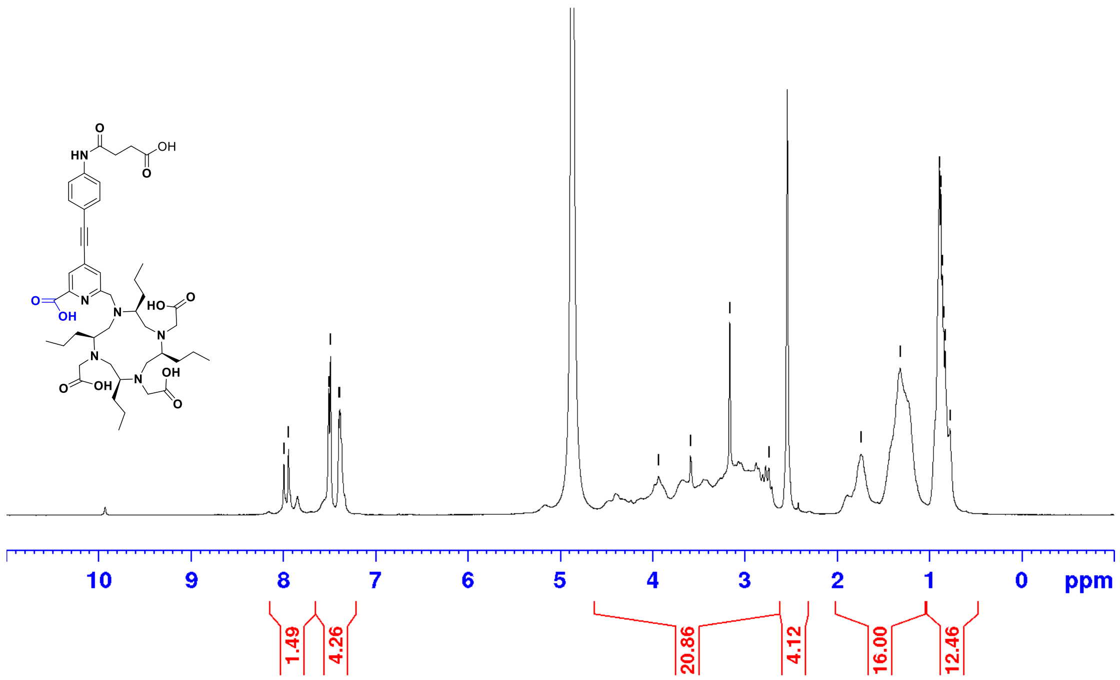
HN
O
OH
O
O
OH
N
N
HO
O
N
N
N
O
OH
OH
O
10
9
8
7
6
5
4
3
2
1
0
ppm
1.49
4.26
20.86
4.12
16.00
12.46

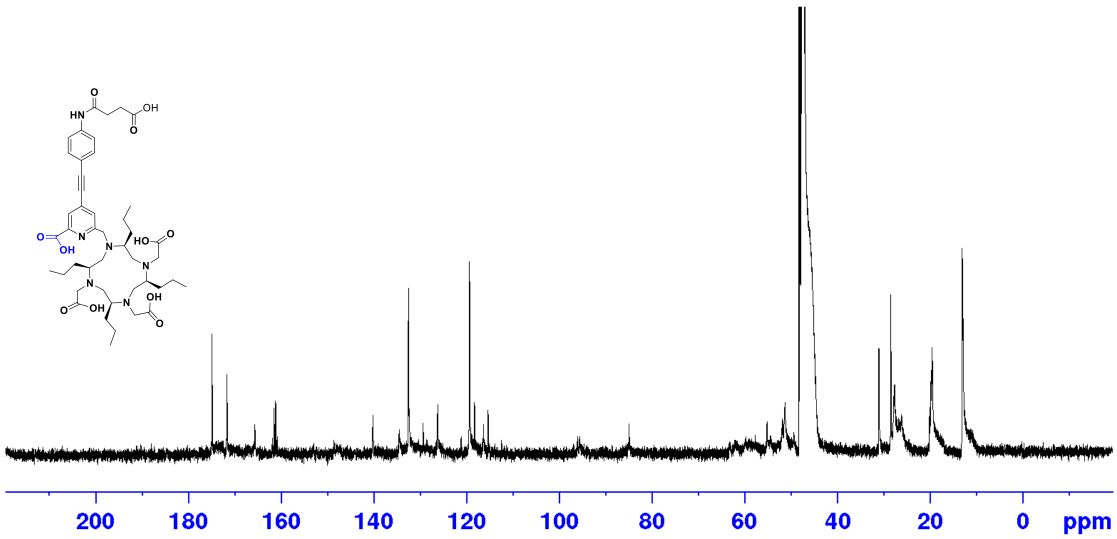
HN
O
OH
O
O
OH
N
N
HO
O
N
N
N
O
OH
OH
O
200
180
160
140
120
100
80
60
40
20
0
ppm

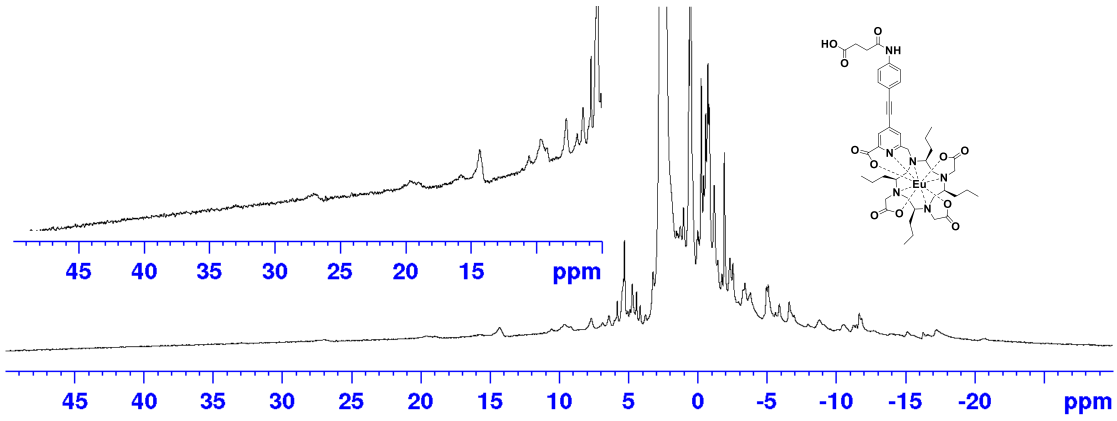
HO
O
O
NH
O
O
N
N
Eu
N
N
N
O
O
O
O
O
O
45
40
35
30
25
20
15
ppm
45
40
35
30
25
20
15
10
5
0
-5
-10
-15
-20
ppm

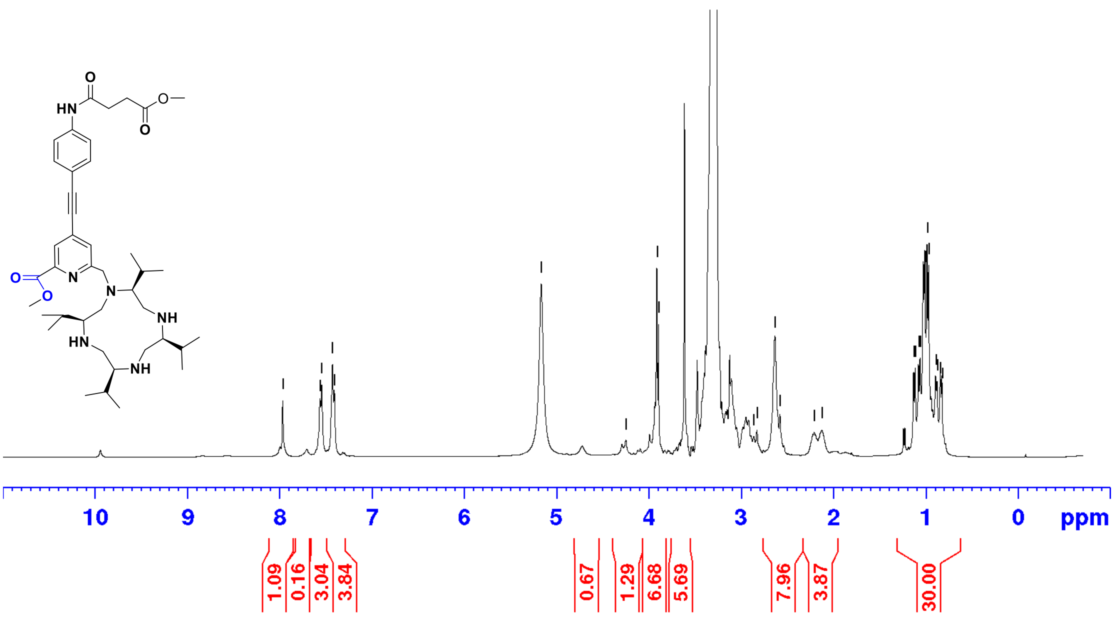
10 9 8 7 6 5 4 3 2 1 0 ppm
1.09 0.16 3.04 3.84 0.67 1.29 6.68 5.69 7.96 3.87 30.00

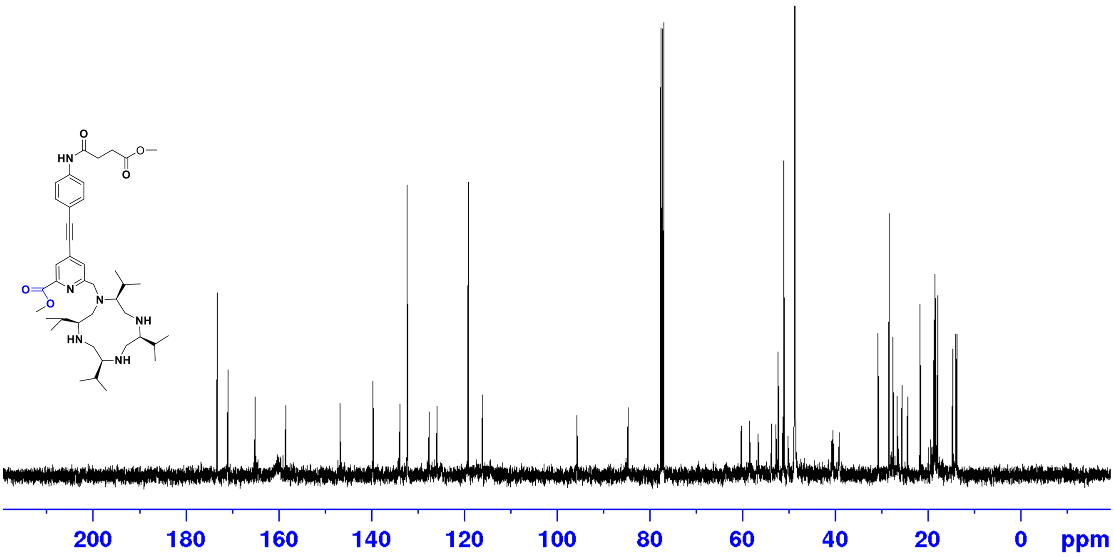
200 180 160 140 120 100 80 60 40 20 0 ppm

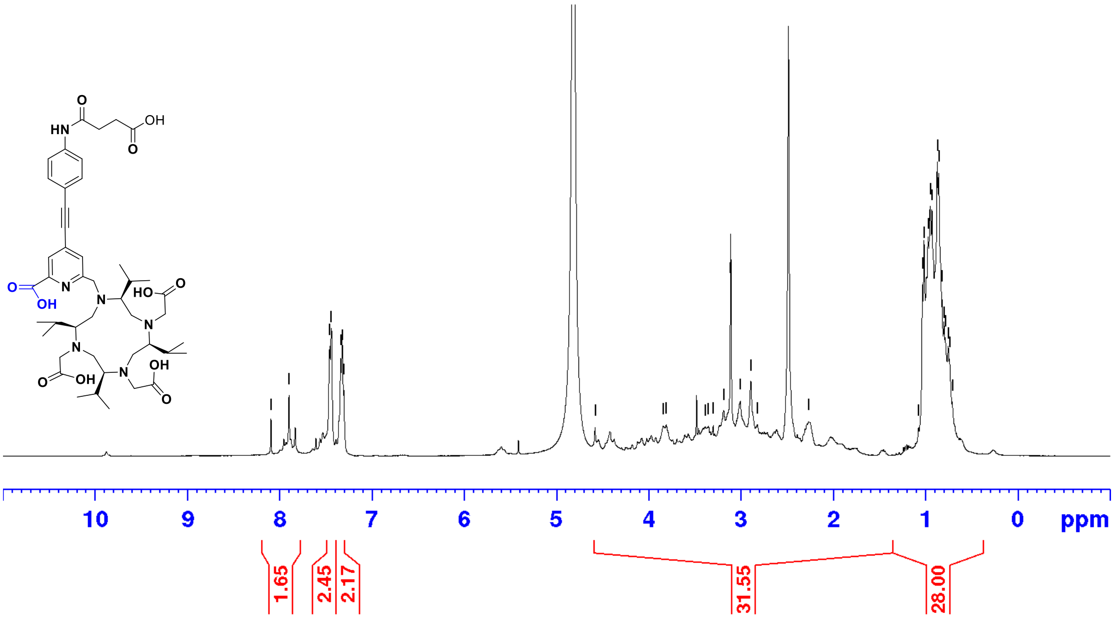
10 9 8 7 6 5 4 3 2 1 0 ppm
1.65 2.45 2.17 31.55 28.00

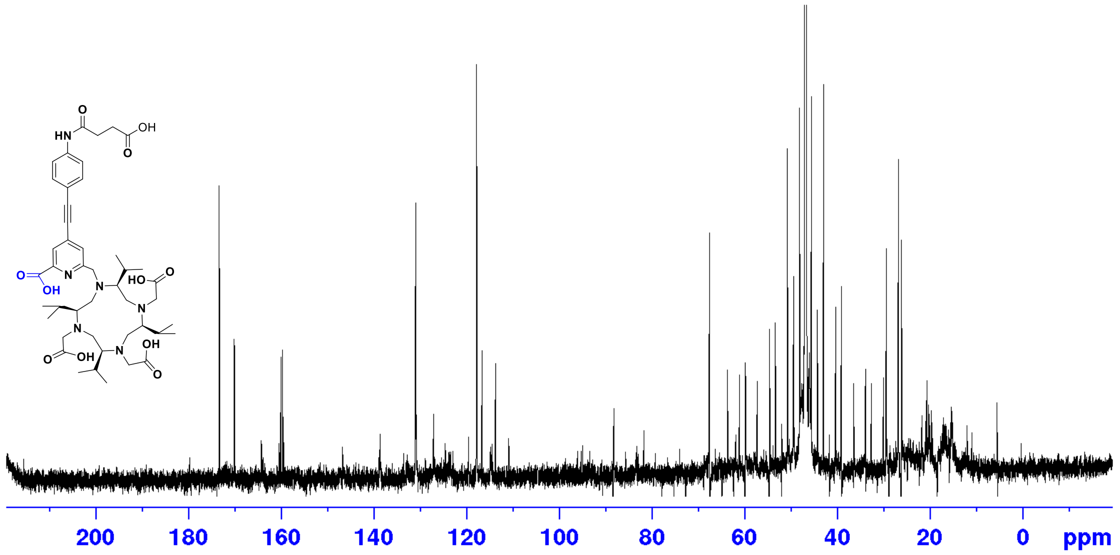
200 180 160 140 120 100 80 60 40 20 0 ppm

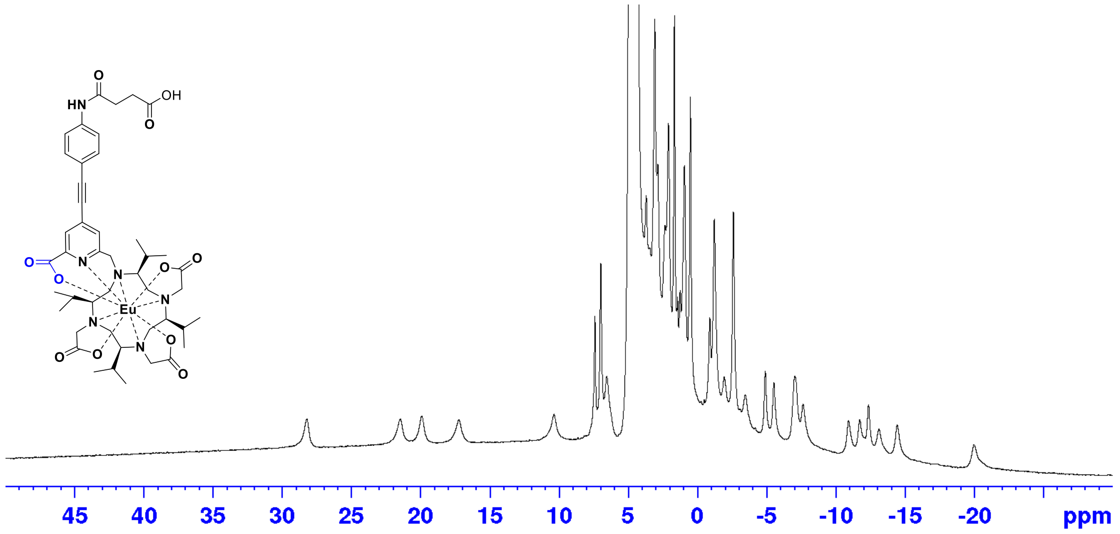
45 40 35 30 25 20 15 10 5 0 -5 -10 -15 -20 ppm

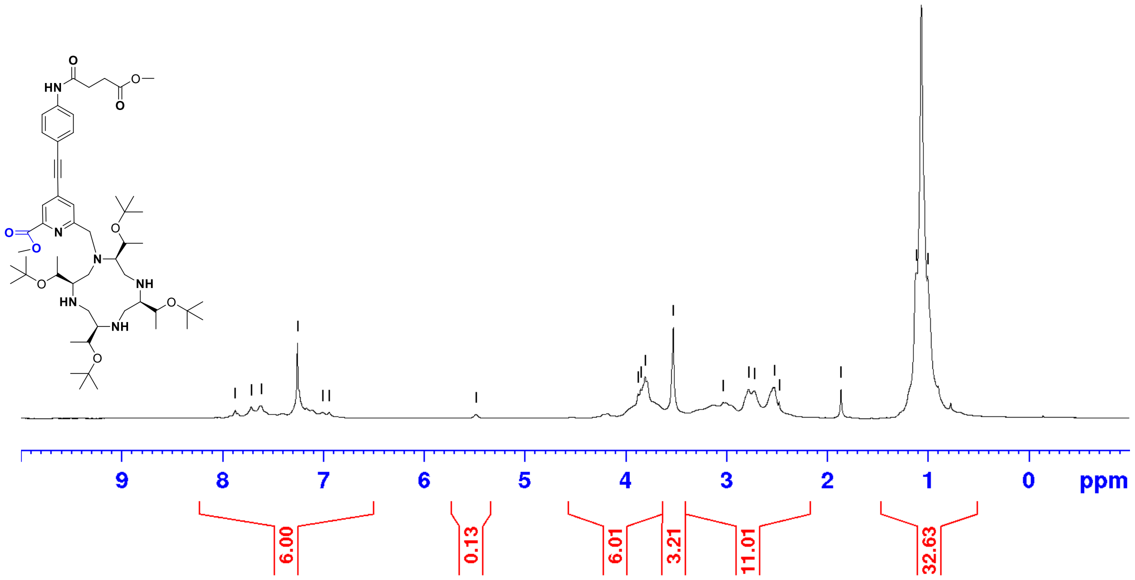
9 8 7 6 5 4 3 2 1 0 ppm
6.00
0.13
6.01
3.21
11.01
32.63

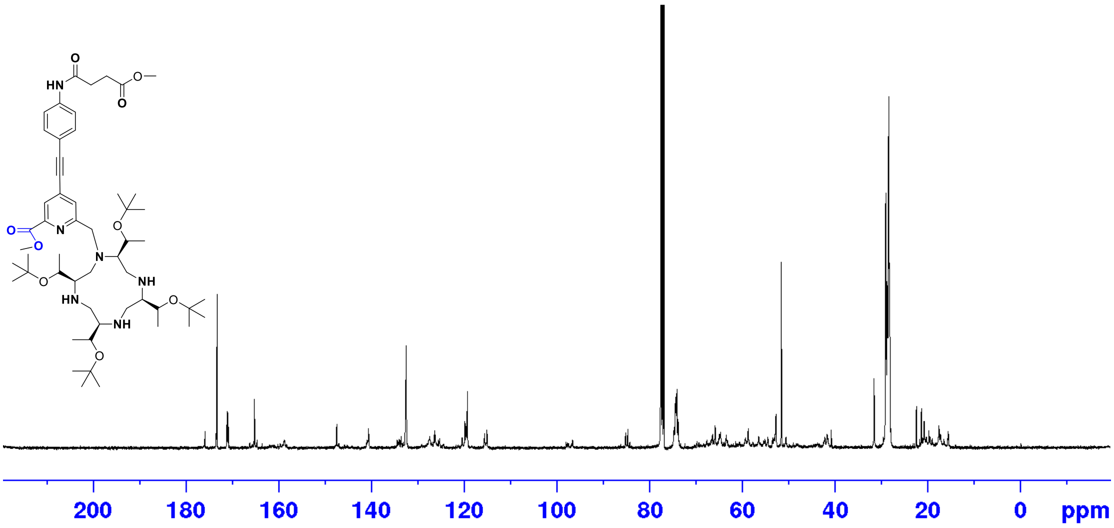
200 180 160 140 120 100 80 60 40 20 0 ppm

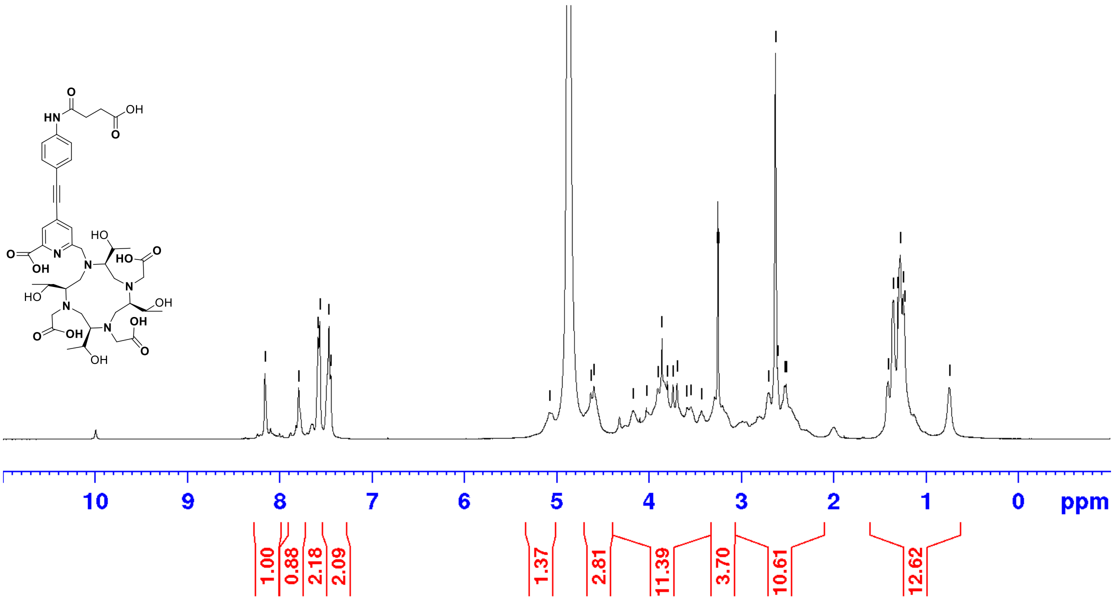
10 9 8 7 6 5 4 3 2 1 0 ppm
1.00 0.88 2.18 2.09 1.37 2.81 11.39 3.70 10.61 12.62

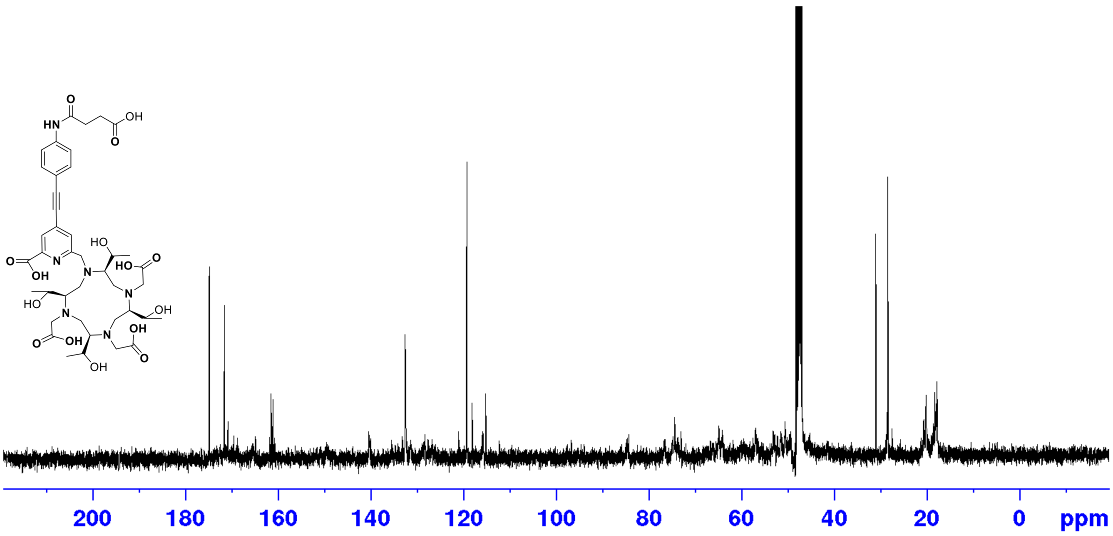
200 180 160 140 120 100 80 60 40 20 0 ppm

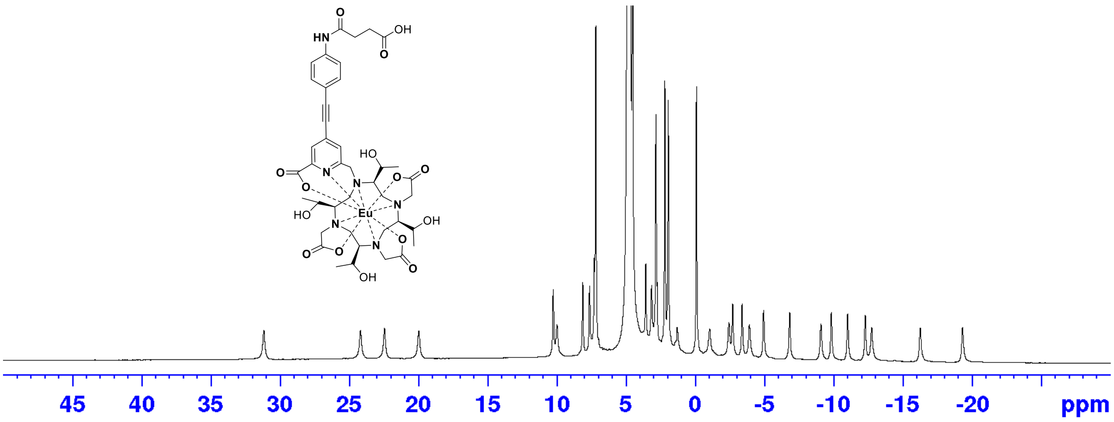

8. References

[1] J. R. Lakowicz, *Principles of Fluorescence Spectroscopy,* Springer*,* **2006**.

[2] A. Aebischer, F. Gumy, J.-C. G. Bünzli, *Phys. Chem. Chem. Phys.* **2009**, *11*, 1346-1353.

[3] A. Beeby, I. M. Clarkson, R. S. Dickins, S. Faulkner, D. Parker, L. Royle, A. S. de Sousa, J. A. G. Williams, M. Woods, *J. Chem. Soc.* **1999**, 493-503.

[4] R. M. Supkowski, J. W. D. Horrocks, *Inorg. Chim. Acta.* **2002**, *340*, 44-48.

[5] T. Harada, H. Hayakawa, M. Watanabe, M. Takamoto, *Rev. Sci. Instrum.* **2016**, *87*, 075102.

[6] L. Krause, R. Herbst-Irmer, G. M. Sheldrick, D. Stalke, *J. Appl. Crystallogr.* **2015**, *48*, 3-10.

[7] Bruker-AXS. APEX3 Software Suite. Madison, Wisconsin (USA), **2014**.

[8] C. B. Hubschle, G. M. Sheldrick, B. Dittrich, *J. Appl. Crystallogr.* **2011**, *44*, 1281-1284.

[9] J. W. Walton, A. Bourdolle, S. J. Butler, M. Soulie, M. Delbianco, B. K. McMahon, R. Pal, H. Puschmann, J. M. Zwier, L. Lamarque, O. Maury, C. Andraud, D. Parker, *Chem. Commun.* **2013**, *49*, 1600-1602.

[10] L. Dai, W. S. Lo, I. D. Coates, R. Pal, G. L. Law, *Inorg. Chem.* **2016**, *55*, 9065-9070.

[11] L. Dai, C. M. Jones, W. T. K. Chan, T. A. Pham, X. Ling, E. M. Gale, N. J. Rotile, W. C.-S. Tai, C. J. Anderson, P. Caravan, G.-L. Law, *Nat. Commun.* **2018**, *9*, 857.

[12] A. M. Webster, S. L. Cobb, *Tetrahedron Lett.* **2017**, *58*, 1010-1014.
